# Supplementary material for: Hippo signaling regulates cuticle pigmentation and dopamine metabolism in Drosophila
Source: PLoS Genet. 2026 Aug 21;22(8):e1012260. doi: 10.1371/journal.pgen.1012260 (PMC13524344; doi:10.1371/journal.pgen.1012260)
Supplement: Supplementary file 1 [file pgen.1012260.s011.pdf]

S2 Table: List of transgenic *Drosophila* lines used and additional information.

LINE	GENOTYPE	SOURCE	STOCK NUMBER
pnr-Gal4	*yw;P{GawB}pnr*^{MD237}*/TM3,Sb*	BDSC<br>*rebalanced with TM3,Sb	3039
pnr-Gal4, UAS-TH-RNAi	*yw;P{GawB}pnr*^{MD237}*,P{TRiP.JF01813}attP2/TM3,Sb*	This study (recombined BDSC 3039 and BDSC 25796)	
TH-Gal4	*w***; P{ple-GAL4.F}3*	BDSC	8848
R58-Gal4	*w*^{1118}*; P{GMR58E02-GAL4}attP2*	BDSC	41347
TH-Gal4, R58-Gal4	*yw; P{ple-GAL4.F}3, P{GMR58E02-GAL4}attP2*	Deal et. al [44]	
tub-Gal80^{ts}	*w*; P{tubP-GAL80[ts]}10; TM2/TM6B,Tb*^{1}	BDSC	7108
tub-Gal80^{ts}; pnr-Gal4	*w*; P{tubP-GAL80[ts]}10/CyO; P{GawB}pnr*^{MD237}*/TM6B,Tb*^{1}	This study (double balanced BDSC 7108 and BDSC 3039)	
tub-Gal80^{ts}; pnr-Gal4, UAS-TH-RNAi	*w*; P{tubP-GAL80[ts]}10/CyO; P{GawB}pnr*^{MD237}*,P{TRiP.JF01813} attP2/TM3,Sb*	This study (double balanced BDSC 7108 and recombined BDSC 3039 and BDSC 25796)	
UAS-Cas9; pnr-Gal4	*w*; P{UAS-Cas9.P2}attP40/ CyO;P{GawB}pnr*^{MD237}*/TM6B,Tb*^{1}	BDSC	67077
UAS-Cas9; pnr-Gal4, UAS-TH-RNAi	*y*^{1}*w*; P{UAS-Cas9.P2}attP40/CyO; P{GawB}pnr*^{MD237}*,P{TRiP.JF01813} attP2/TM3,Sb*	This study	
wts[TG4]	*y*^{1}*w*; Mi{Trojan-GAL4.0} wts[MI05605-TG4.0]/TM3,Sb*	This study	
yki[TG4]	*y*^{1}*w*;TI{GFP[3xP3.cLa]=CRIMIC.TG4.1}yki[CR00812-TG4.1]/SM6a*	BDSC	79628
pnr-Gal4, UAS-CD8::GFP	*y*^{1}*w*; pnr-Gal4, P{w[+mC]=UAS-mCD8::GFP.L}LL6/TM3,Sb*	This study (recombined BDSC 3039 and BDSC 5130)	
UAS-Ctrl-RNAi	*w P{w*$^{+}$*, UAS>lacZ.SL.IR}12a*	Kennerdell and Carthew [152]	
UAS-TH-RNAi	*y*^{1}*v*^{1} *; P{TRiP.JF01813}attP2*	BDSC	25796
UAS-hpo-RNAi^{1}	*y*^{1}*v*^{1} *; P{TRiP.HMS00006}attP2*	BDSC	33614
UAS-hpo-RNAi^{2}	*y*^{1}*v*^{1} *; P{TRiP.JF02740}attP2*	BDSC	27661
UAS-hpo-RNAi^{3}	w^{1118}; P{GD1570}v7823	VDRC	v7823
UAS-mats-RNAi^{1}	*y*^{1}*sc*v*^{1}*sev*^{21} *; P{TRiP.HMS00475}attP2*	BDSC	34959
UAS-mats-RNAi^{2}	*y*^{1}*sc*v*^{1}*sev*^{21} *; P{TRiP.JF03246}attP2*	BDSC	29567
UAS-mats-RNAi^{3}	*w*^{1118}*; P{GD8136}v17716*	VDRC	v17716
UAS-wts-RNAi^{1}	*P{NIG.12072R}2*	NIG-Japan	12702R-2
UAS-wts-RNAi^{2}	*P{NIG.12072R}1*	NIG-Japan	12702R-1
UAS-wts-RNAi^{3}	*w*^{1118}*; P{GD1563}v9928*	VDRC	v9928
UAS-yki-RNAi^{1}	*P{NIG.4005R}3*	NIG-Japan	4005R-3
UAS-yki-RNAi^{2}	*w*^{1118}*; P{GD11187}v40497/TM3*	VDRC	v40497
UAS-sd-RNAi^{1}	*y*^{1}*v*^{1}*; P{TRiP.JF02514}attP2*	BDSC	29352
UAS-sd-RNAi^{2}	*y*^{1}*sc*v*^{1}*sev*^{21} *; P{TRiP.GL00410}attP2*	BDSC	35481
UAS-bsk-RNAi	*y*^{1} *v*^{1}*; P{TRiP.JF01275}attP2*	BDSC	31323
UAS-hep-RNAi	*y*^{1}*sc*v*^{1}*sev*^{21} *; P{ TRiP.GL00089}attP2*	BDSC	35210
UAS-stw-RNAi	*w*^{1118}*; P{GD12744}v22959*	VDRC	v22959
UAS-Lk6-RNAi	*w*^{1118}*; P{GD9353}v32885*	VDRC	v32885
UAS-yellow-RNAi	*y*^{1}*sc*v*^{1}*sev*^{21} *; P{TRiP.HMC05546}attP40*	BDSC	64527
UAS-Bsg-RNAi	*w*^{1118}*; P{GD15718}v43306*	VDRC	v43306
UAS-stv-RNAi	*w*^{1118}*; P{GD10796}v34408*	VDRC	v34408

UAS-bab1-RNAi	*y^{1}sc*v^{1}sev^{21}; P{TRiP.HMC04716}attP40*	BDSC	57410
UAS-cib-RNAi	*y^{1}v^1; P{TRiP.JF02837}attP2*	BDSC	28003
UAS-taldo-RNAi	*P{NIG.2827R}2*	NIG-Japan	2827R-2
UAS-Duox-RNAi	*y^{1}sc*v^{1}sev^{21}; P{TRiP.HMS00692}attP2*	BDSC	32903
UAS-Gp150-RNAi	*P{NIG.5820R}2*	NIG-Japan	5802R-2
UAS-InR-RNAi	*y^1 v^1; P{TRiP.JF01183}attP2*	BDSC	31594
UAS-mgl-RNAi	*y^1 v^1; P{TRiP.JF02545}attP2*	BDSC	27242
UAS-yellow-B-RNAi	*P{NIG.17914R}1*	NIG-Japan	17914R-1
UAS-kibra-RNAi	*y^1 v^1; P{TRiP.JF03098}attP2*	BDSC	28683
EGFP-gRNA	*y^{1}sc*v^{1}sev^{21}; P{TKO.GS00382}attP40*	BDSC	79393
hpo-gRNA	*y^{1}sc*v^{1}sev^{21}; P{TKO.GS04500}attP40*	BDSC	85878
sav-gRNA	*y^{1}sc*v^{1}sev^{21};* P{TKO.GS05550}attP40/CyO	BDSC	84080
UAS-LacZ	*w*; P{UAS-lacZ.Exel}2*	BDSC	8529
UAS-TH	*w*; P{UAS-ple.T}331f2*	BDSC	37539
UAS-yki.S168A^1	*y^{1}w*; P{UAS-yki.S168A.GFP.HA}10-7-Y*	BDSC	28816
UAS-yki.S168A^2	*w*; P{UAS-yki.S168A.V5}attP2*	BDSC	28818
UAS-yki.S3	*w*; P{UAS-yki.S111A.S168A.S250A.V5}attP2*	BDSC	28817
UAS-MOB1A	*yw; pGW.UAS-MOB1A*	This study	
UAS-MOB1B	*yw; pGW.MOB1B*	This study	
UAS-LATS1	*yw; pGW.UAS-LATS1*	This study	
UAS-LATS2	*yw; pGW.UAS-LATS2*	This study	
UAS-yki	*w*; P{UAS-yki.V5.O}attP2*	BDSC	28819
UAS-YAP1	*y^{1}w*; pGW.UAS-YAP1*	This study	
UAS-yellow	*y^{1}w^{1118}; P{UAS-y.C}MC1*	BDSC	3043
UAS-Ddc	*w*; P{UAS-Ddc.T}16f2/TM3,Sb^1*	BDSC	37540
UAS-TH, UAS-Ddc	*y^{1}w*; P{UAS-ple.T}331f2,P{UAS-Ddc.T}16f2/TM3, Sb*	This study (recombined BDSC 37539 and BDSC 37540)	

*BDSC=Bloomington Drosophila Stock Center, VDRC=Vienna Drosophila Resource Center, NIG= National Institute of Genetics
