# Supplementary material for: Hippo signaling regulates cuticle pigmentation and dopamine metabolism in Drosophila
Source: PLoS Genet. 2026 Aug 21;22(8):e1012260. doi: 10.1371/journal.pgen.1012260 (PMC13524344; doi:10.1371/journal.pgen.1012260)
Supplement: Supplementary file 2 [file pgen.1012260.s012.pdf]

S3 Table: *Drosophila* lines that produced no obvious change of *wts* KD phenotypes.

DEG PHENOTYPE	LINE	GENOTYPE	SOURCE	STOCK NUMBER
Concordant	UAS-CG46639	y^{1}sc*v^{1}sev^{21} ; P{ TRiP.HMS01636}attP2	BDSC	37494
Concordant	UAS-Hrb98DE-RNAi^{1}	w^{1118}; P{GD14939}v29523	VDRC	v29523
Concordant	UAS-Hrb98DE-RNAi^{2}	P{NIG.9983R}4	NIG-Japan	9983R-4
Concordant	UAS-Mp-RNAi^{1}	y^{1}v^{1}; P{ TRiP.JF02929}attP2	BDSC	28299
Concordant	UAS-Mp-RNAi^{2}	y^{1}v^{1}; P{TRiP.HMJ21668}attP40	BDSC	52981
Concordant	UAS-Mp-RNAi^{3}	P{NIG.8647R}2	NIG-Japan	8647R-2
Concordant	UAS-Mp-RNAi^{4}	P{NIG.8647R}3	NIG-Japan	8647R-3
Concordant	UAS-sima^{1}	y^{1}v^{1}; P{TRiP.JF02105}attP2	BDSC	26207
Concordant	UAS-sima^{2}	y^{1}sc*v^{1}sev^{21} ; P{TRiP.HMS00832}attP2	BDSC	33894
Concordant	UAS-sima^{3}	P{KK102226}VIE-260B	VDRC	v106187
Concordant	UAS-Synd^{1}	y^{1}v^{1}; P{ TRiP.JF02607}attP2/TM3, Sb[1]	BDSC	27297
Concordant	UAS-Synd^{2}	w^{1118}; P{GD9087}v40018	VDRC	v40018
Concordant	UAS-Synd^{3}	P{KK109082}VIE-260B	VDRC	v104580
Non-Concordant	UAS-cdi-RNAi^{1}	y^{1}v^{1}; P{ TRiP.JF03006}attP2	BDSC	28369
Non-Concordant	UAS-cdi-RNAi^{2}	y^{1}v^{1}; P{TRiP.HMJ02227}attP40	BDSC	42568
Non-Concordant	UAS-cdi-RNAi^{3}	y^{1}sc*v^{1}sev^{21} ; P{TRiP.HMS04453}attP40/CyO	BDSC	57010
Non-Concordant	UAS-cdi-RNAi^{4}	y^{1}sc*v^{1}sev^{21} ; P{TRiP.GL01860}attP2/TM3, Sb[1]	BDSC	57032
Non-Concordant	UAS-CG42671-RNAi^{1}	y^{1}sc*v^{1}sev^{21} ; P{TRiP.GL01098}attP2	BDSC	36844
Non-Concordant	UAS-CG42671-RNAi^{2}	P{KK104428}VIE-260B	BDSC	v100467
Non-Concordant	UAS-CG42674-RNAi^{1}	y^{1}v^{1}; P{TRiP.JF01659}attP2/TM3, Sb[1]	BDSC	31166
Non-Concordant	UAS-CG42674-RNAi^{2}	y^{1}sc*v^{1}sev^{21} ; P{TRiP.HMS00228}attP2	BDSC	34943
Non-Concordant	UAS-Vrp1-RNAi^{1}	y^{1}sc*v^{1}sev^{21} ; P{TRiP.HMS01593}attP2	BDSC	36704
Non-Concordant	UAS-Vrp1-RNAi^{2}	y^{1}sc*v^{1}sev^{21} ; P{ TRiP.GL00640}attP40	BDSC	38201

*BDSC=Bloomington Drosophila Stock Center, VDRC=Vienna Drosophila Resource Center, NIG= National Institute of Genetics
