# Supplementary material for: Hippo signaling regulates cuticle pigmentation and dopamine metabolism in Drosophila
Source: PLoS Genet. 2026 Aug 21;22(8):e1012260. doi: 10.1371/journal.pgen.1012260 (PMC13524344; doi:10.1371/journal.pgen.1012260)

Notum Images

Controls

gRNA Control

Ctrl gRNA; pnr>Cas9 29C

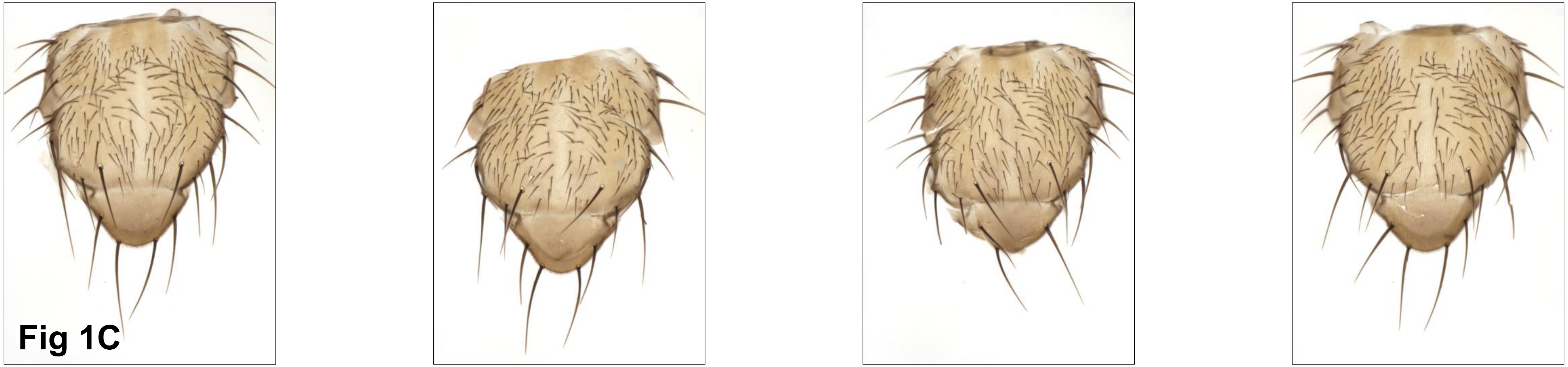

RNAi Controls

pnr>Ctrl-RNAi 29C (1of2)

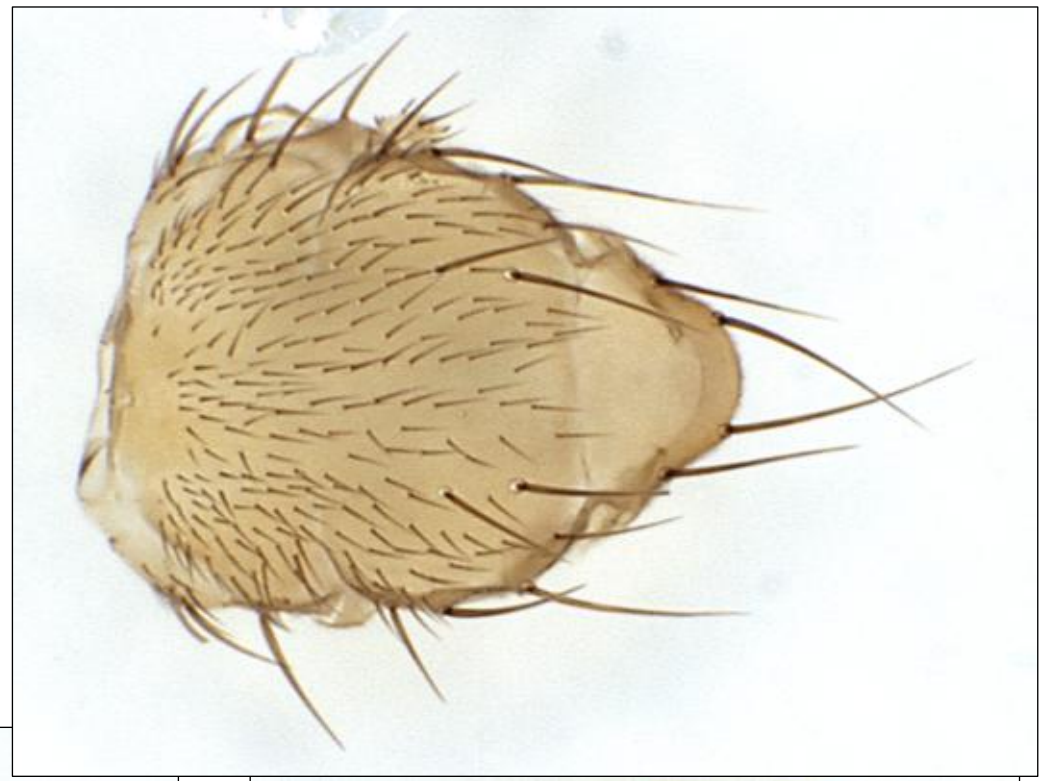

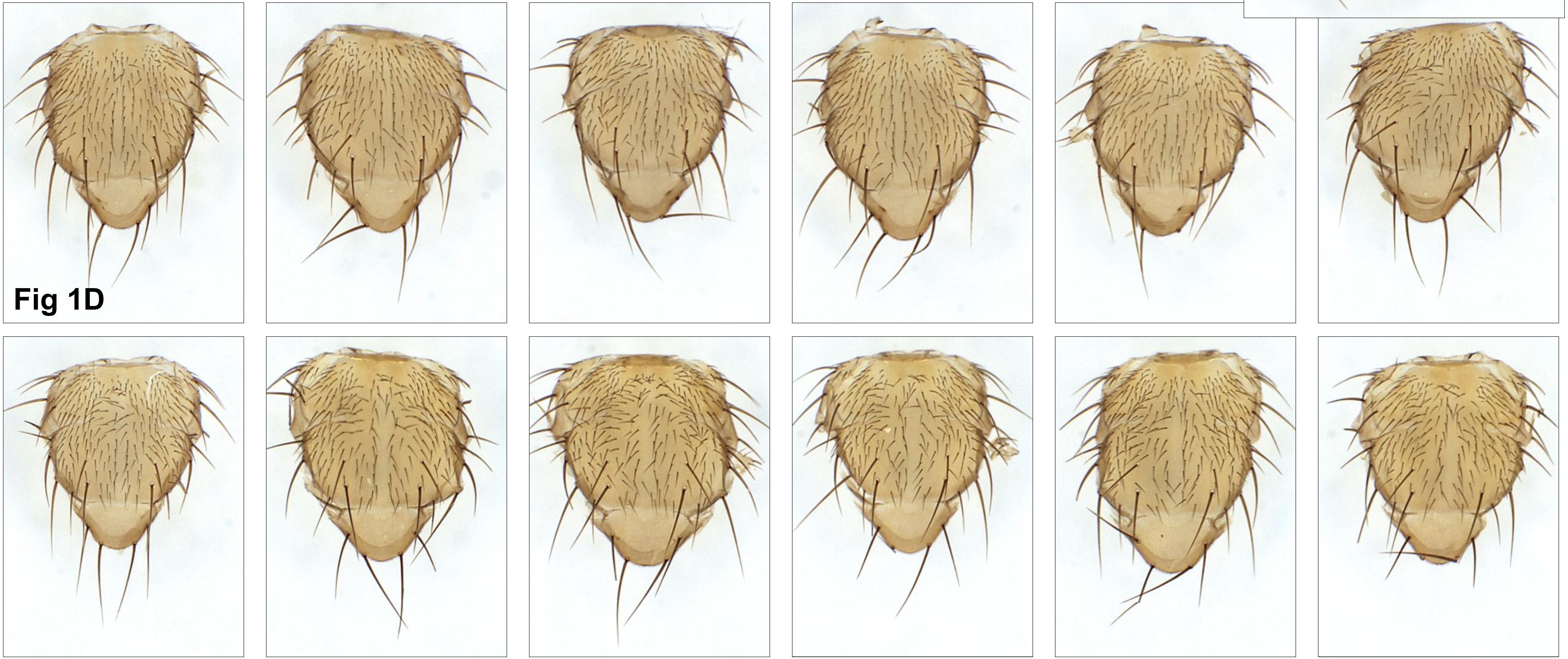

pnr>Ctrl-RNAi 29C (2of2)

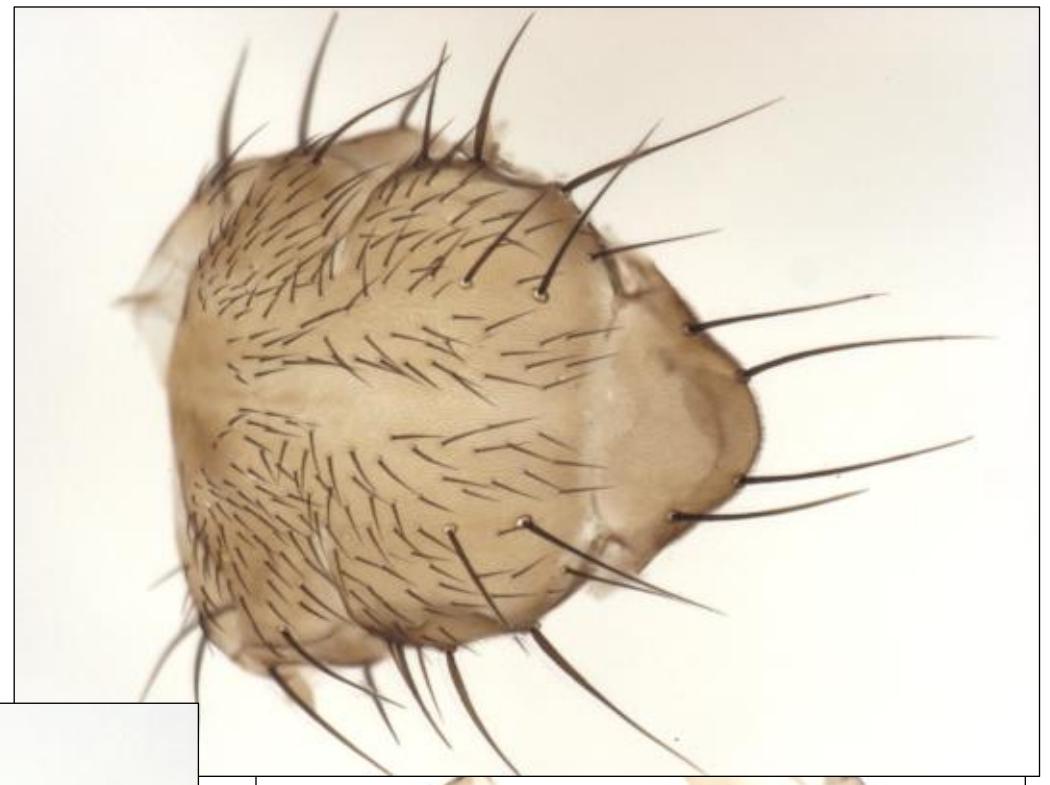

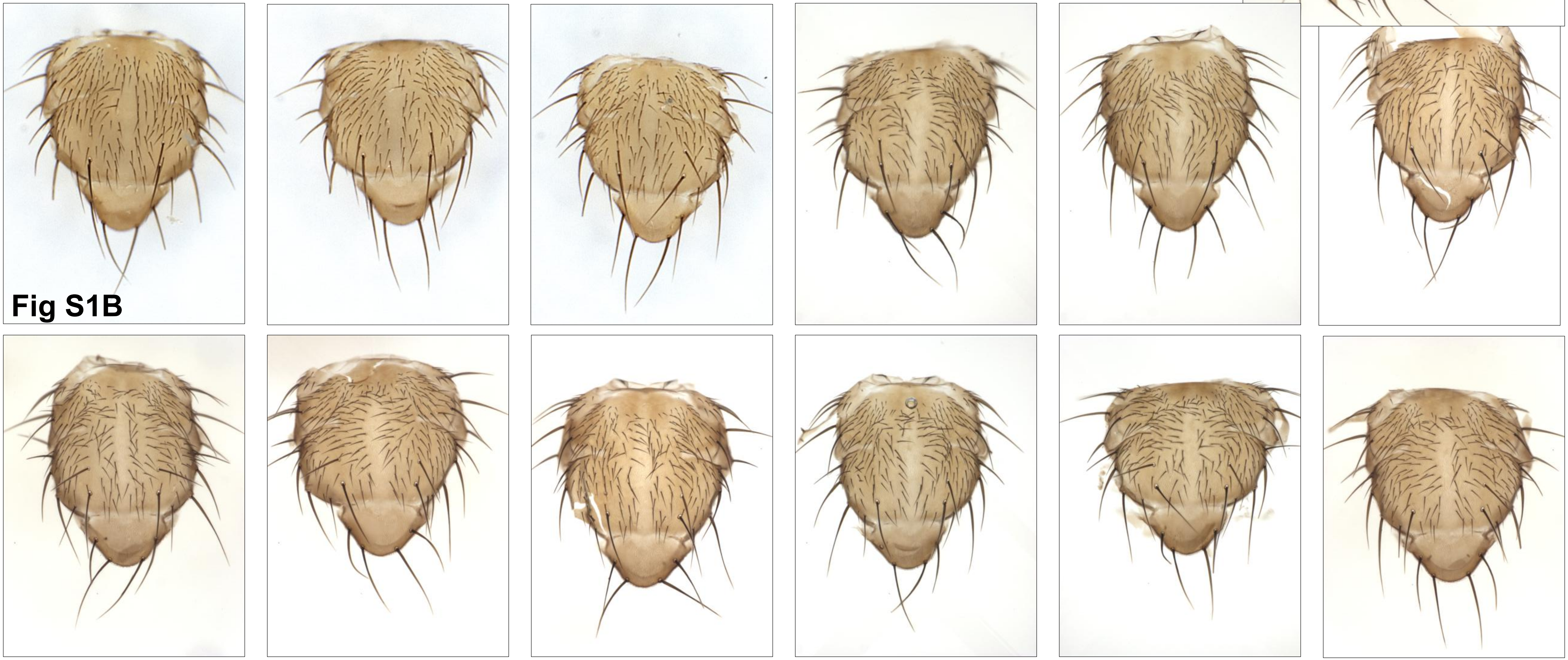

Fig S1B

pnr>Ctrl-RNAi 25C

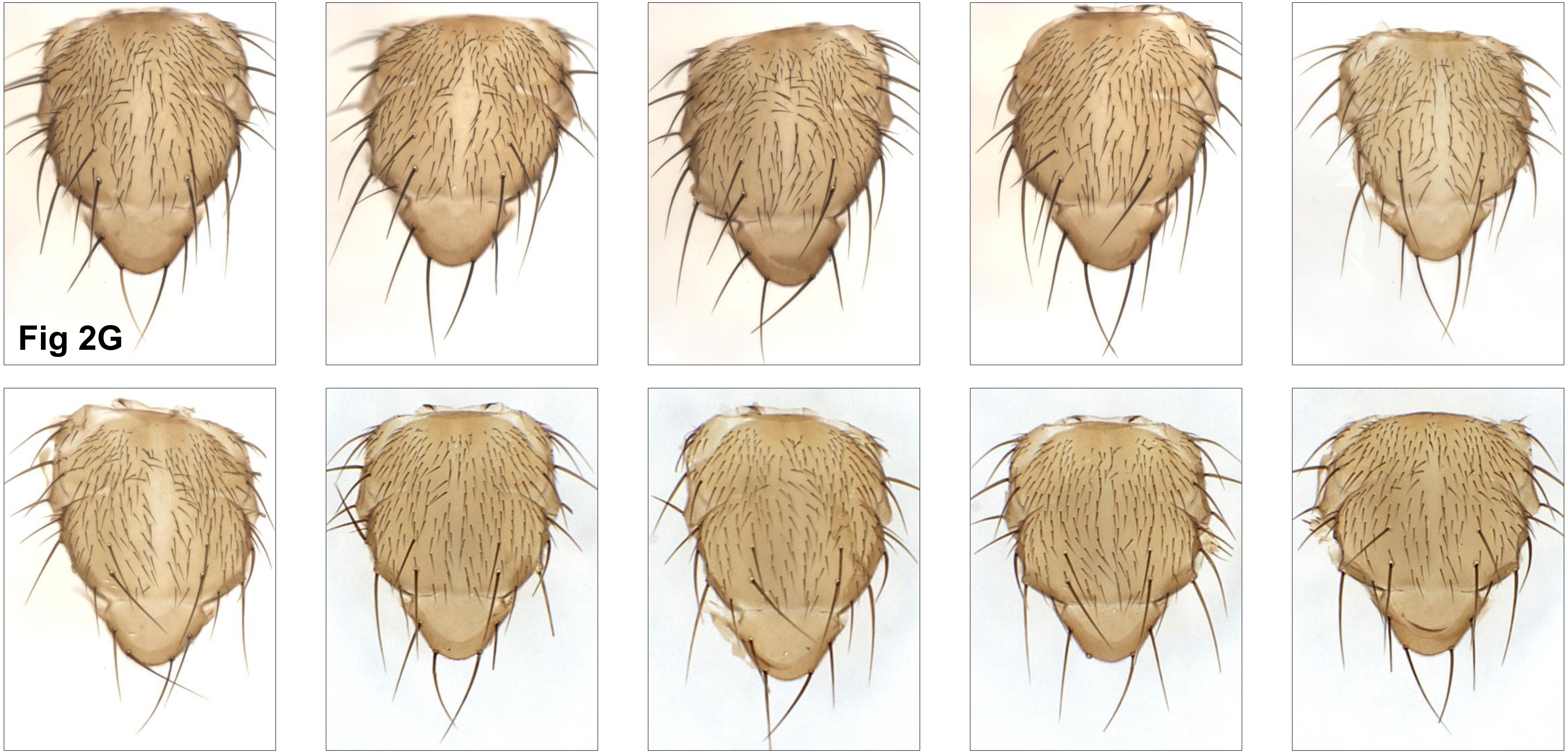

RNAi Controls with wts RNAi

pnr> Ctrl-RNAi, wts-RNAi-1 29C

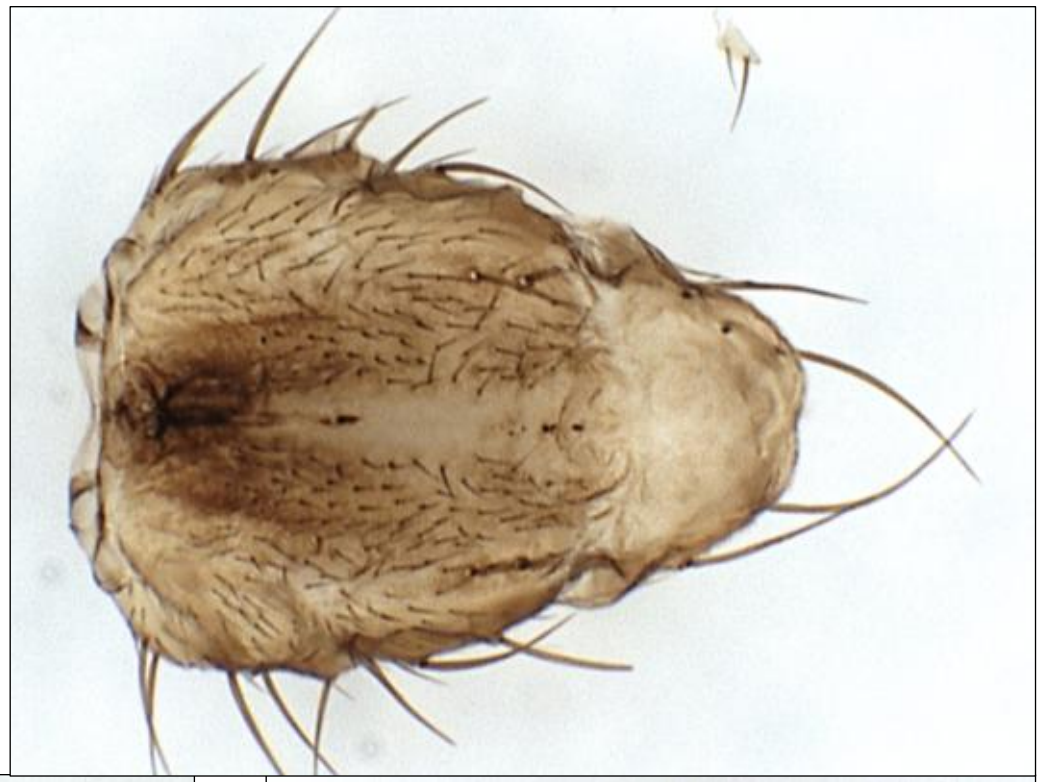

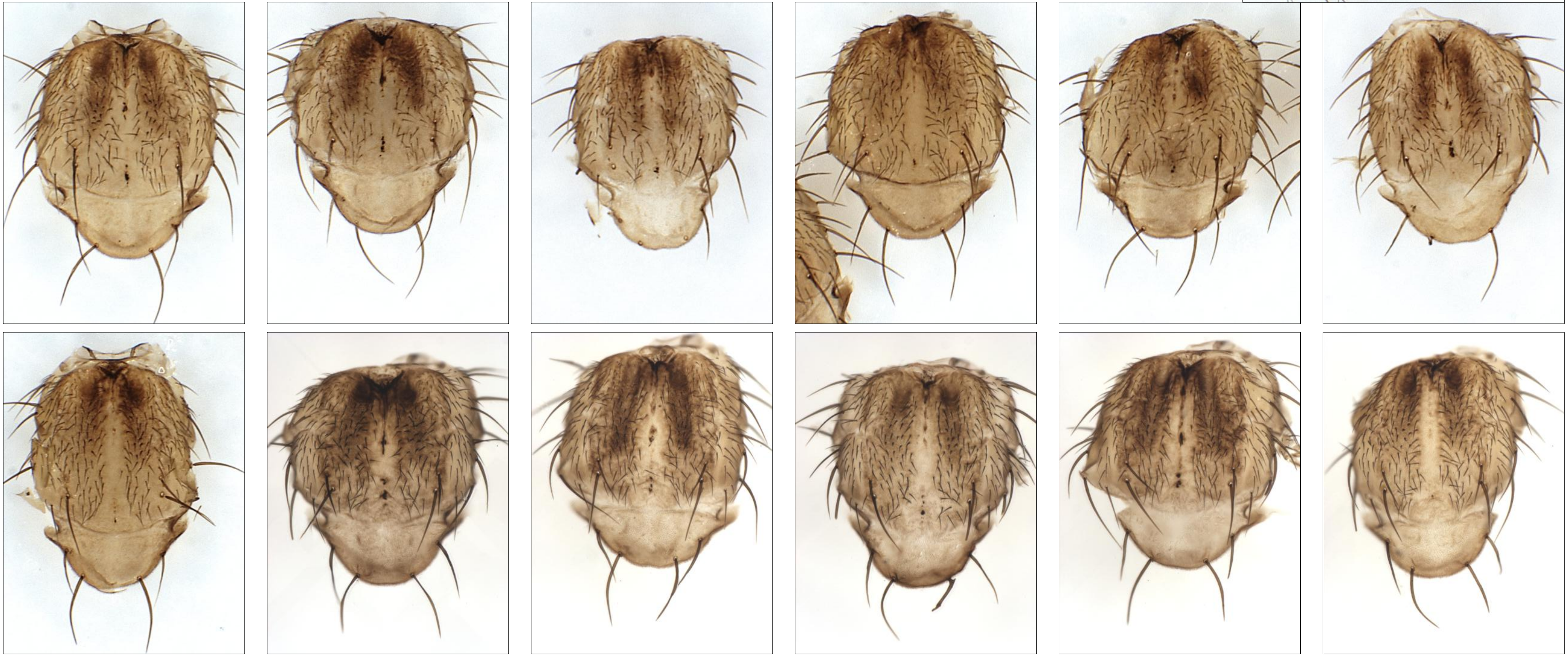

pnr>Ctrl-RNAi, wts-RNAi-1 25C

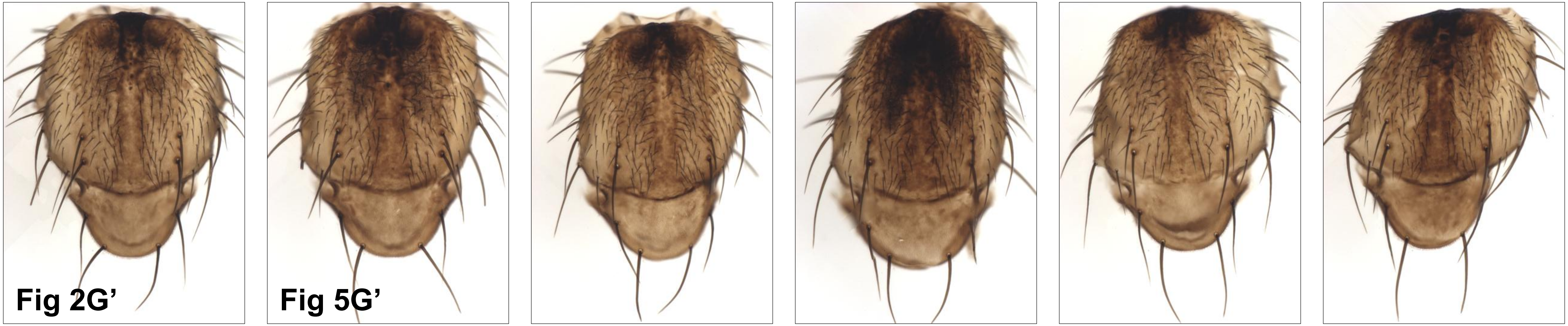

Overexpression Controls

pnr>Ctrl OE 29C

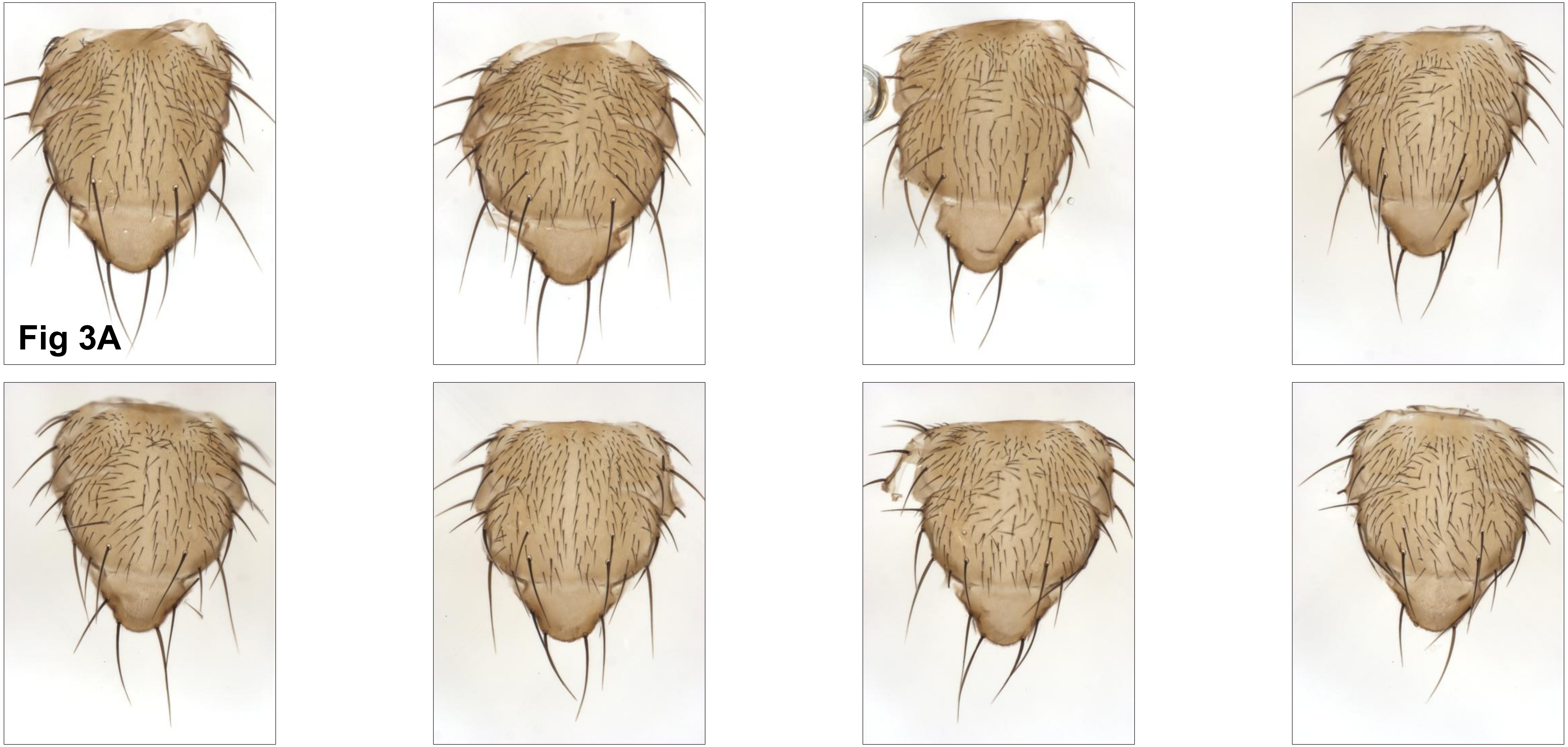

pnr>Ctrl OE 25C

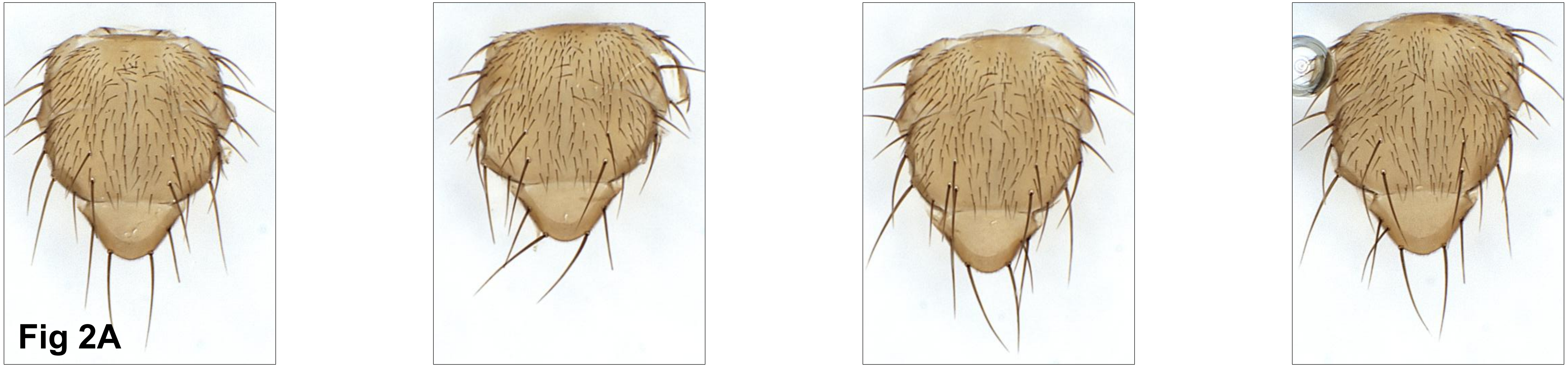

pnr>Ctrl OE RT

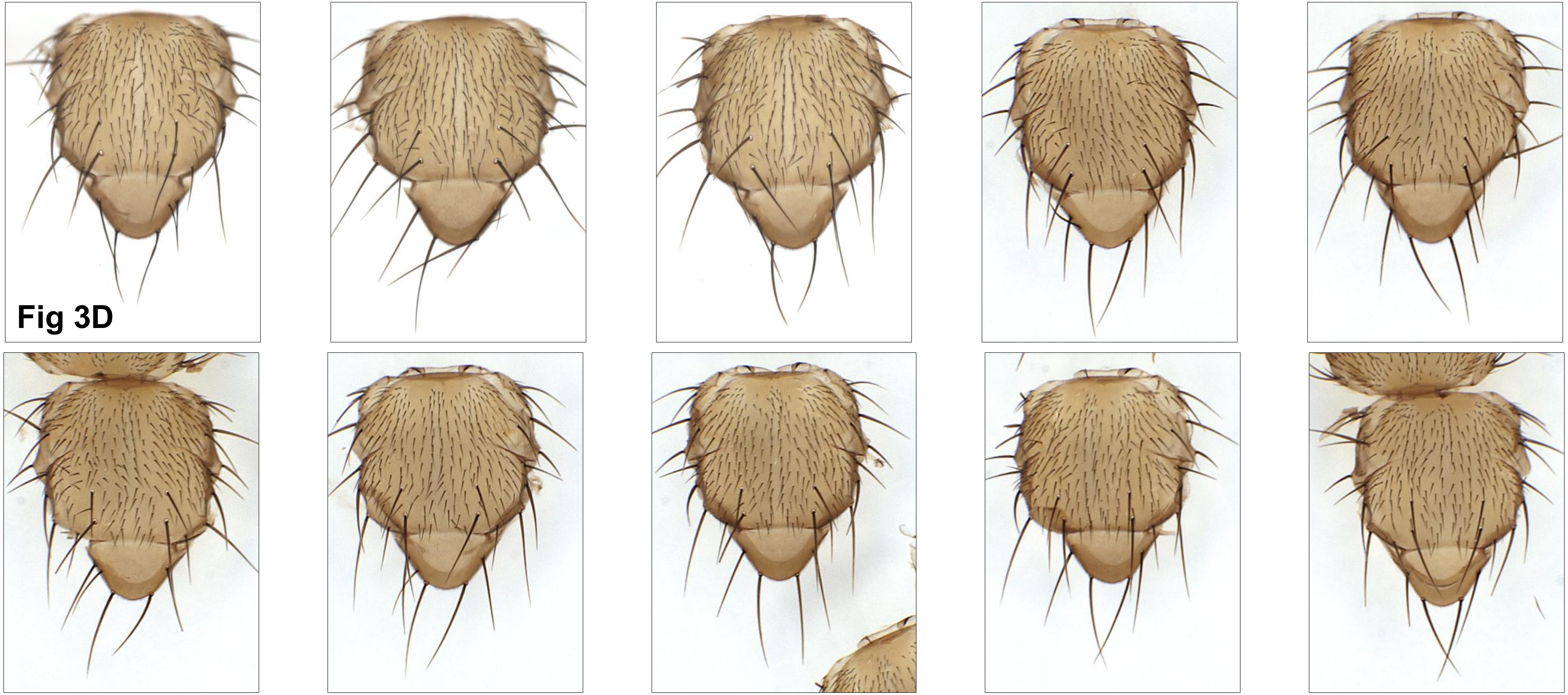

tubG80[ts];pnr>Ctrl OE 18>3rd>29C

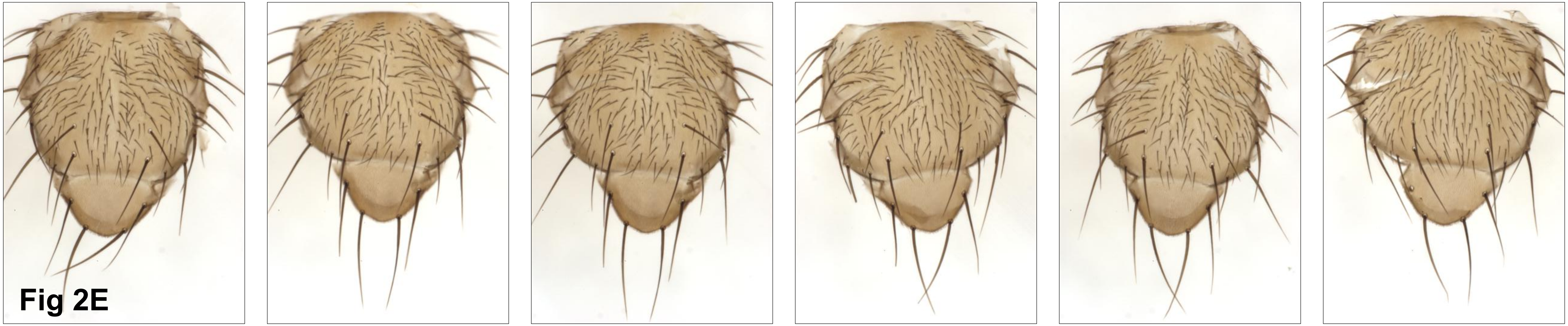

Overexpression Control with wts RNAi

pnr>Ctrl, wts-RNAi-1 25C

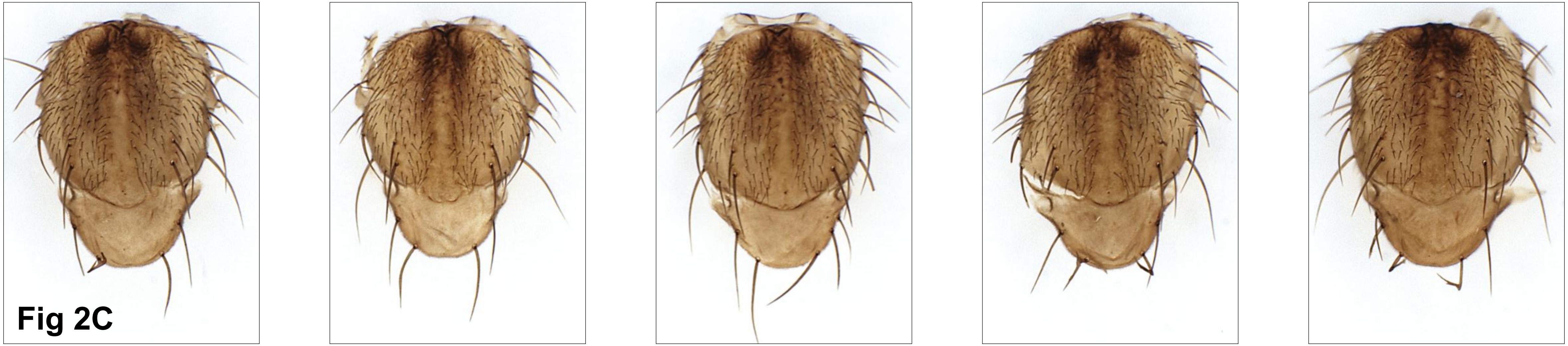

Fig 2C

pnr>Ctrl, wts-RNAi-1 RT

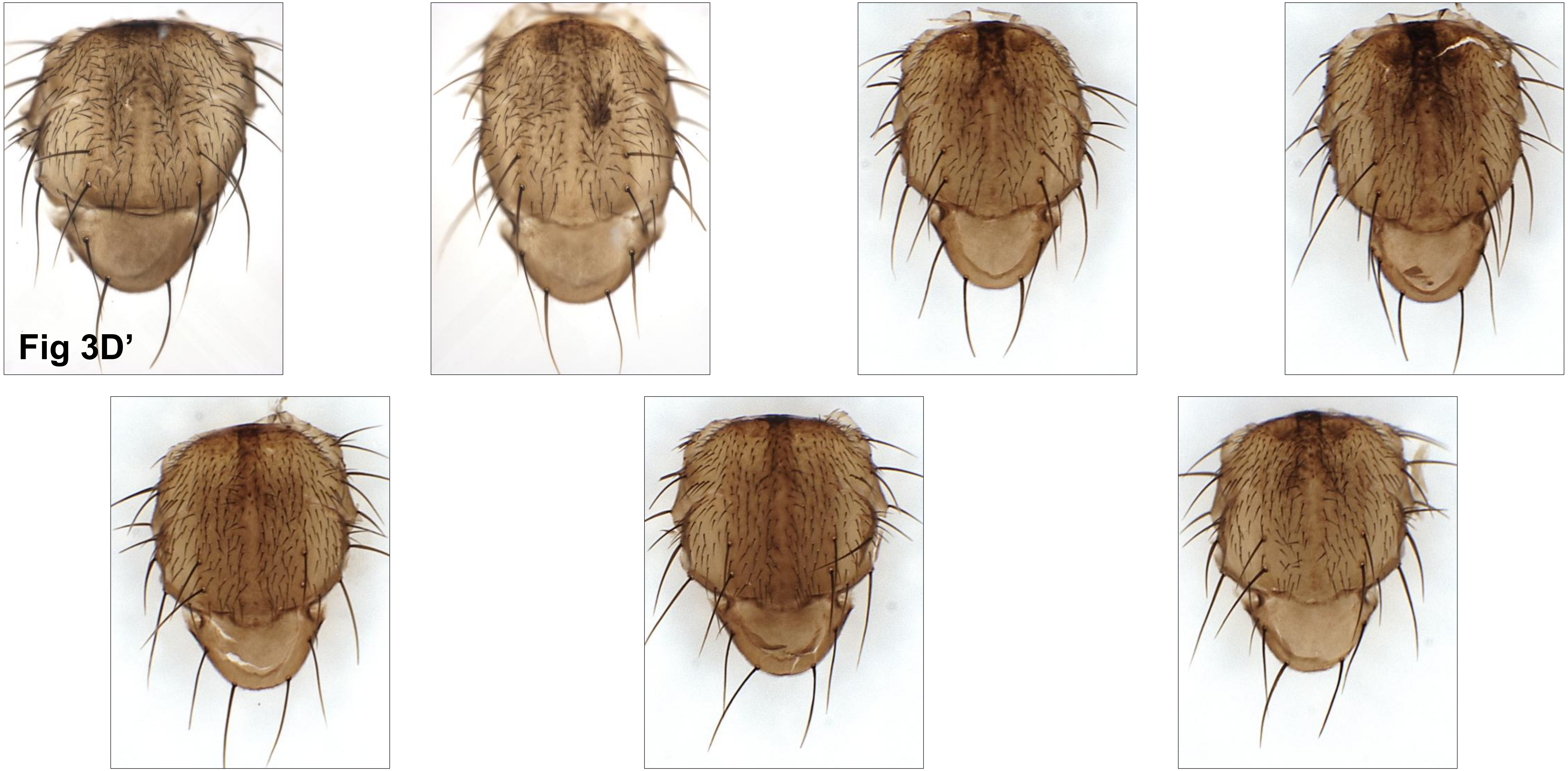

Overexpression Control with hpo RNAi

pnr>Ctrl, hpo-RNAi-1 25C

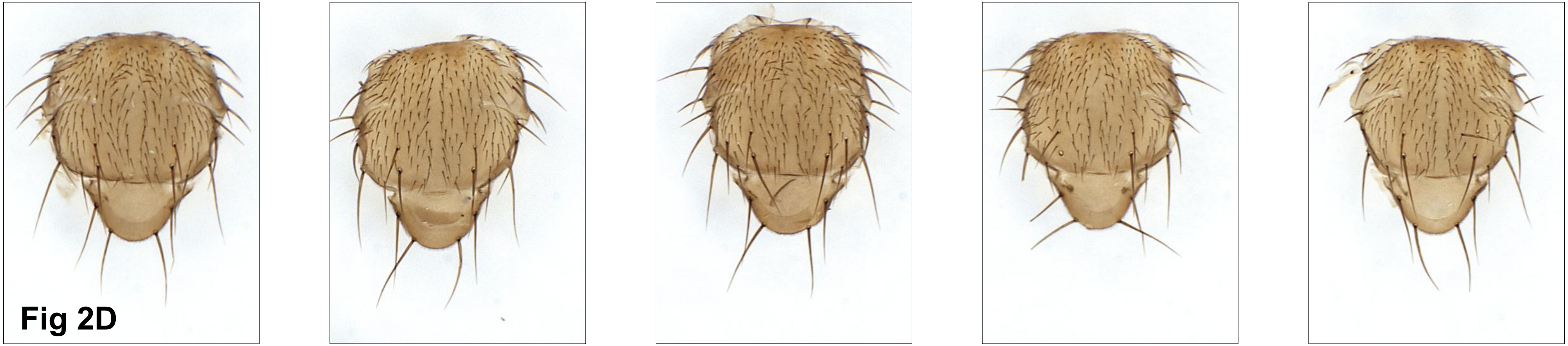

Fig 2D

Overexpression Control with mats RNAi

pnr>Ctrl OE, mats-RNAi-1 29C

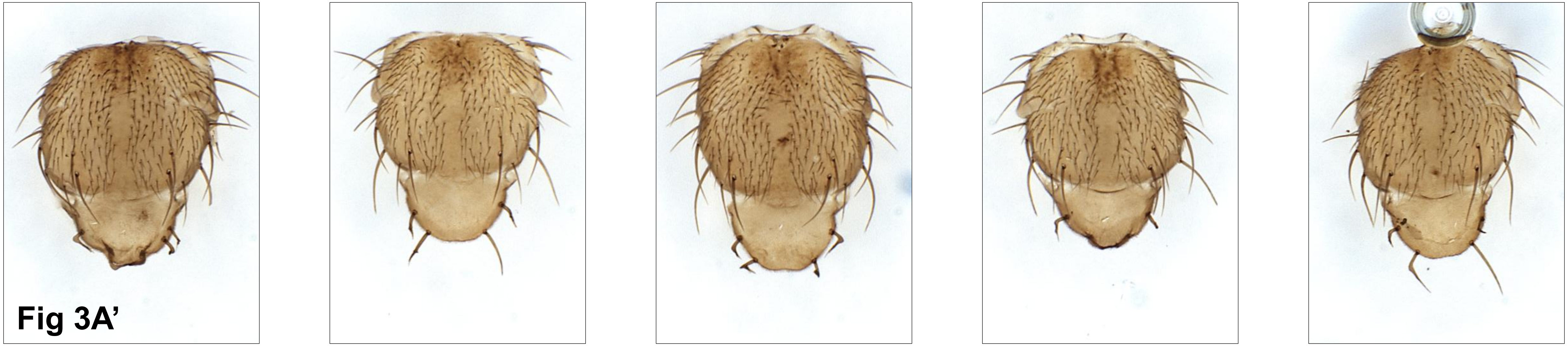

Overexpression Control with yki RNAi

pnr>Ctrl OE, yki-RNAi 25C

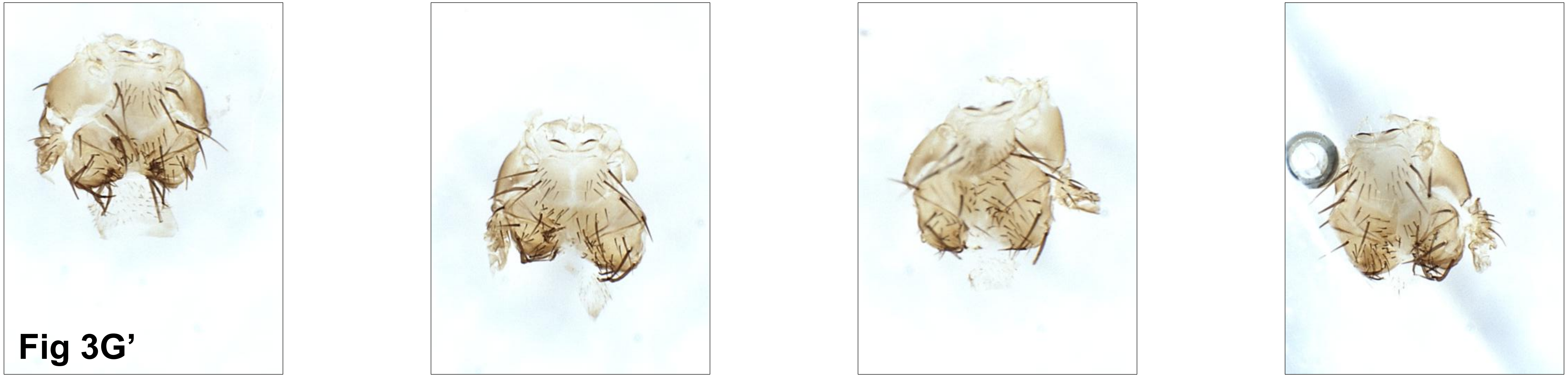

Fig 3G’

Notum Images

Figures 1, 2, 3

Figure 1

hpo gRNA ; pnr>Cas9 29C

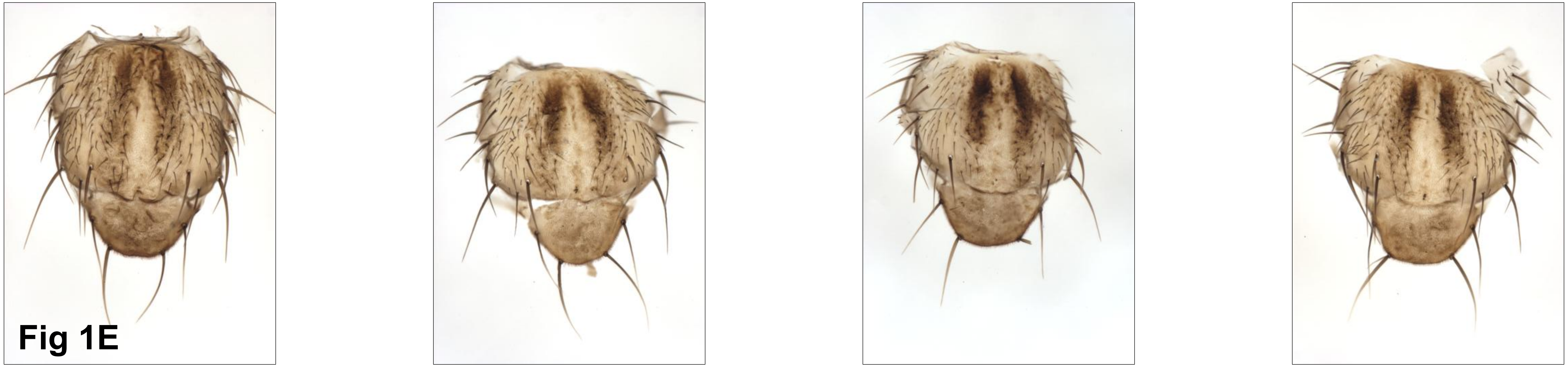

sav gRNA ; pnr>Cas9 29C

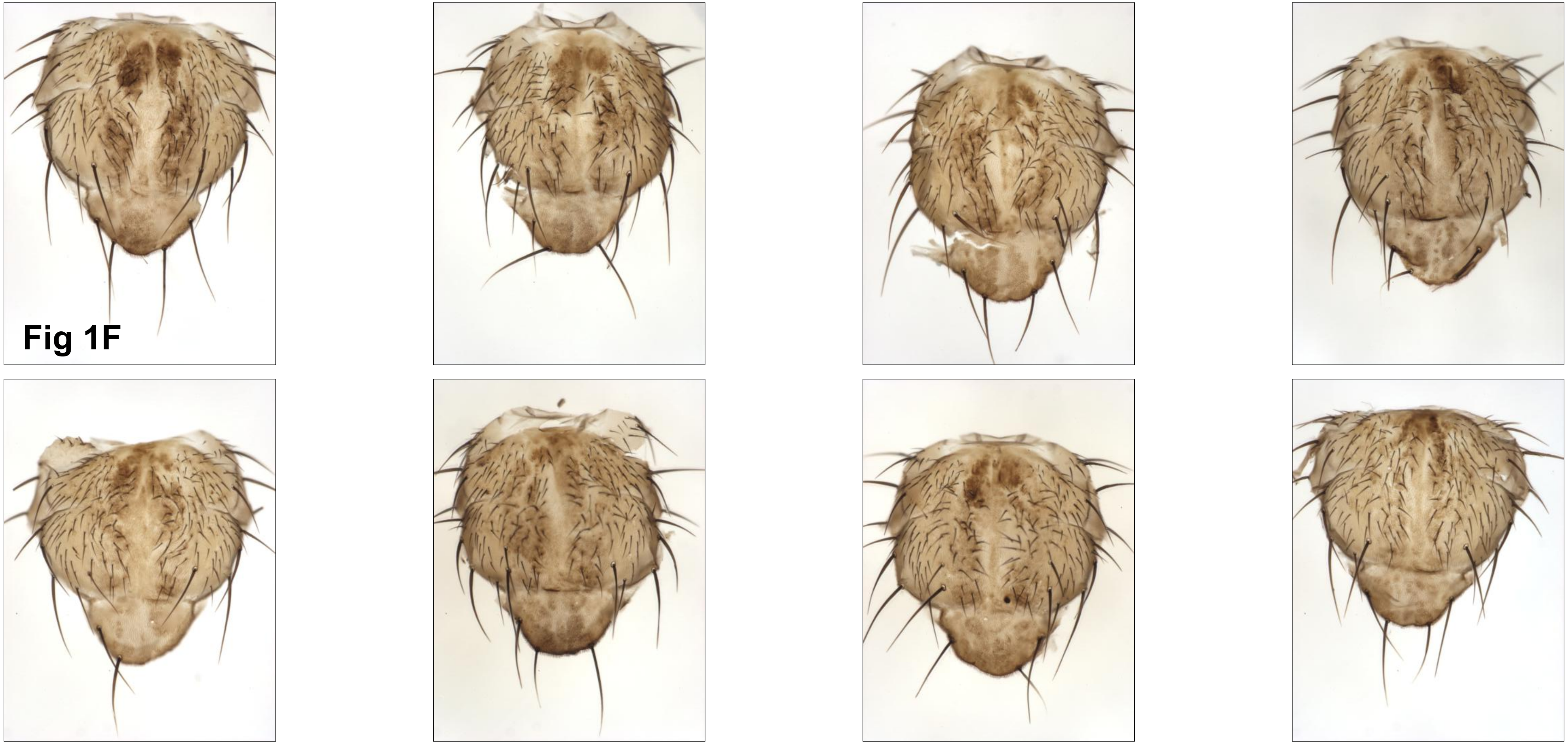

pnr>mats-RNAi-1 29C

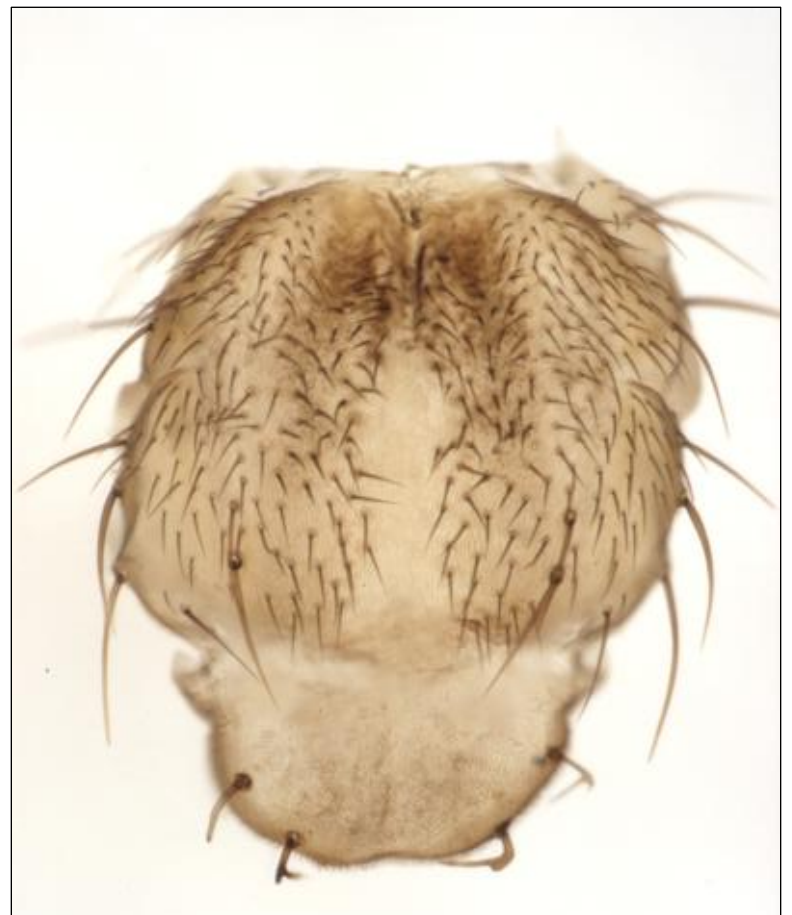
Fig 1G

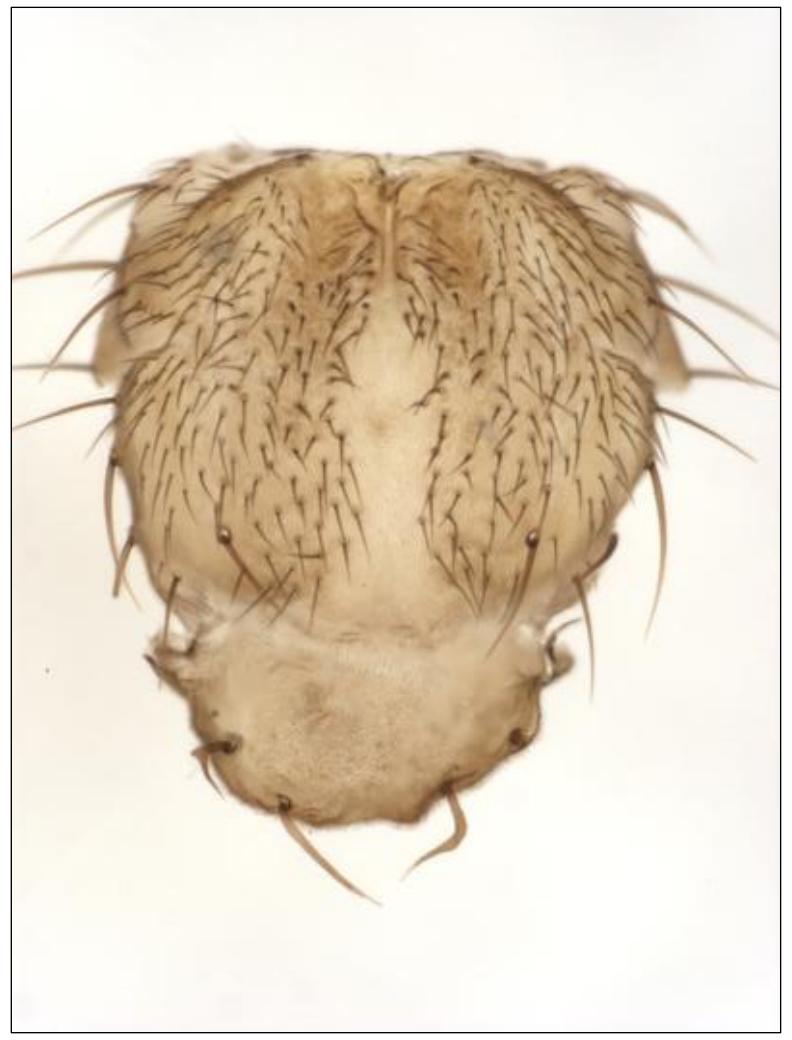

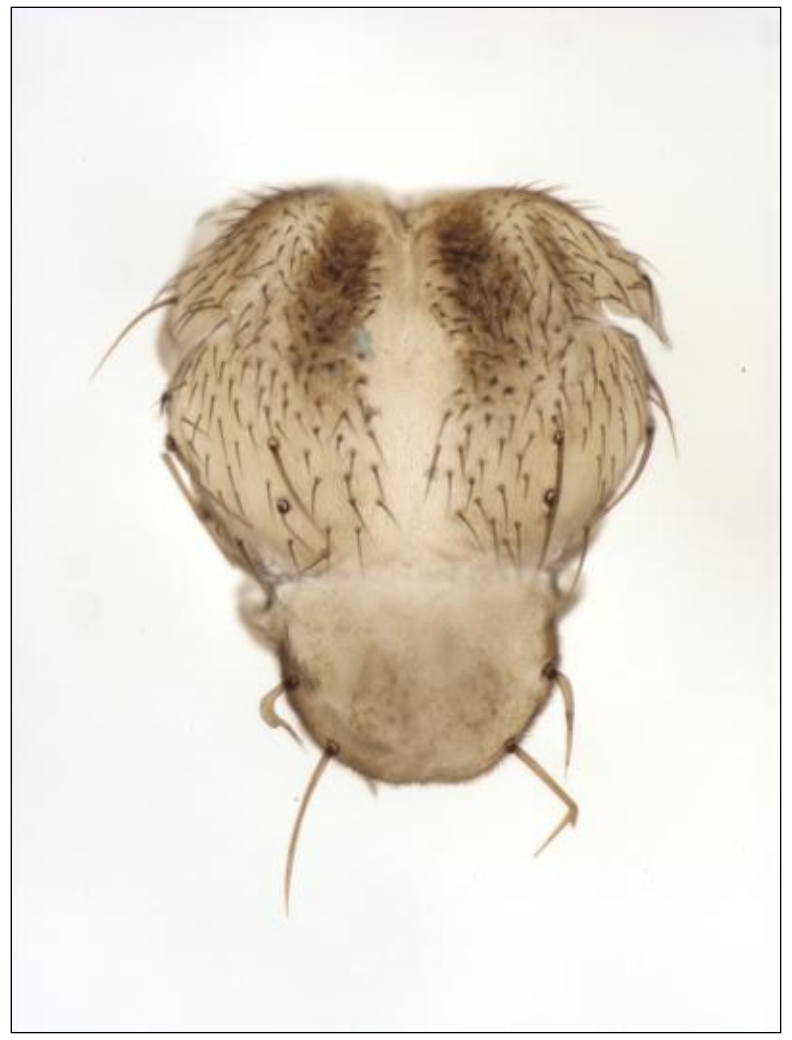

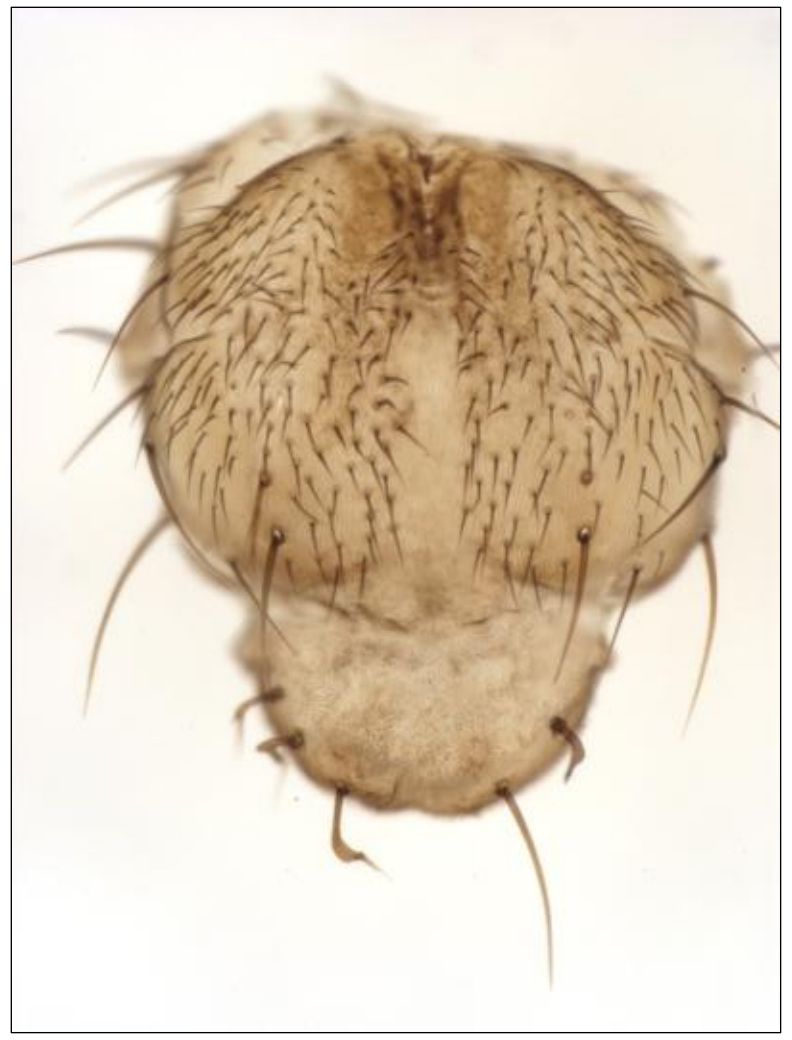

pnr>warts-RNAi-1 29C

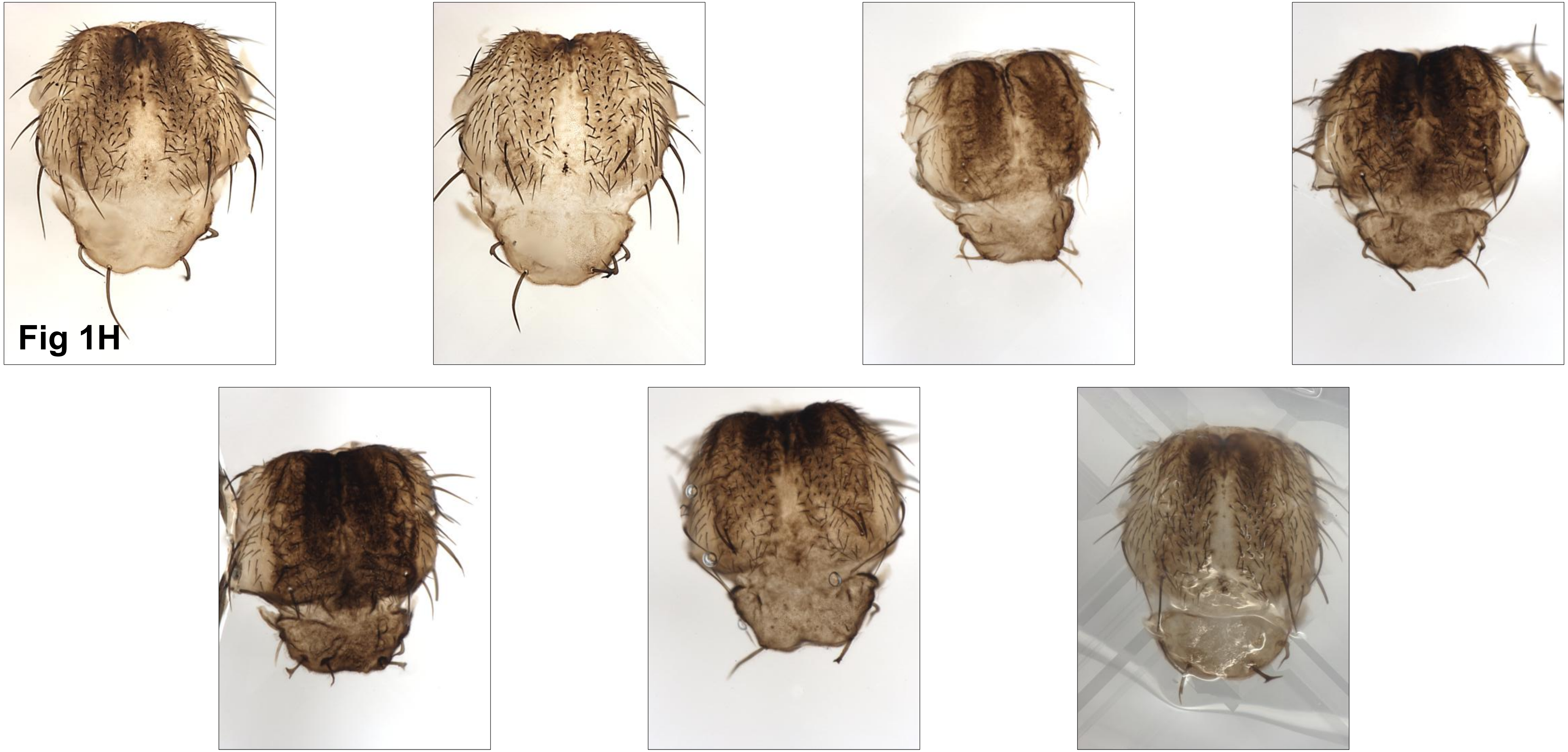

hpo gRNA ; pnr>Cas9, ple-RNAi 29C

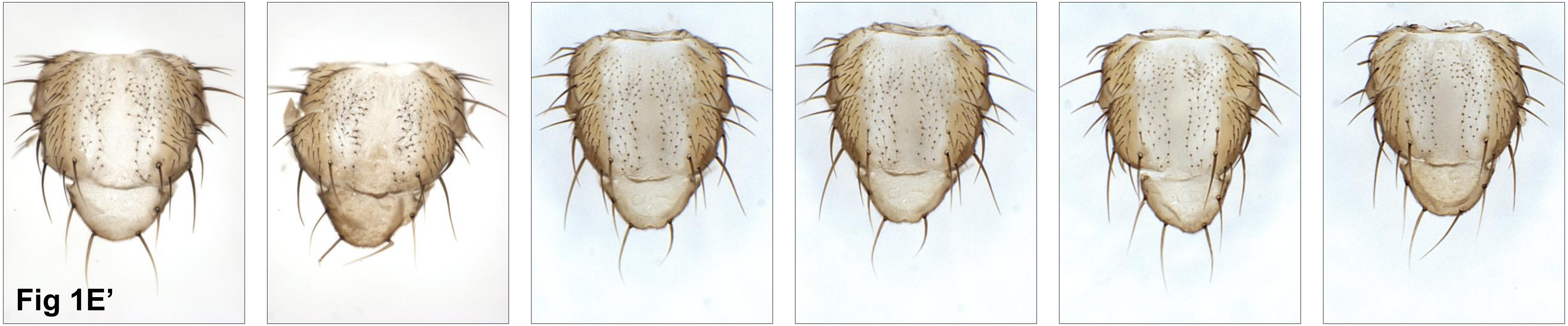

sav gRNA ; pnr>Cas9, ple-RNAi 29C

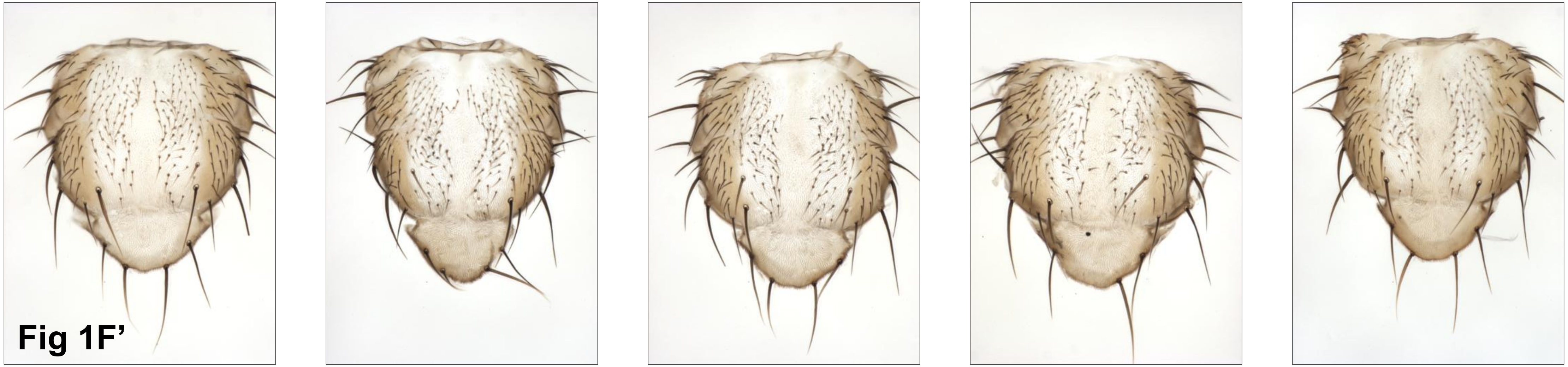

Fig 1F’

pnr>mats-RNAi-1, ple-RNAi 29C

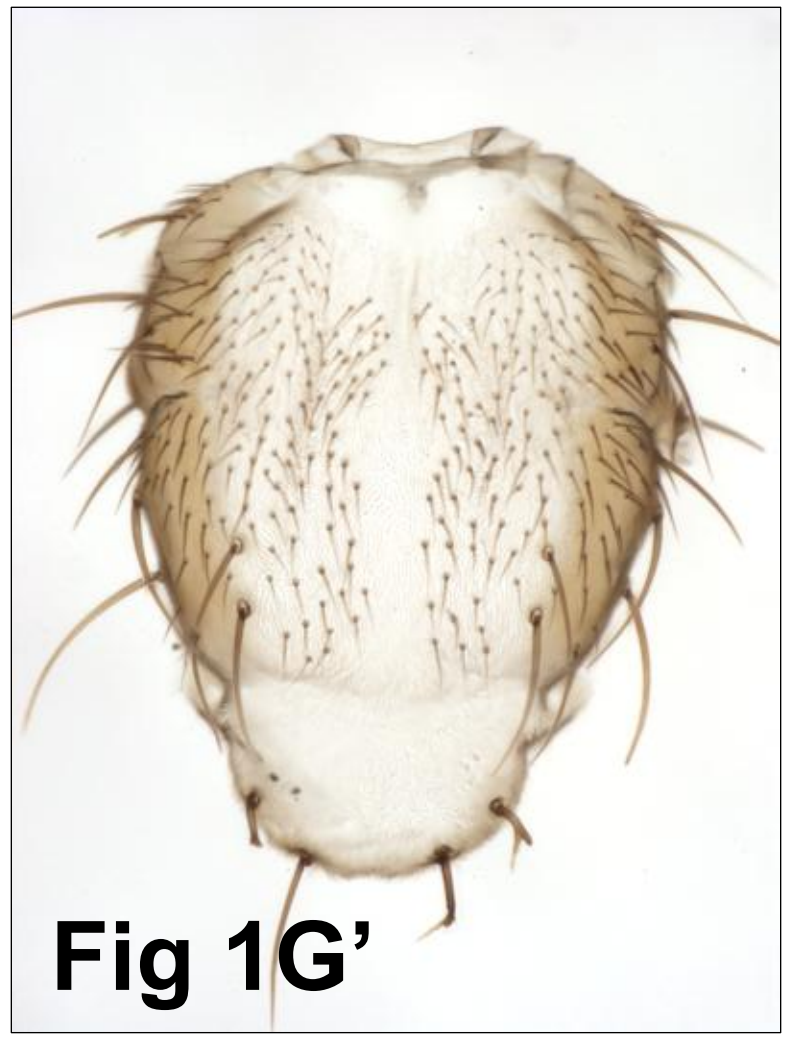

Fig 1G’

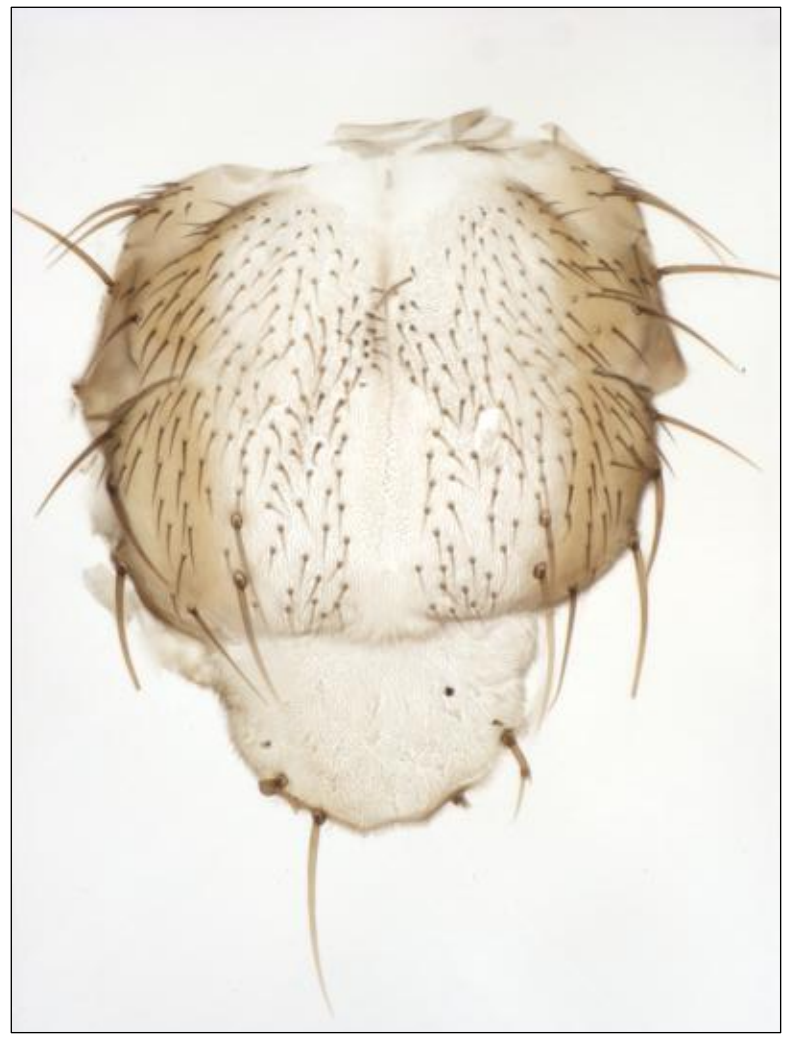

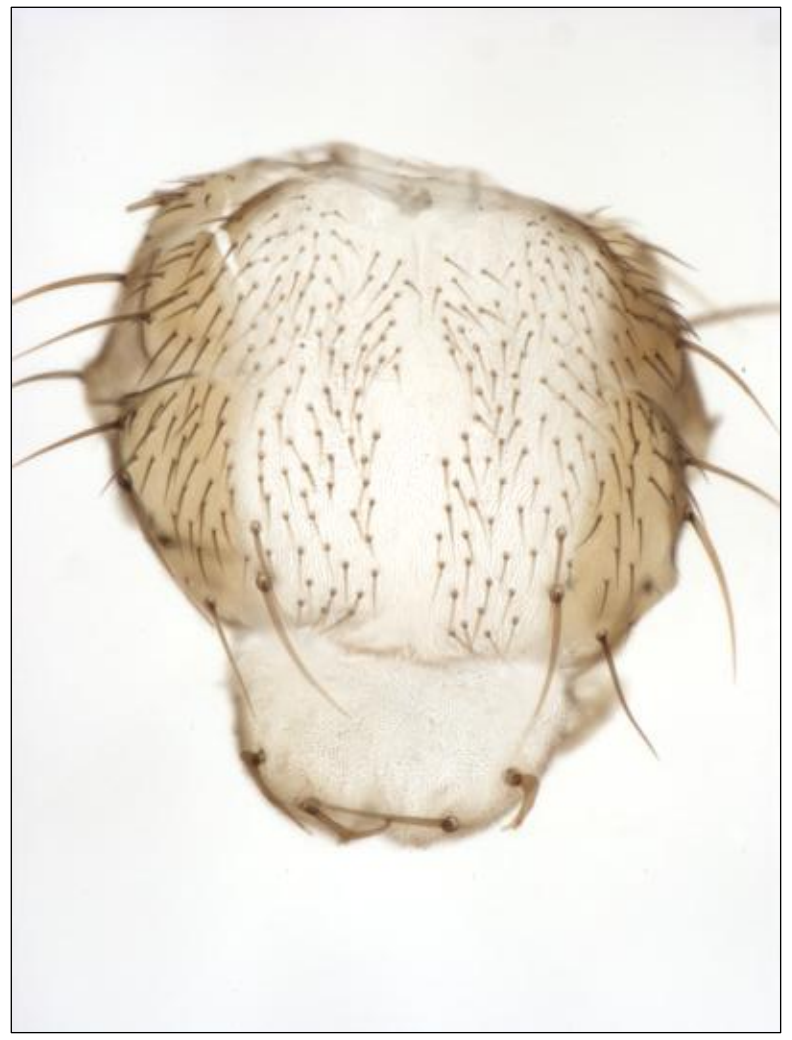

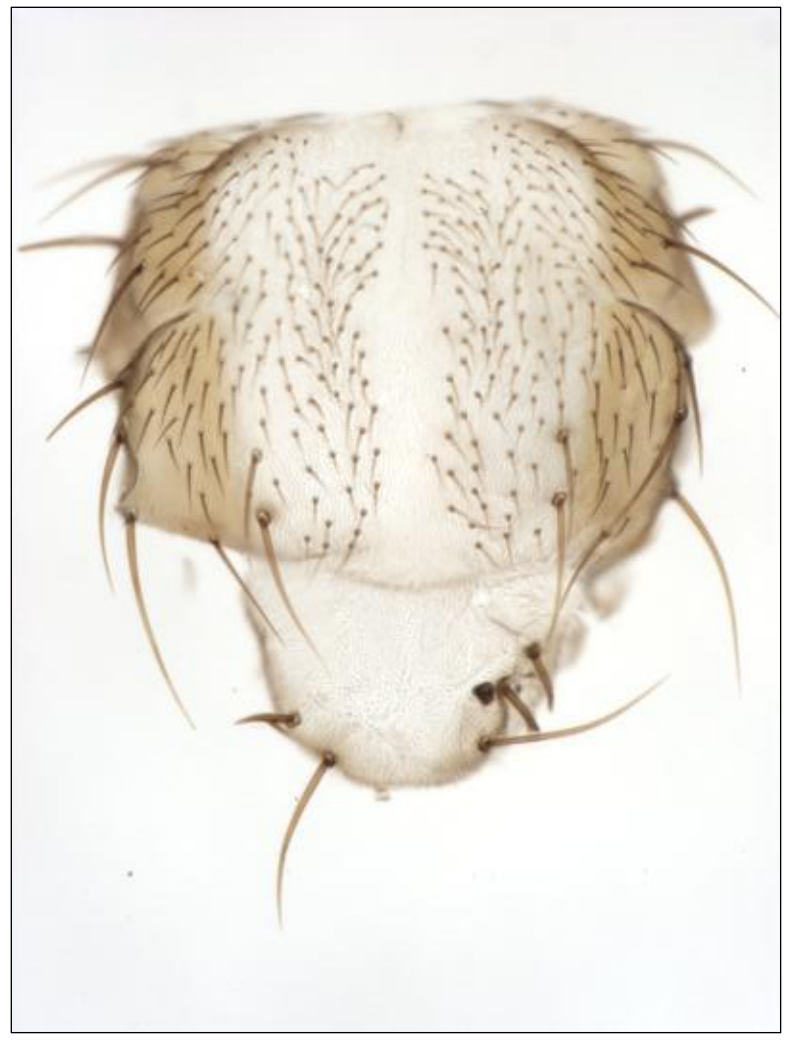

pnr>warts-RNAi-1, ple-RNAi 29C

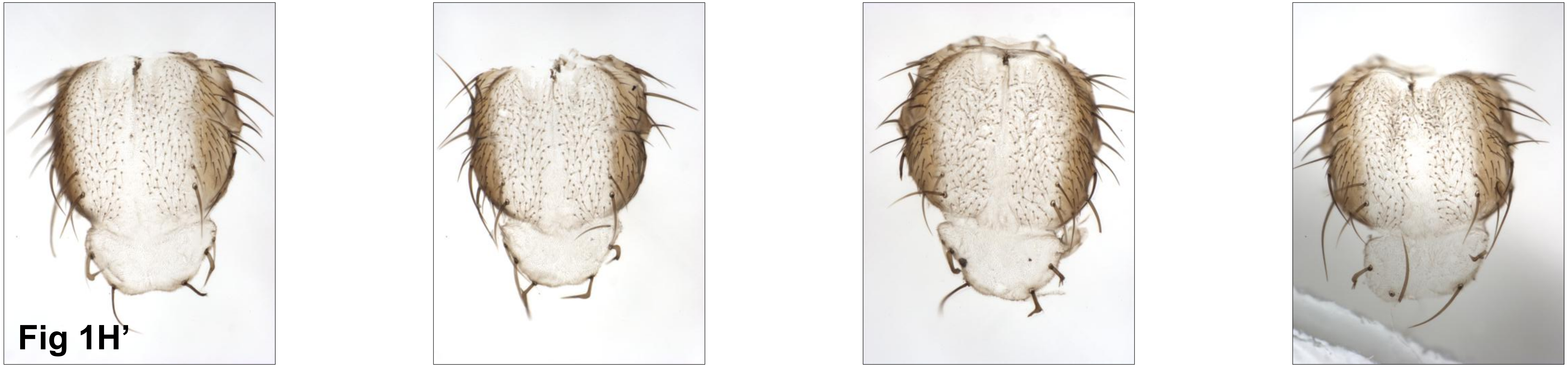

Figure 2

pnr>yki 25C

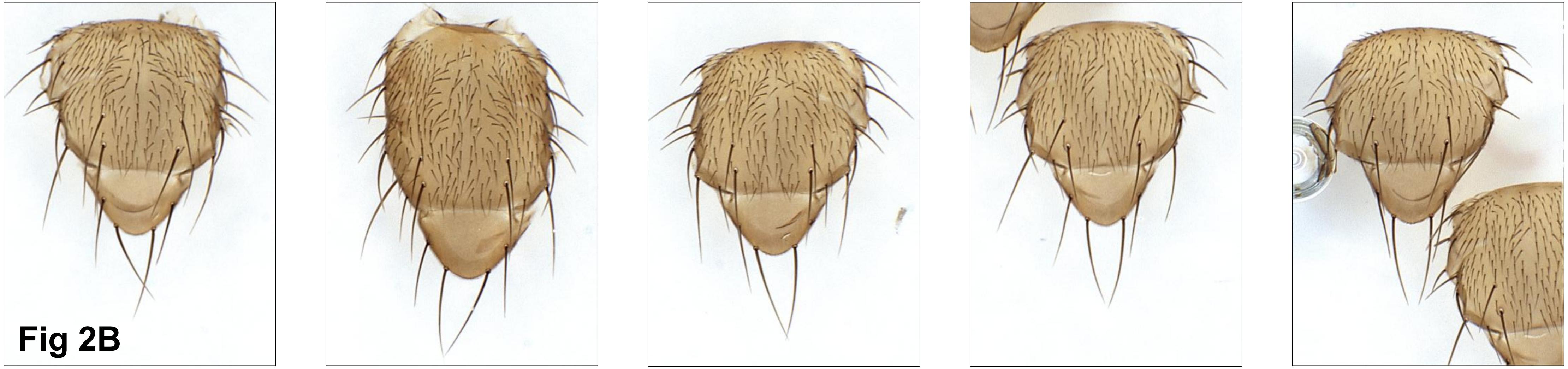

Fig 2B

pnr>yki, wts-RNAi-1 25C

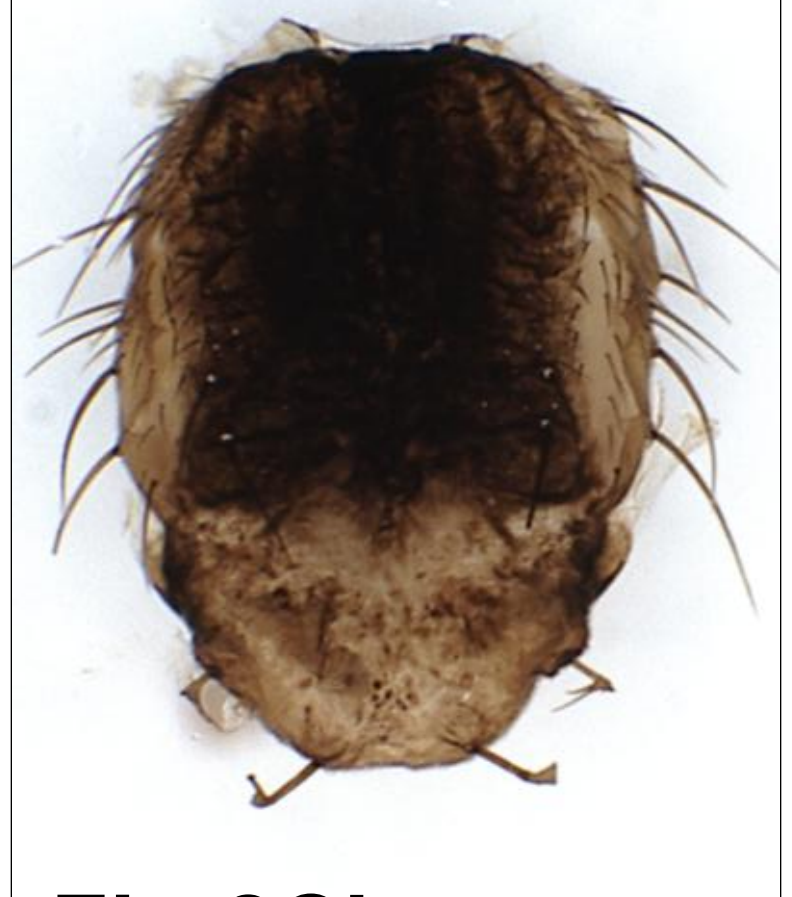

Fig 2C’

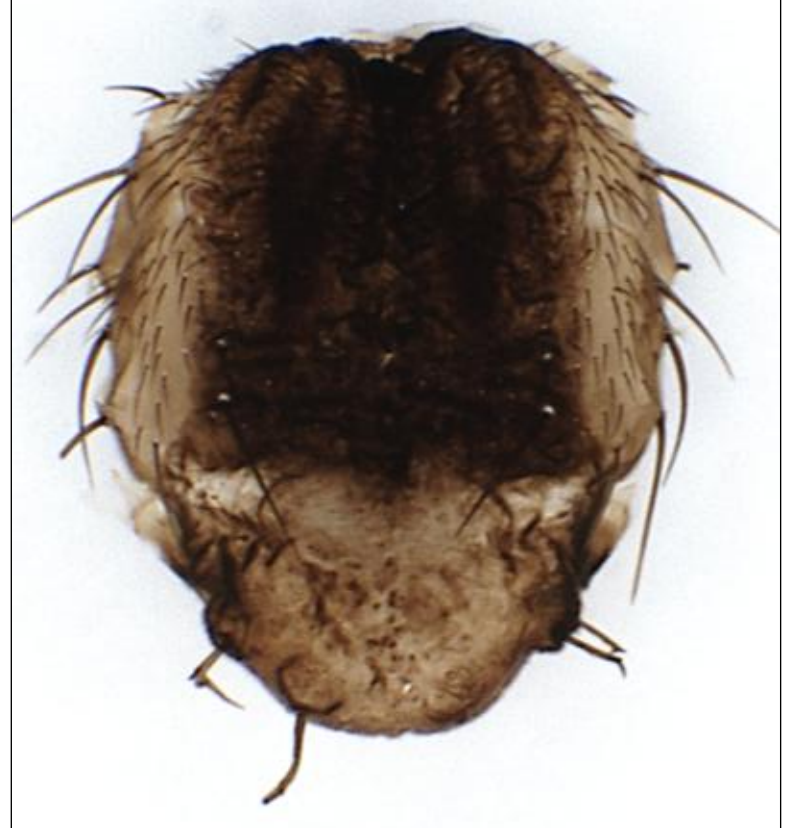

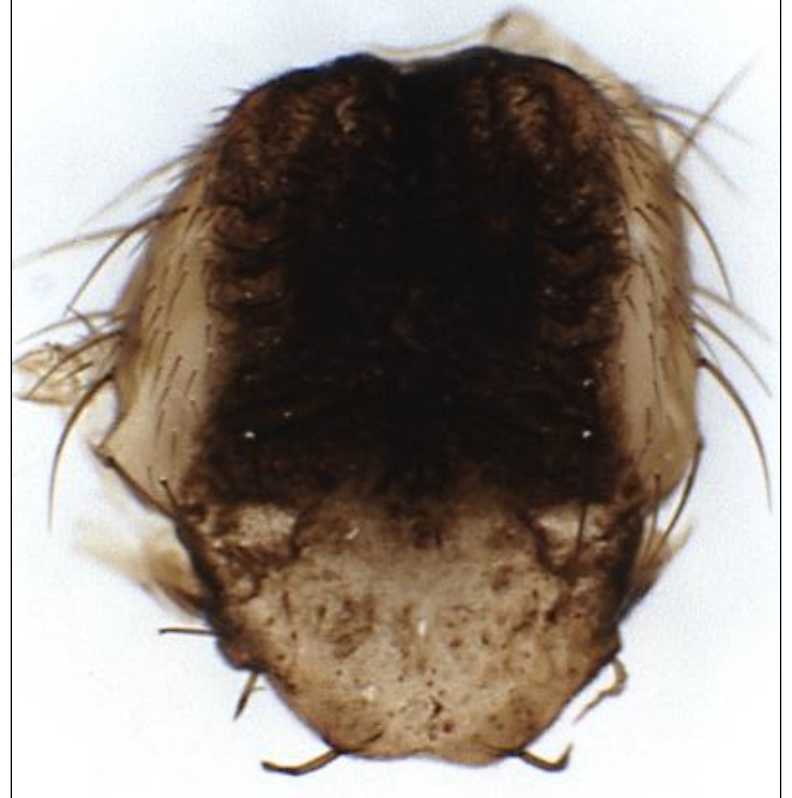

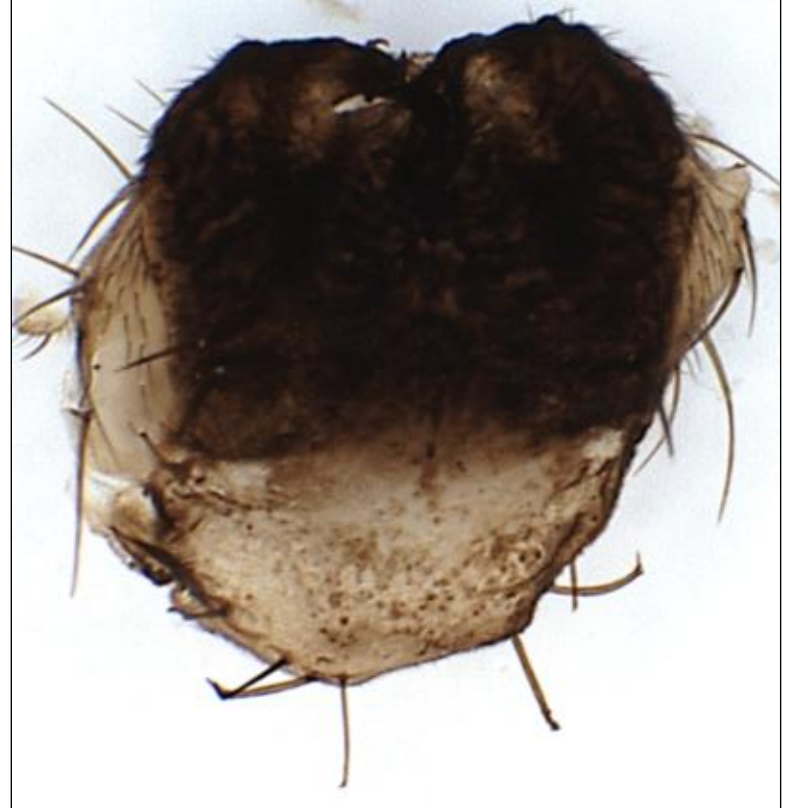

pnr>yki, hpo-RNAi-1 25C

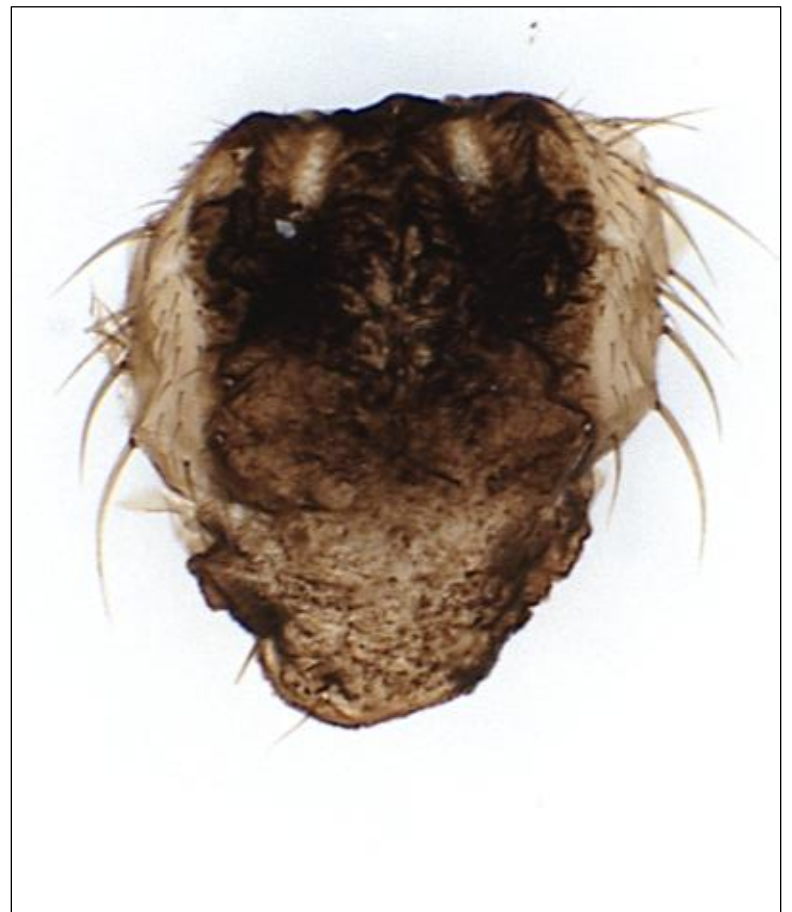

Fig 2D’

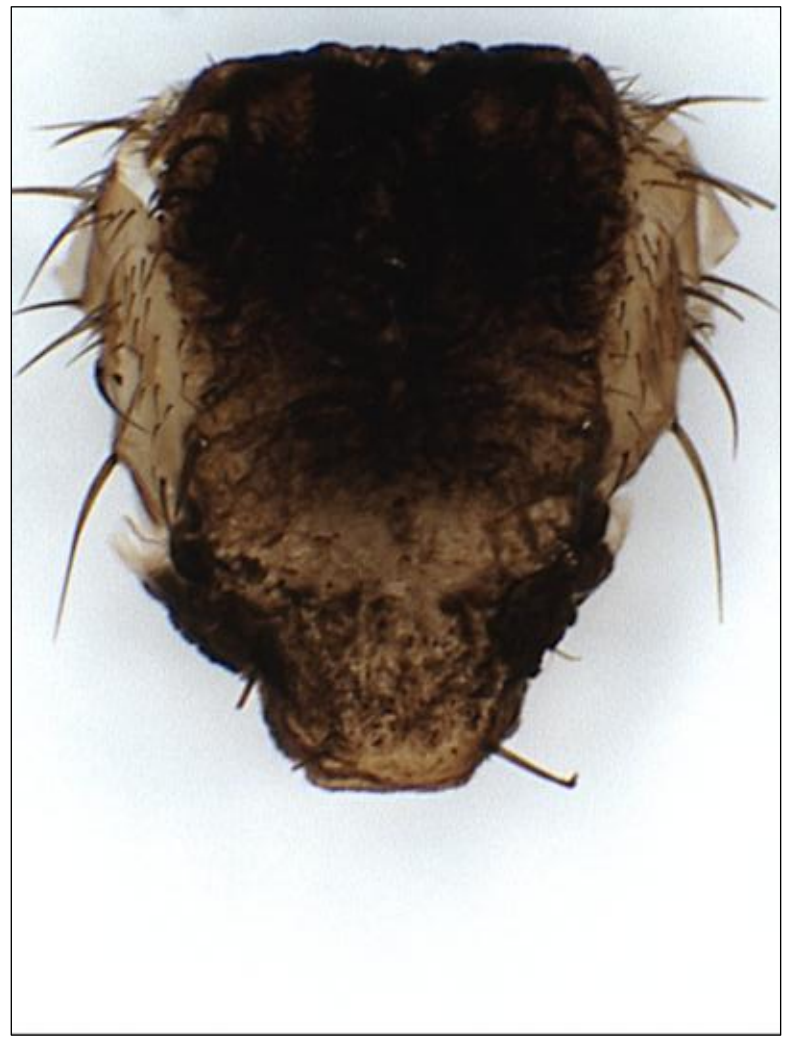

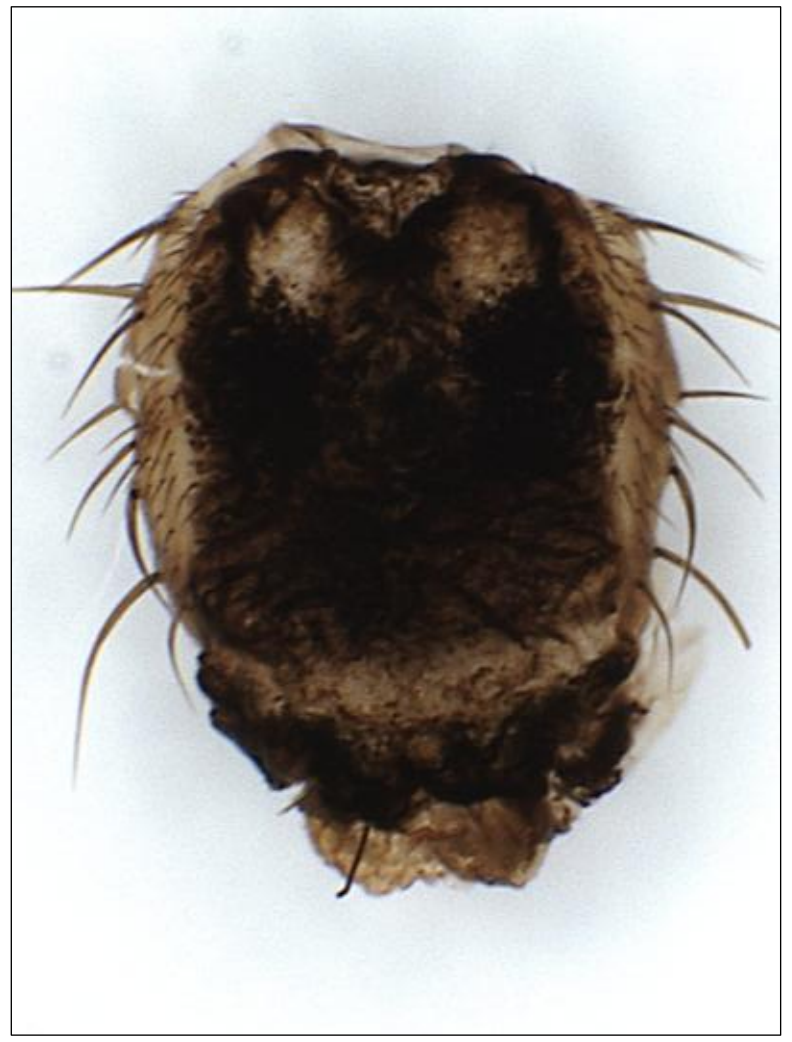

tubG80[ts] ; pnr>yki.S186A 18>3rd>29C

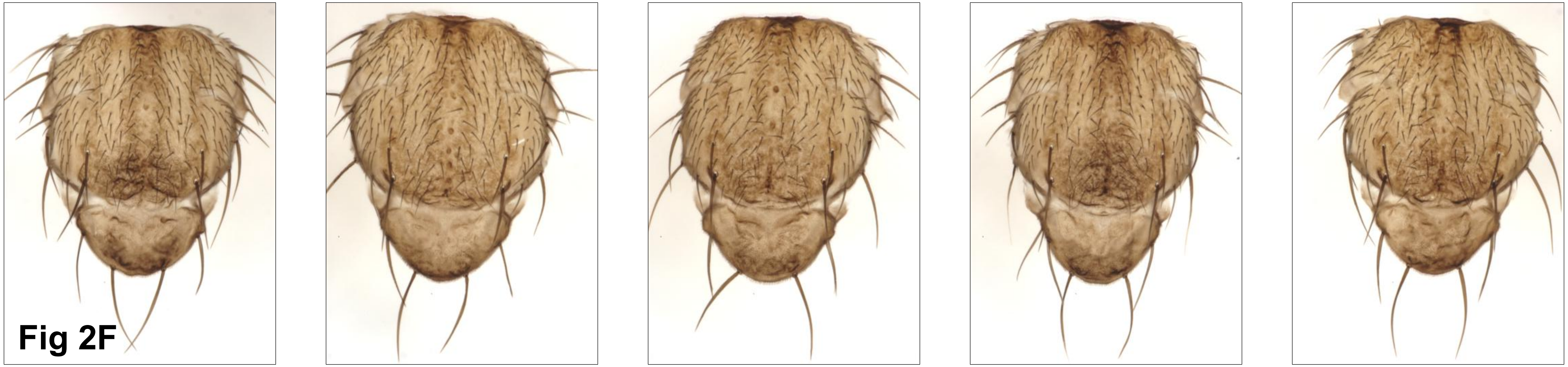

tubG80[ts] ; pnr>yki.S168A, ple-RNAi 18>3rd>29C

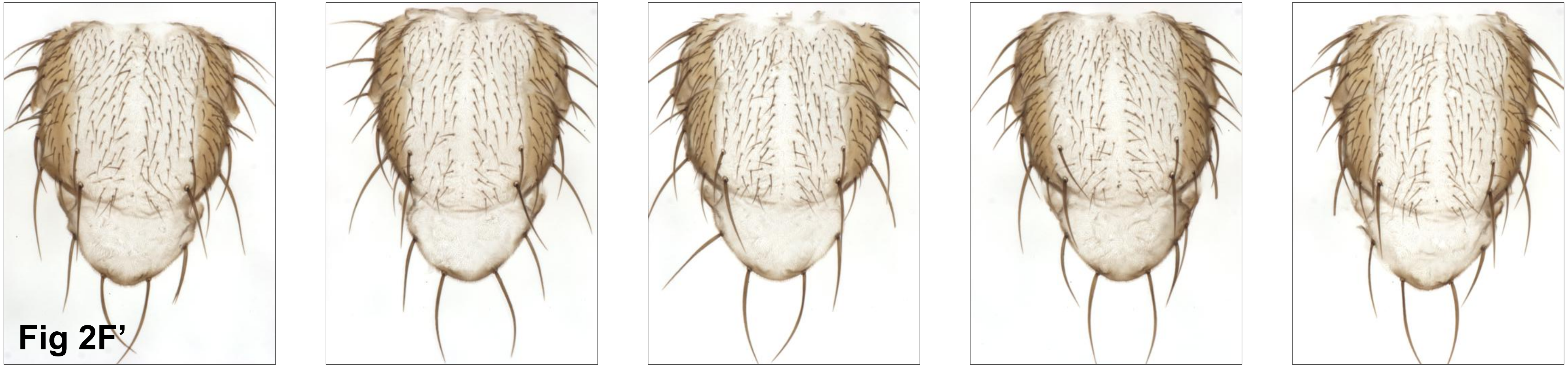

pnr>yki-RNAi-1 25C

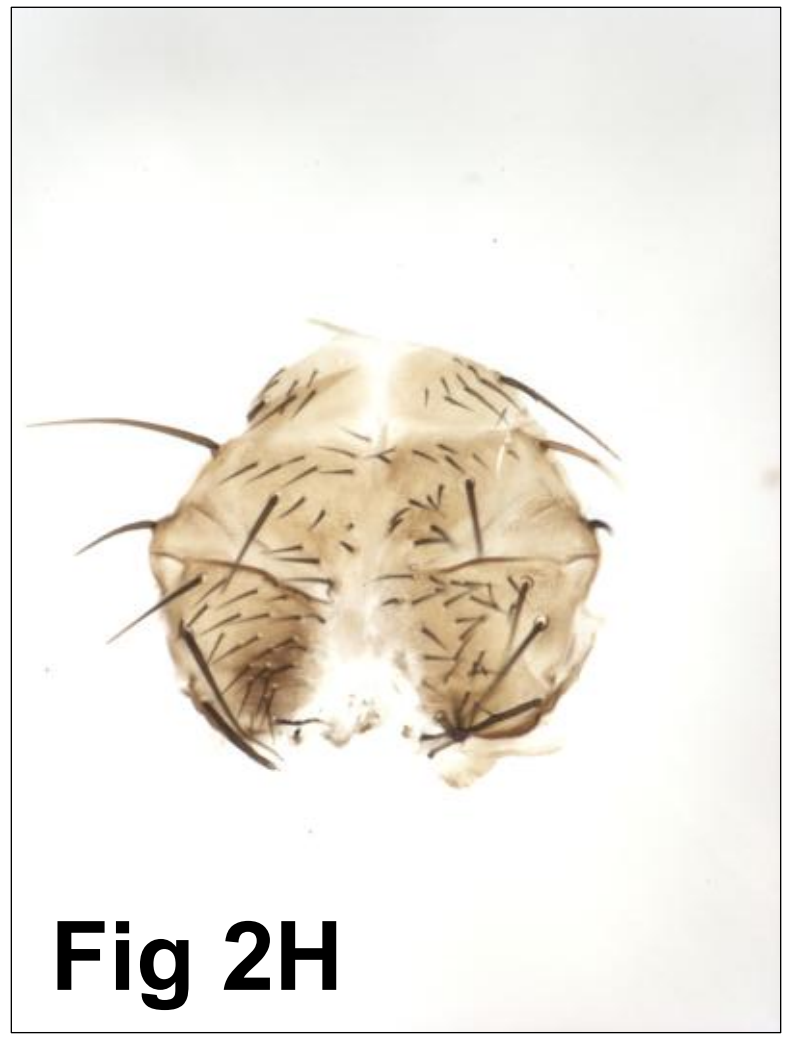

Fig 2H

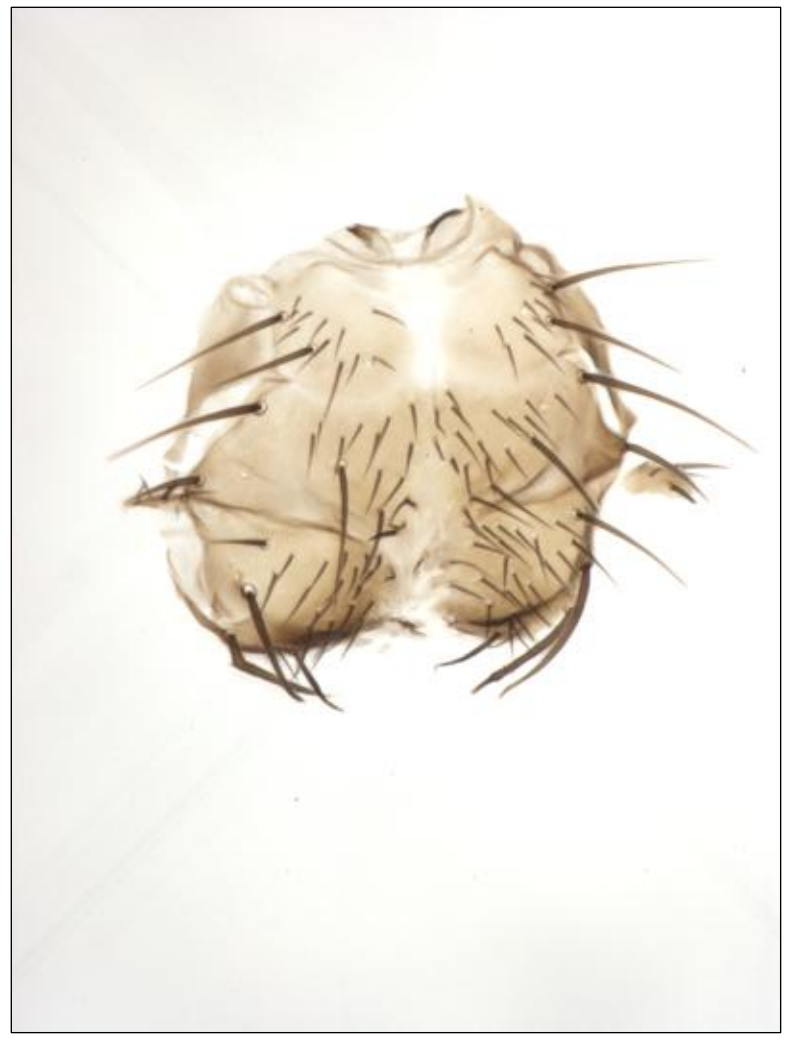

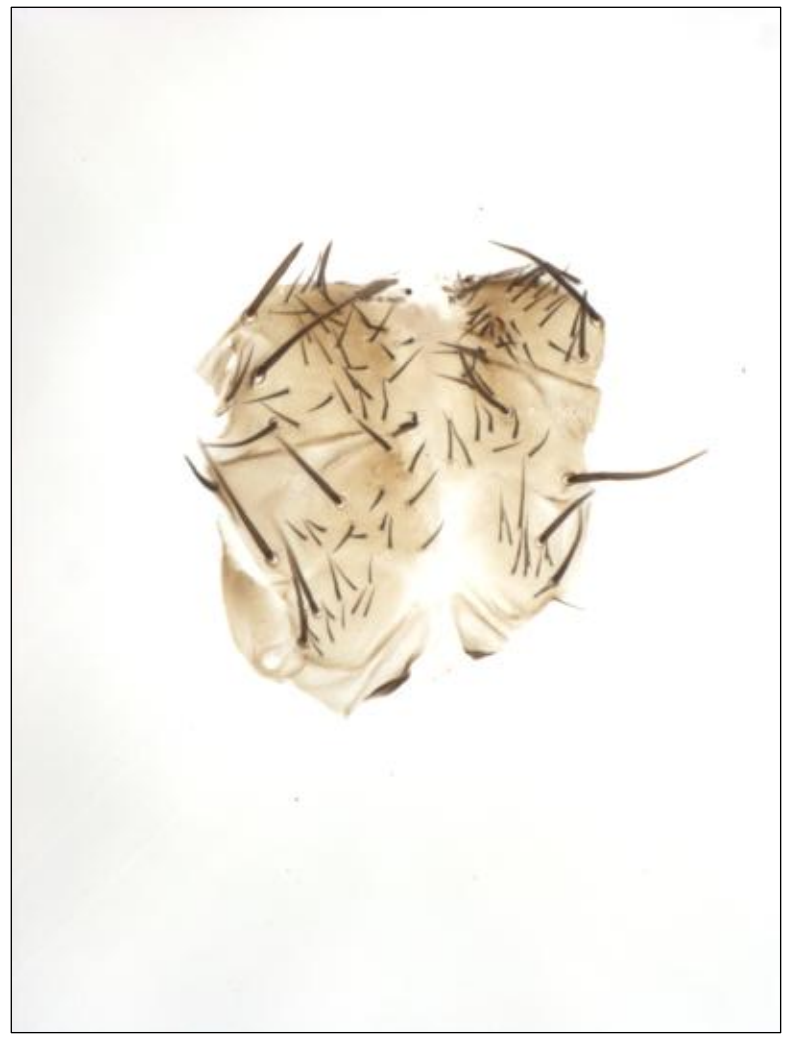

pnr>yki-RNAi-1, wts-RNAi-1 25C

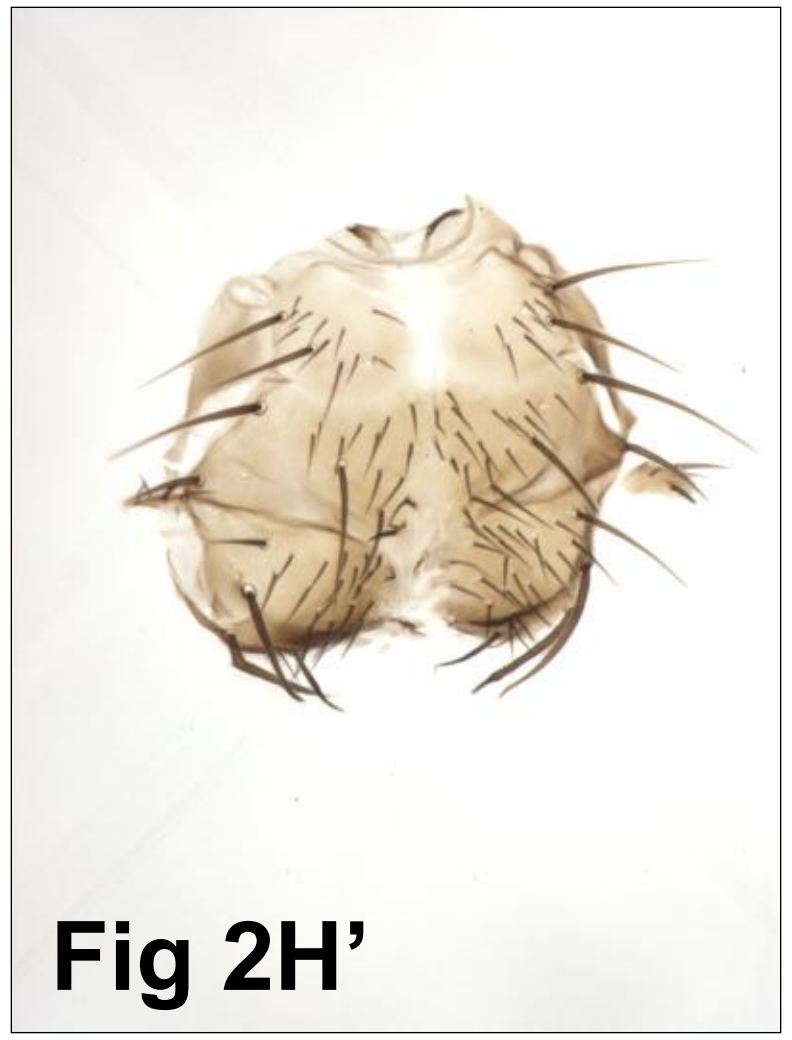

Fig 2H’

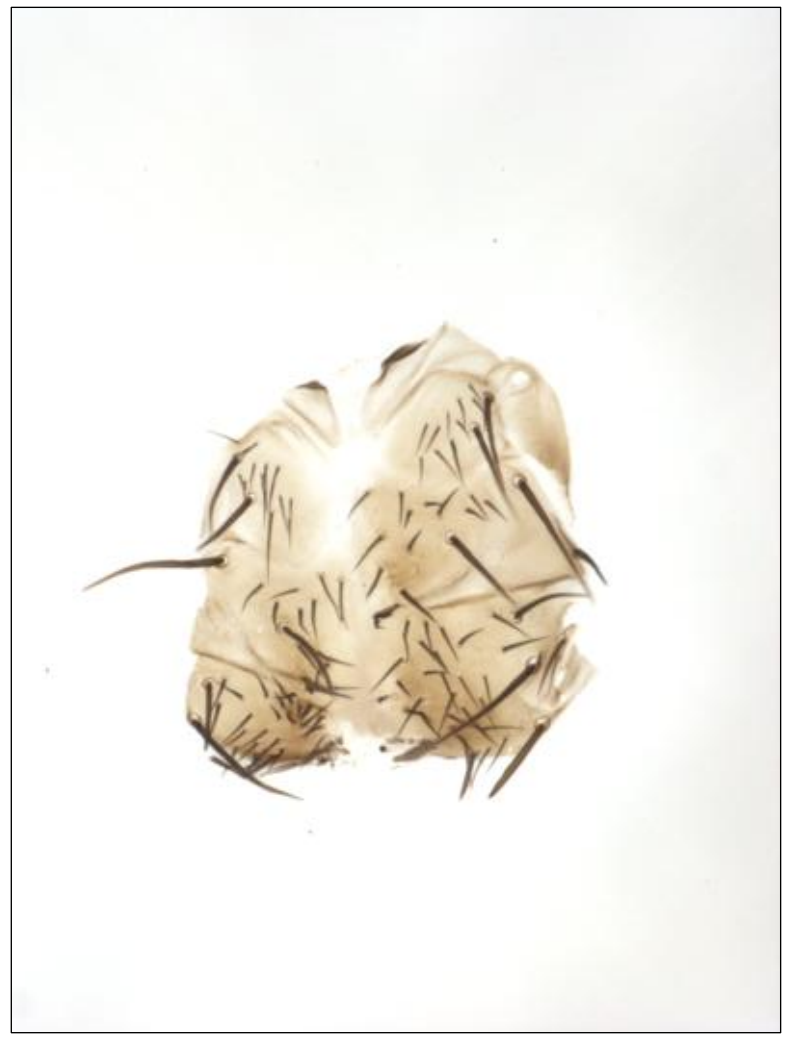

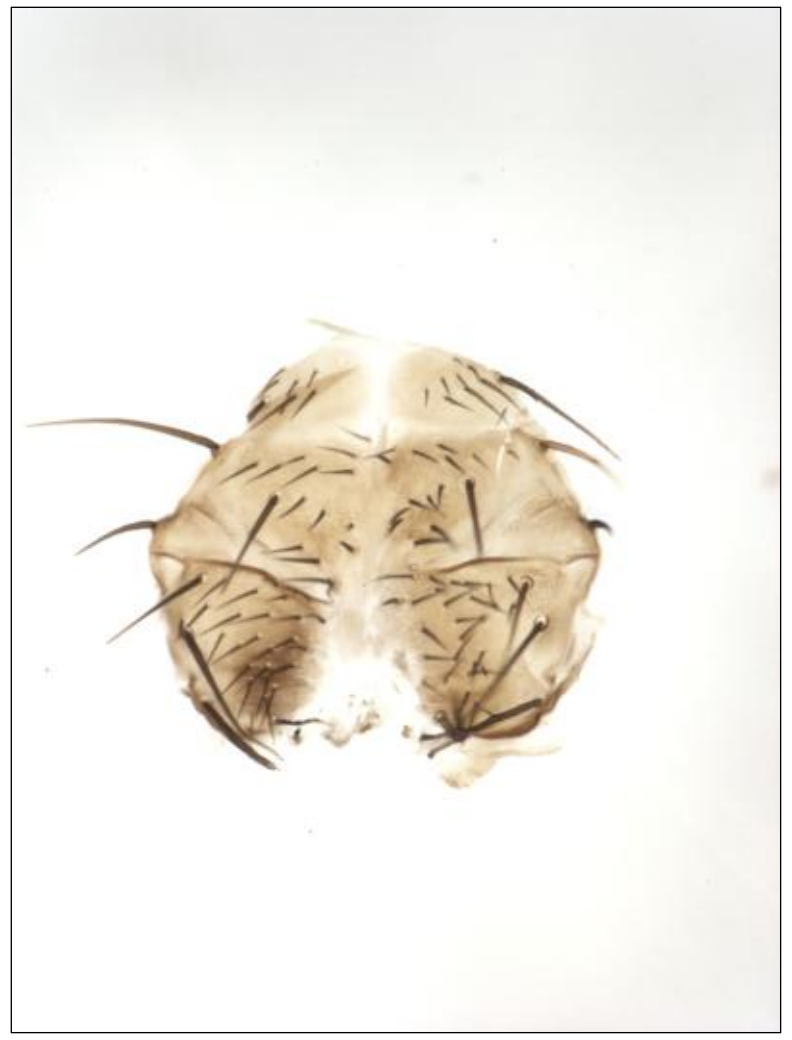

pnr>yki-RNAi-2 25C

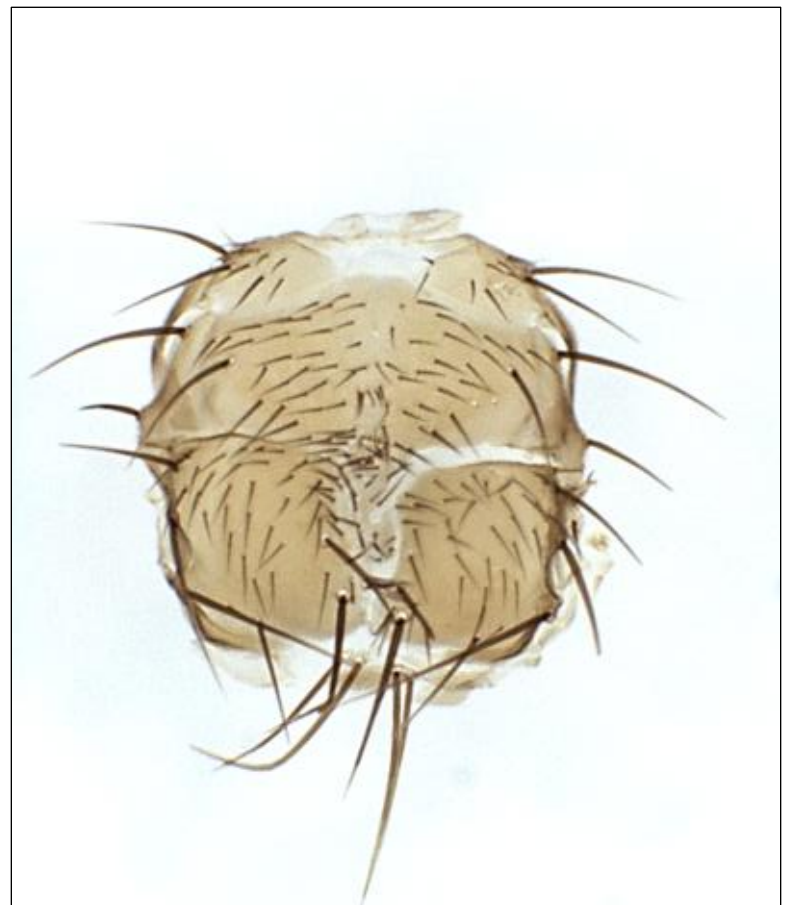

Fig 2I

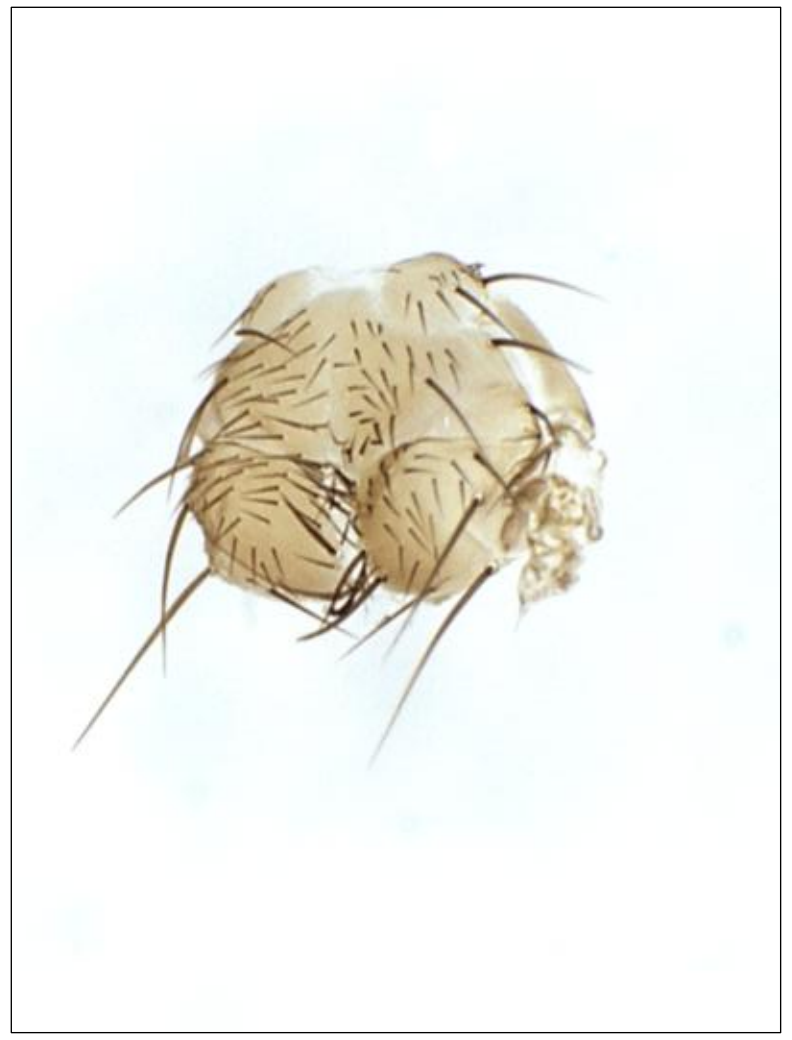

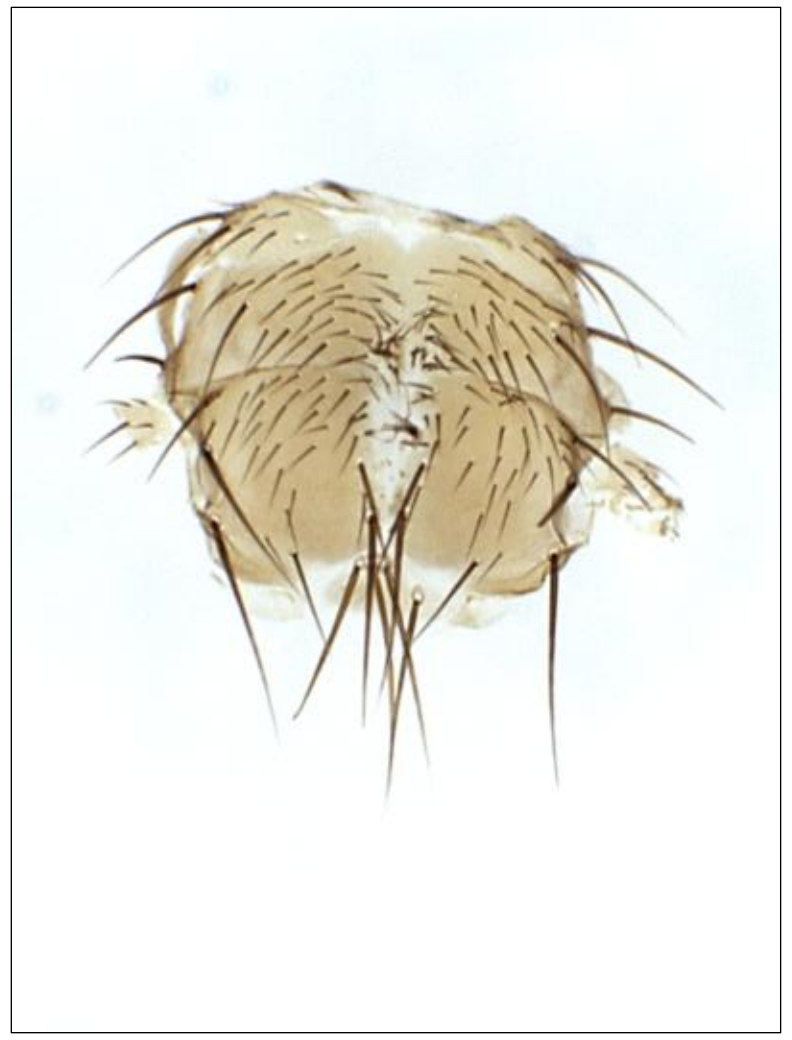

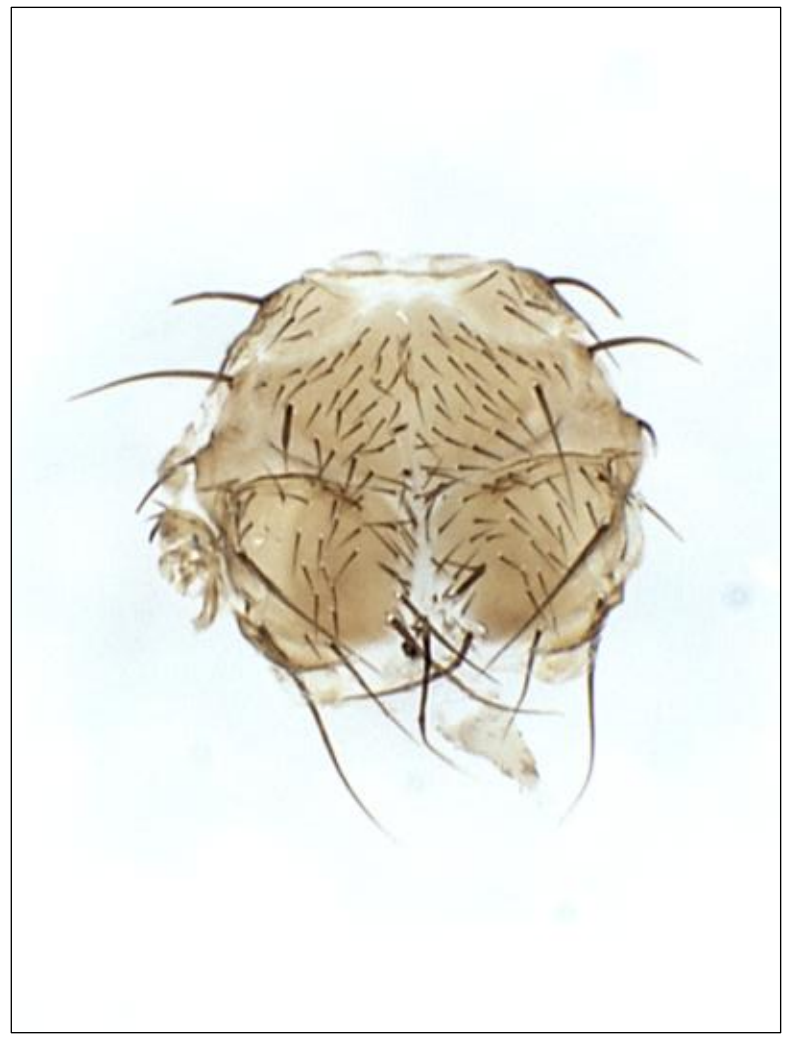

pnr>yki-RNAi-2, wts-RNAi-1 25C

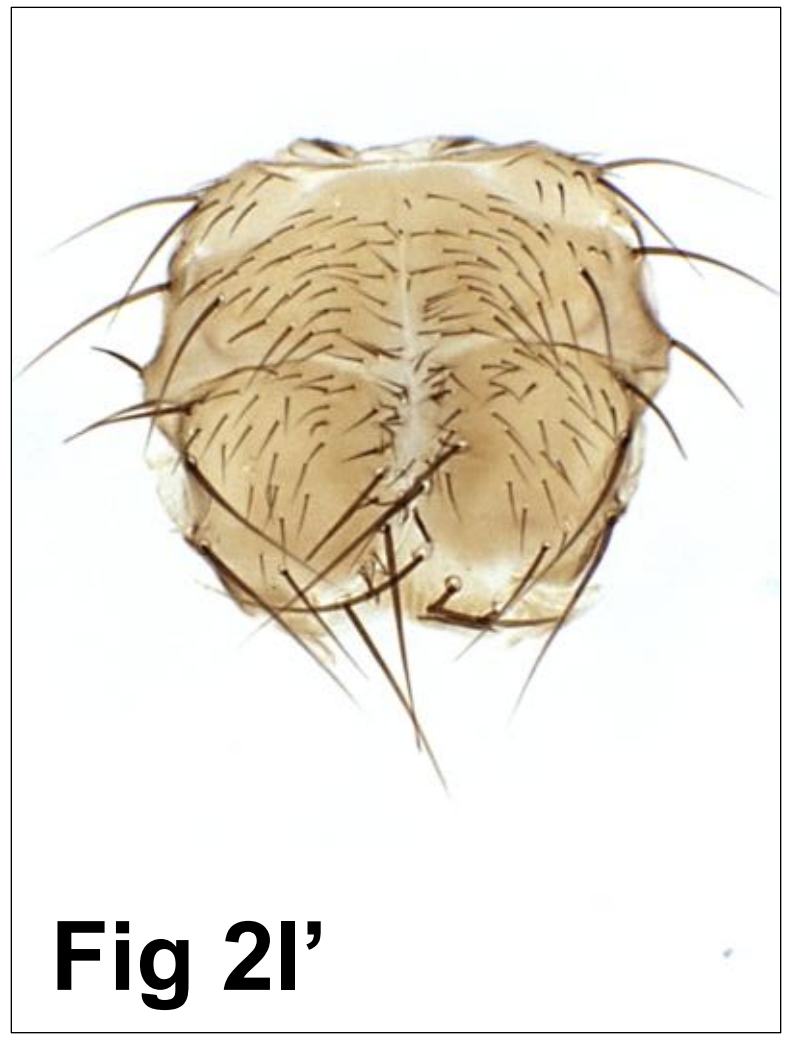

Fig 2I’

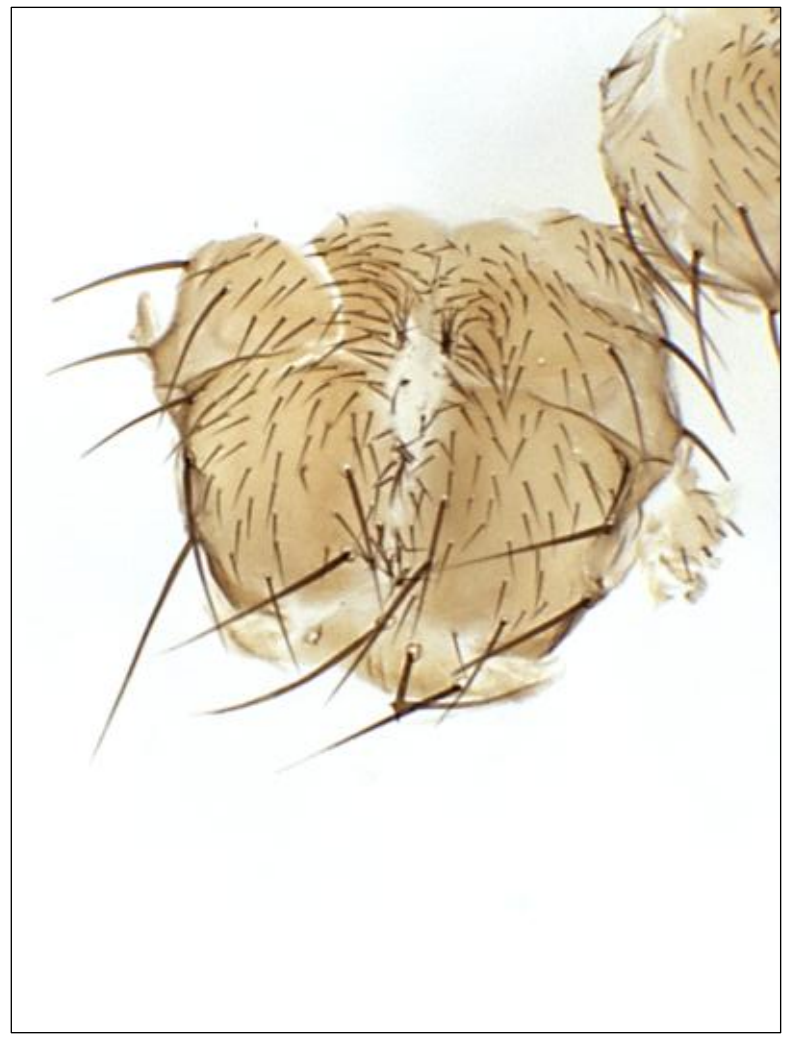

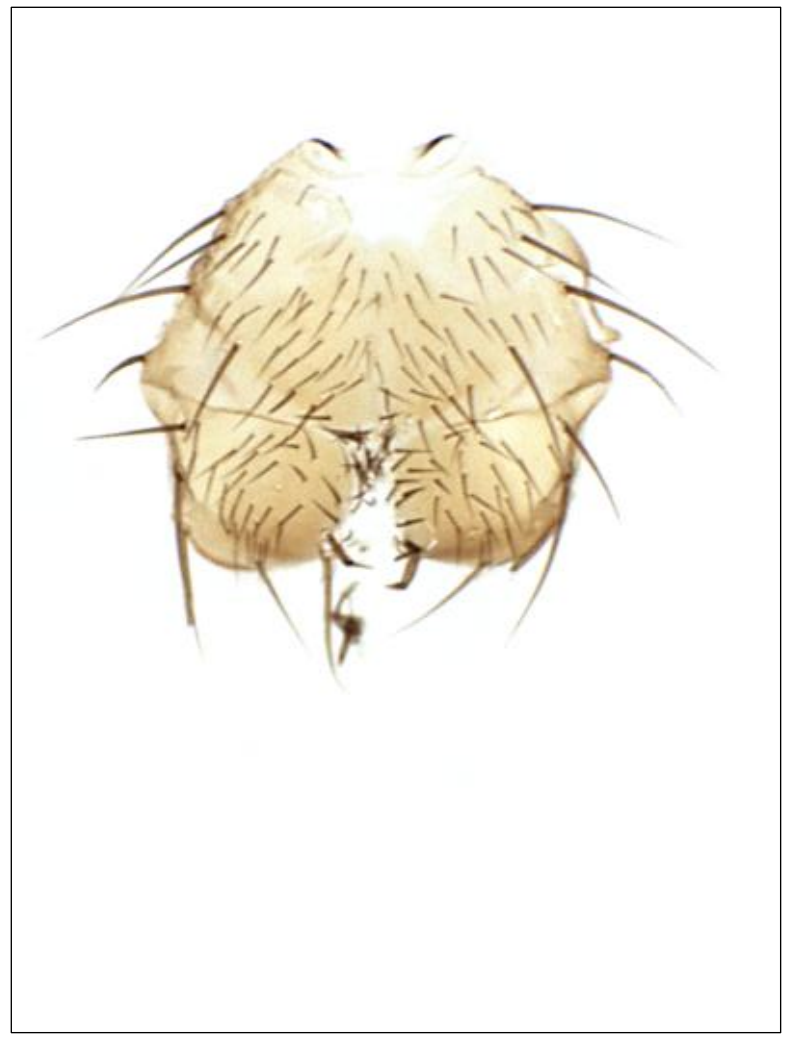

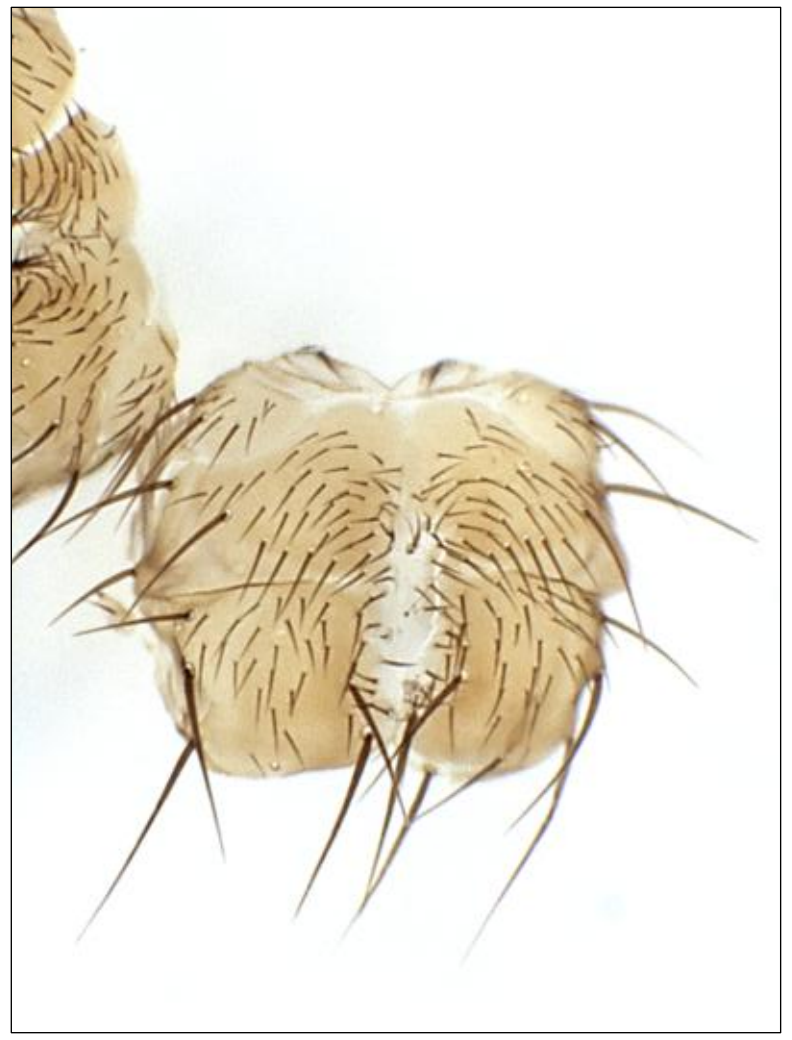

pnr>sd-RNAi-1 25C

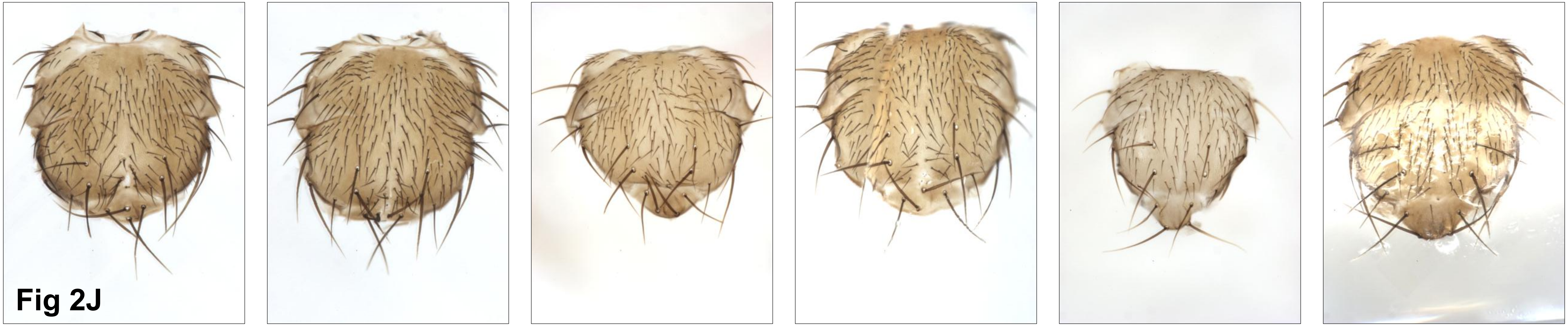

Fig 2J

pnr>sd-RNAi-1, wts-RNAi-1 25C

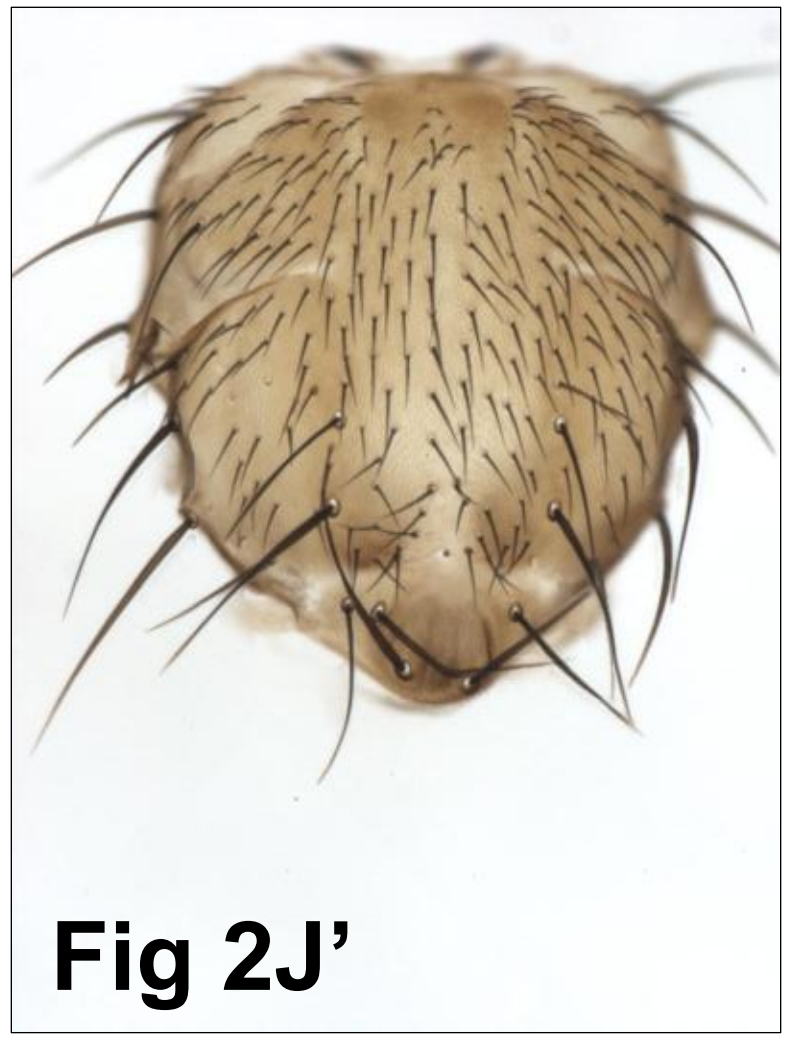
Fig 2J’

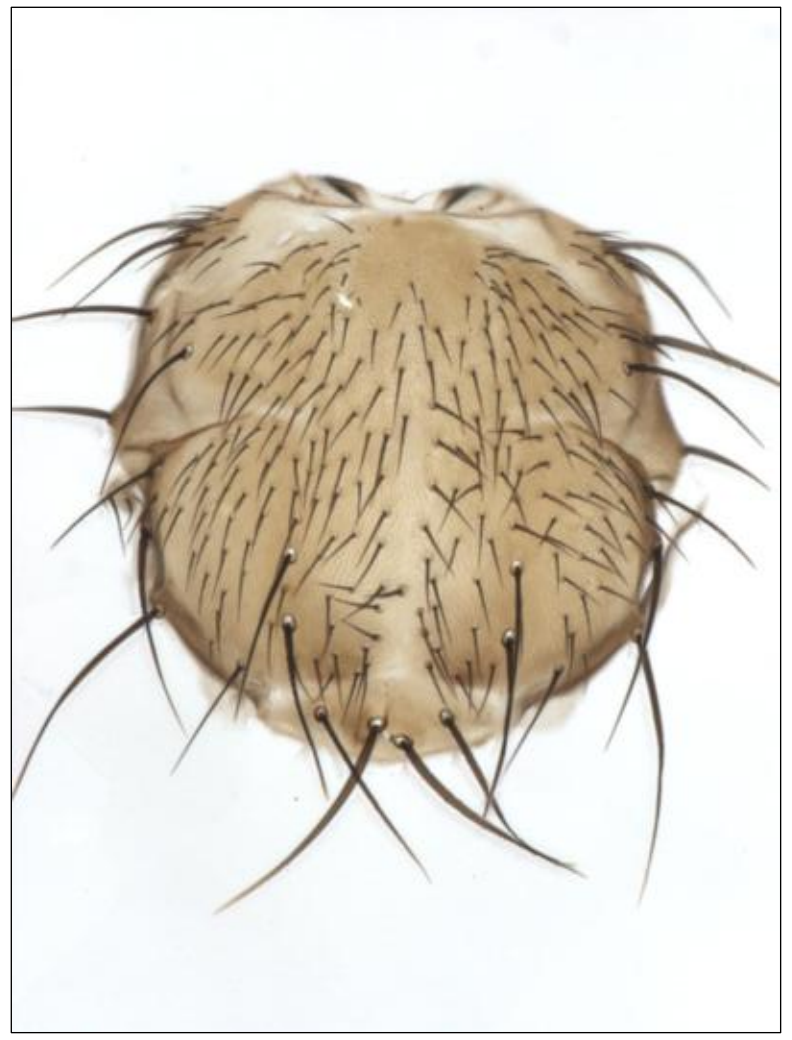

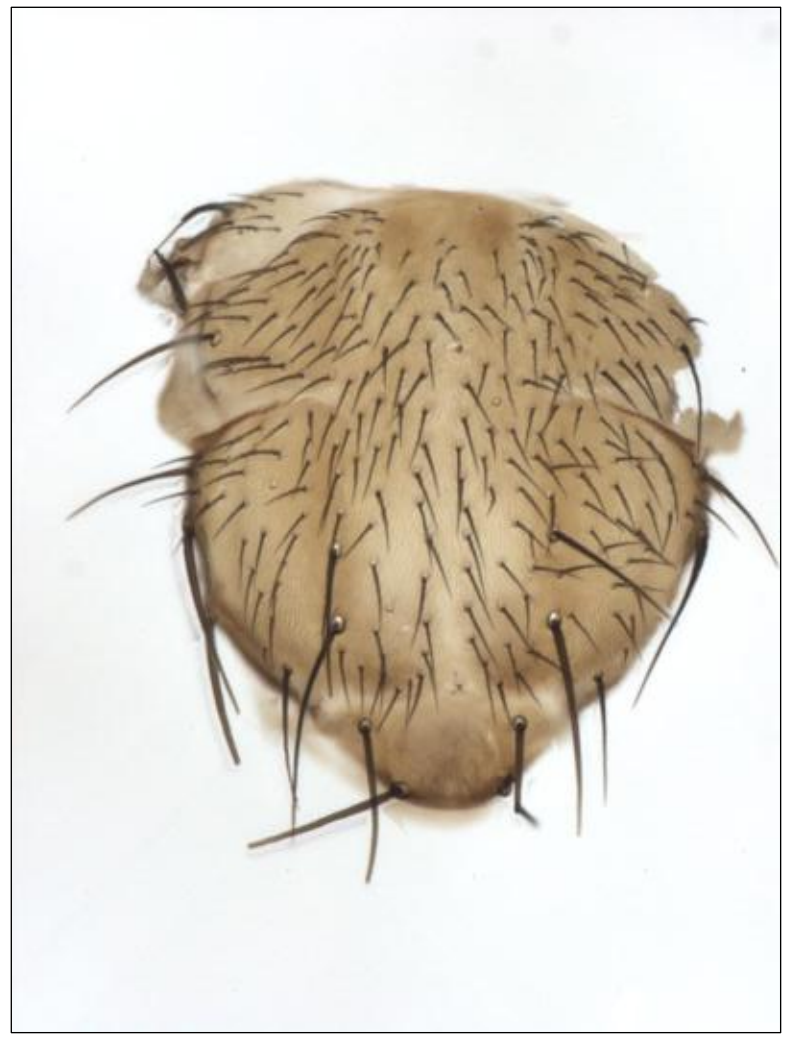

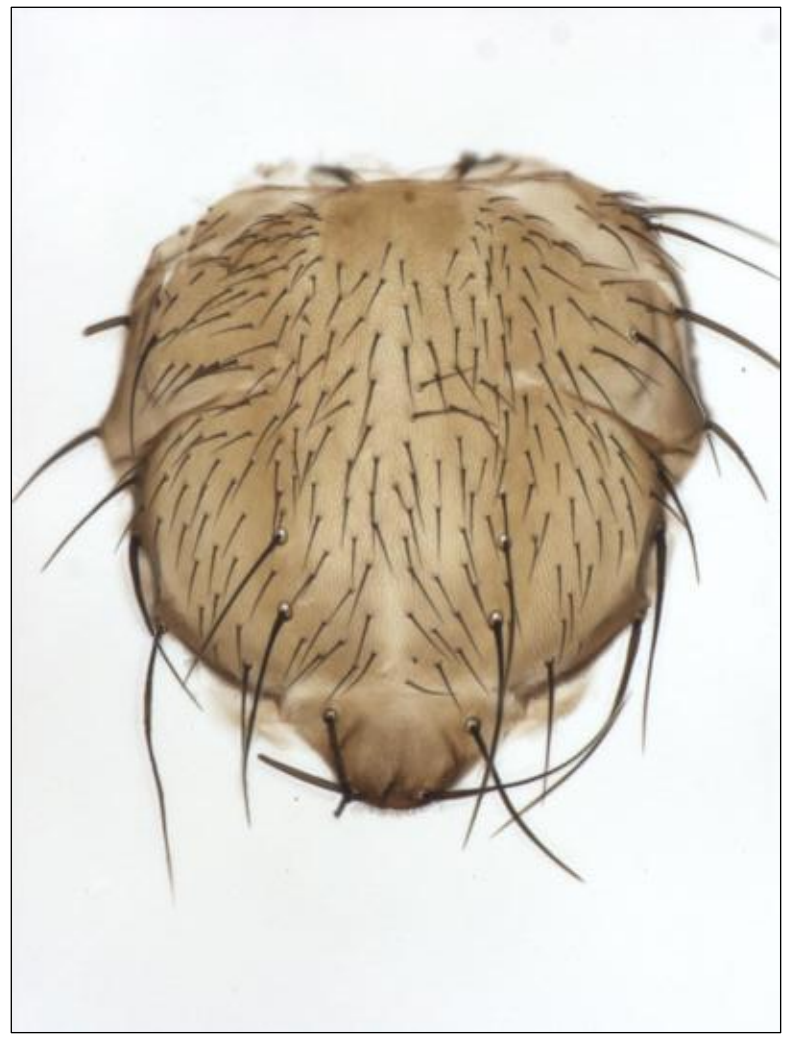

pnr>sd-RNAi-2 25C

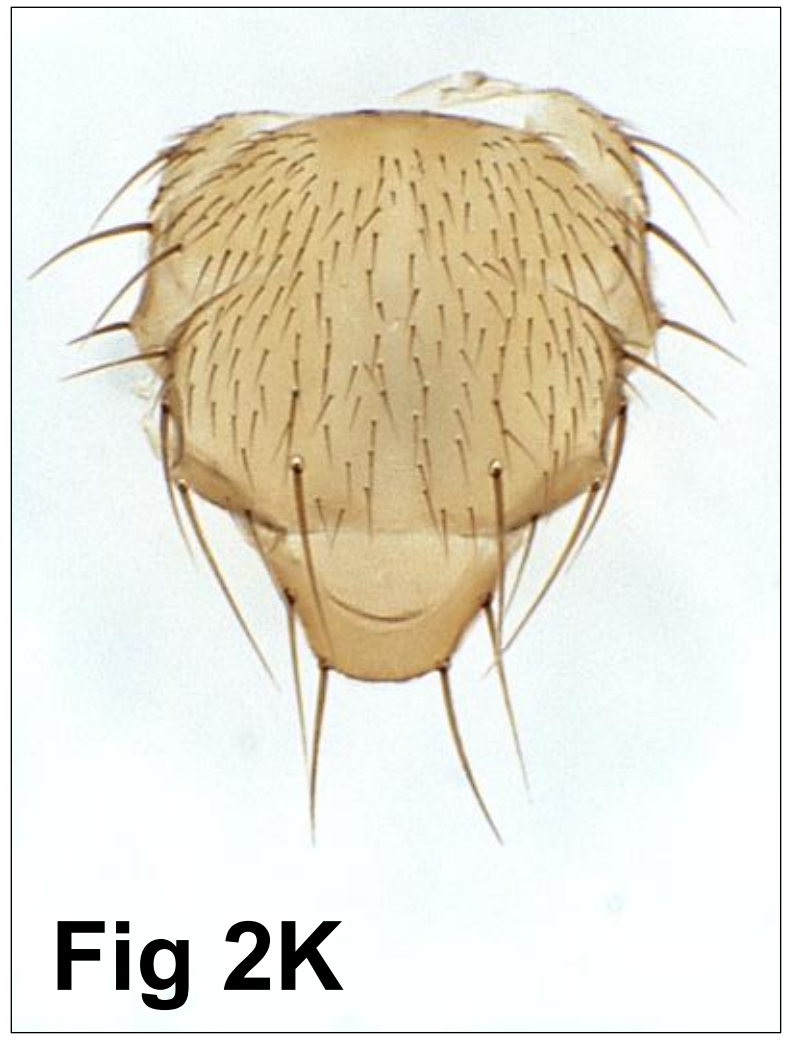

Fig 2K

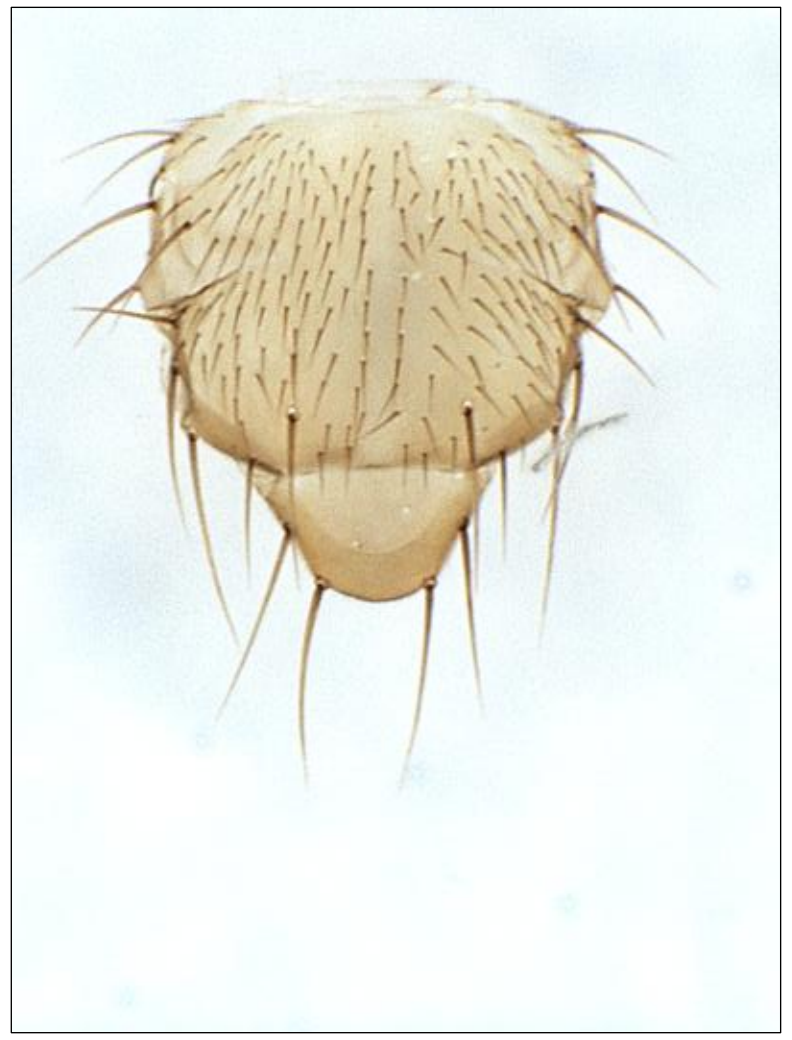

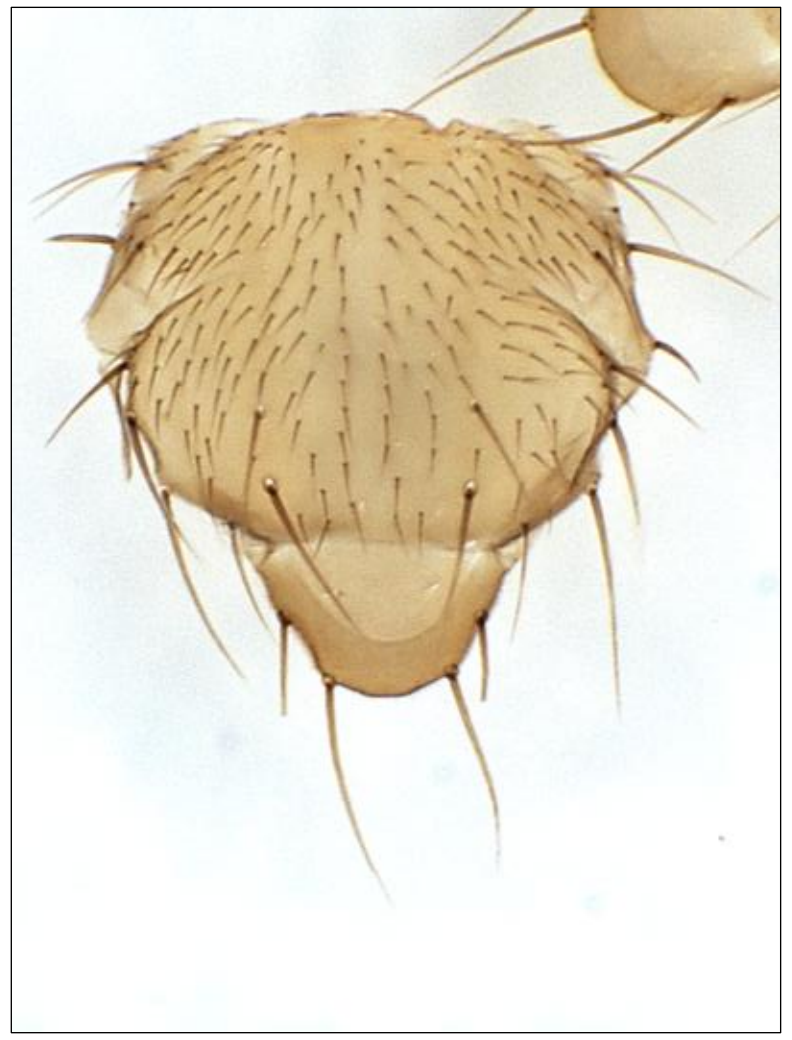

pnr>sd-RNAi-2, wts-RNAi-1 25C

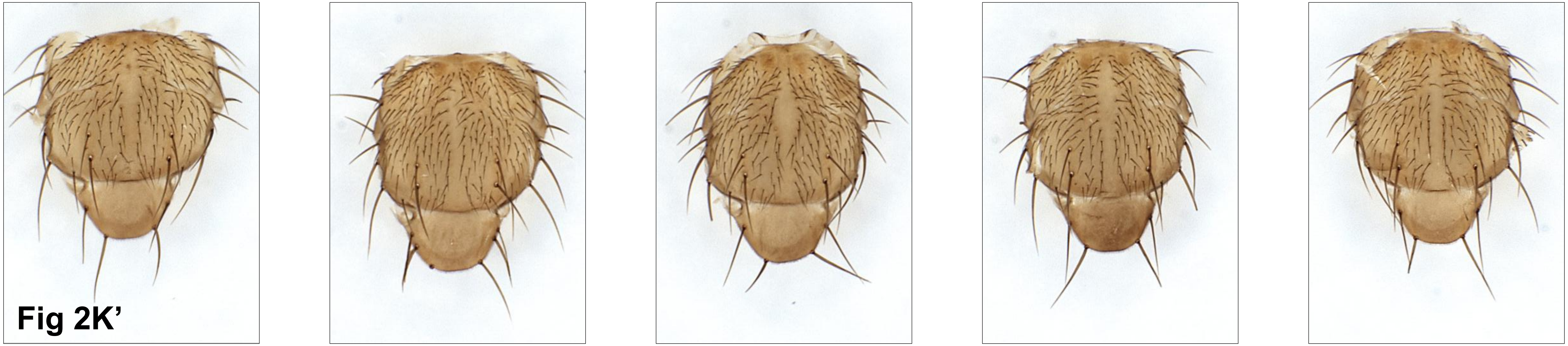

Fig 2K’

FIGURE 3

pnr>MOB1A 29C

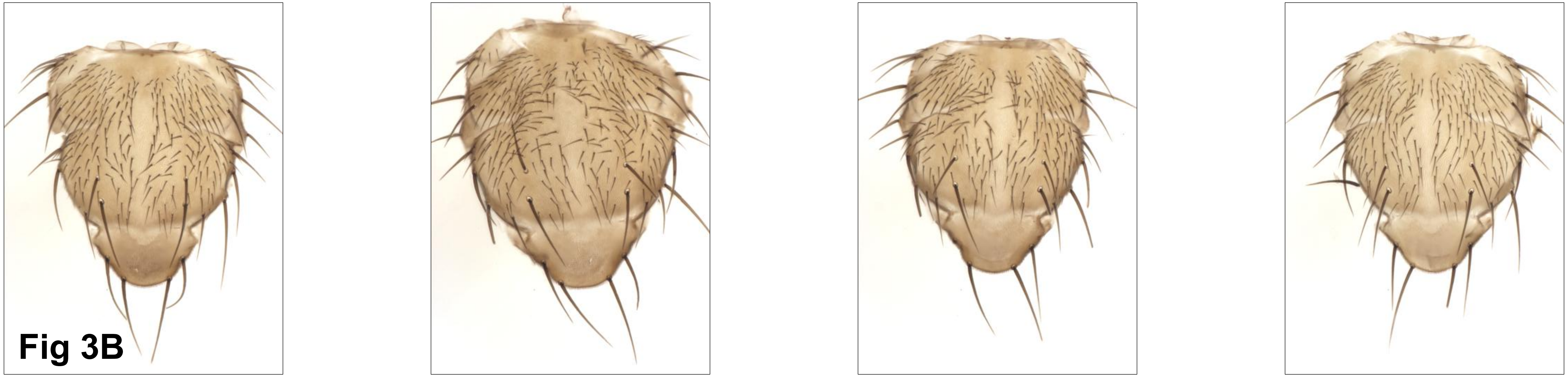

Fig 3B

pnr>MOB1B 29C

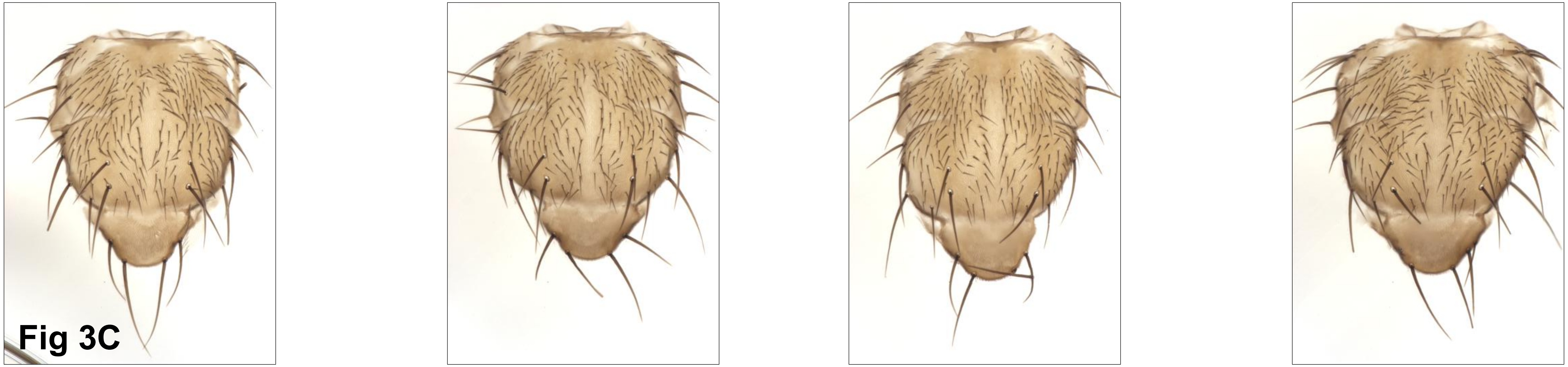

pnr>mats-RNAi-1, MOB1A 29C

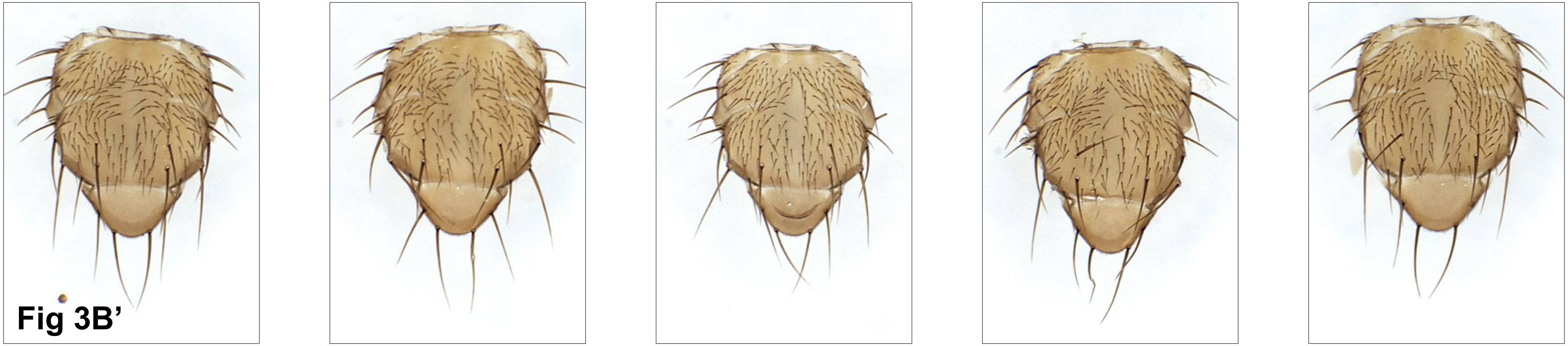

pnr>mats-RNAi-1, MOB1B 29C

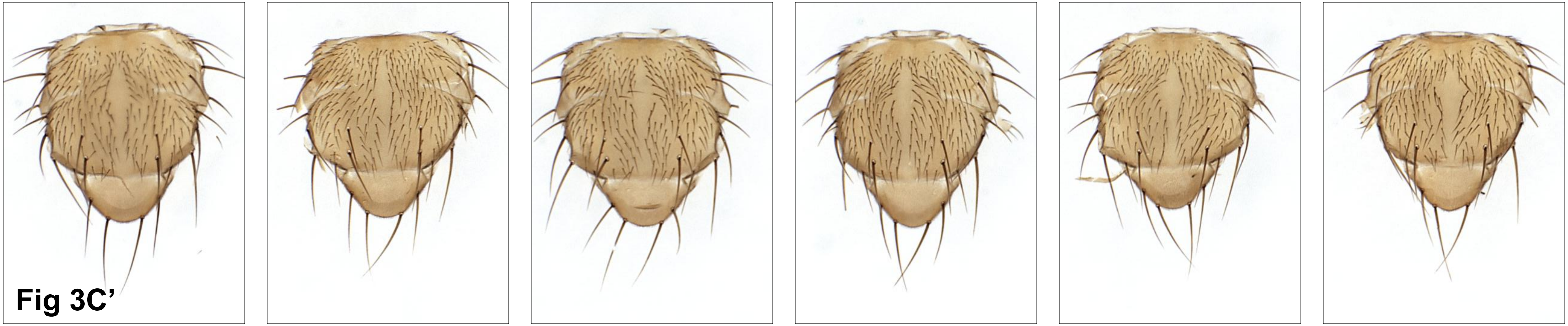

Fig 3C’

pnr>LATS1 RT

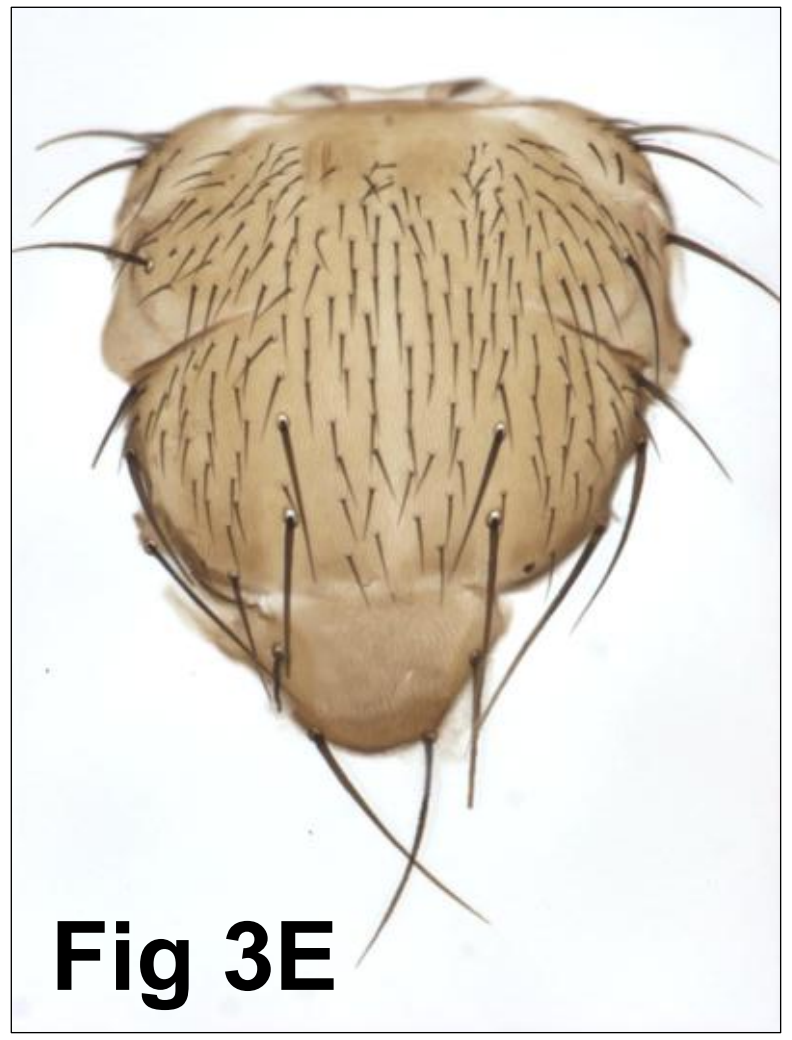

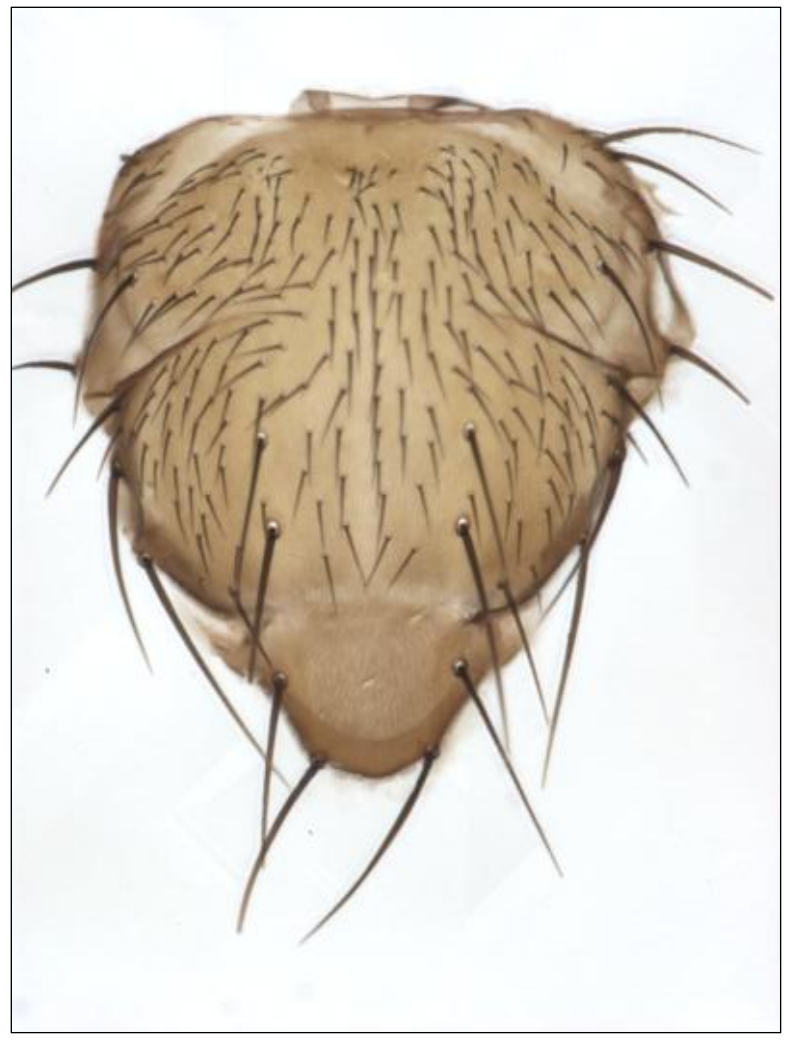

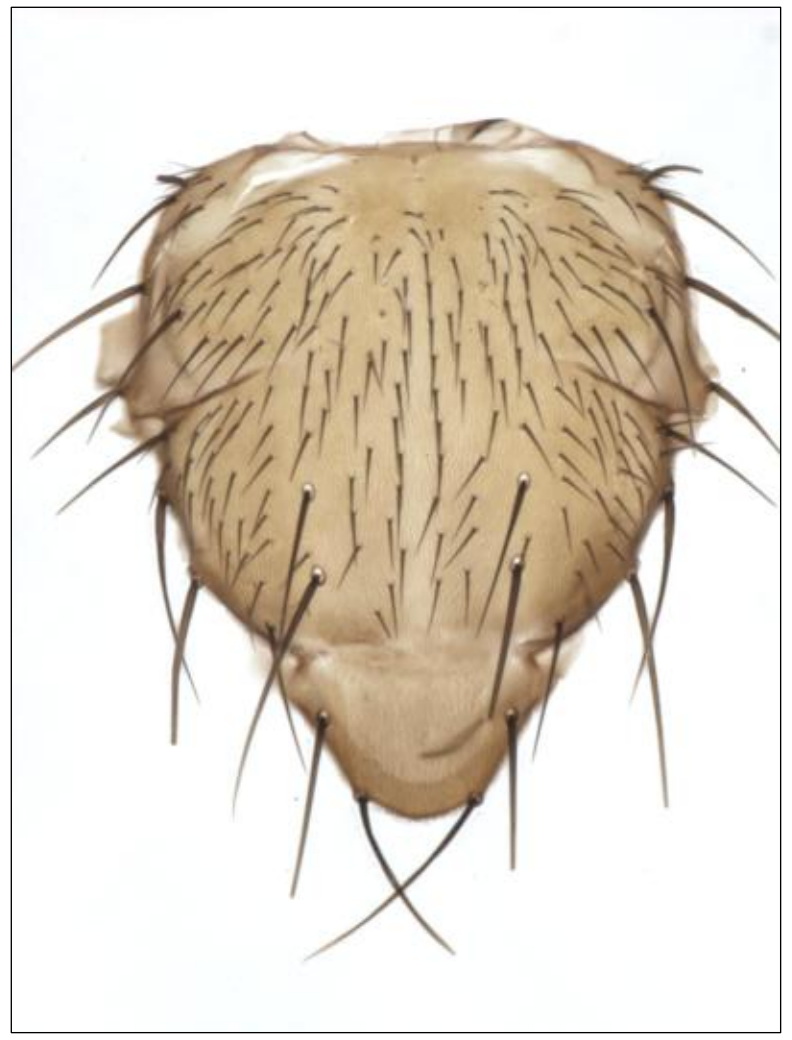

pnr>LAT2 RT

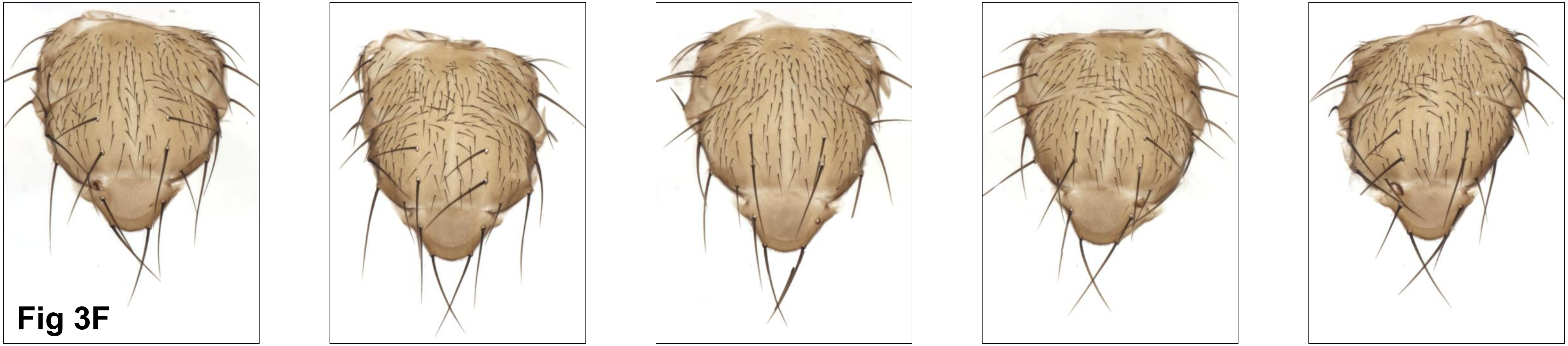

Fig 3F

pnr>wts-RNAi-1, LATS1 RT

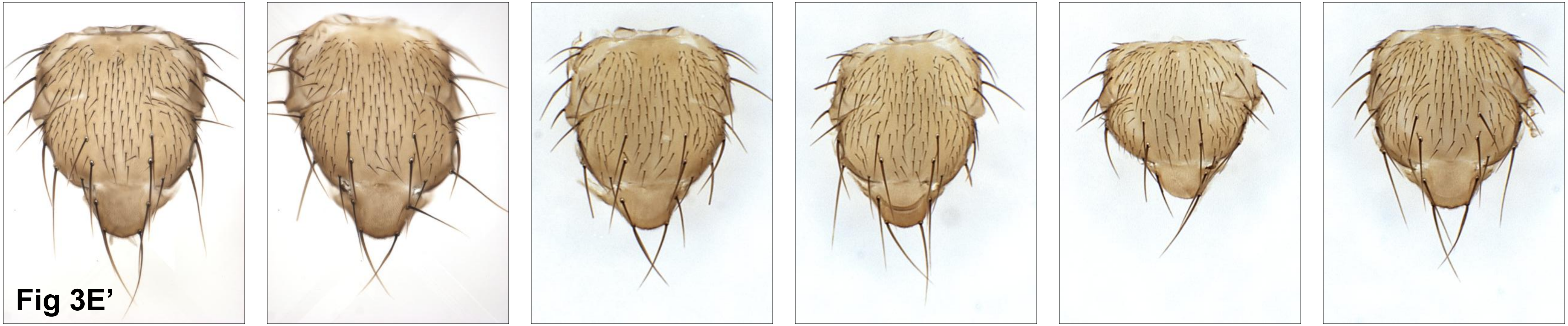

Fig 3E’

pnr>wts-RNAi-1, LATS2 RT

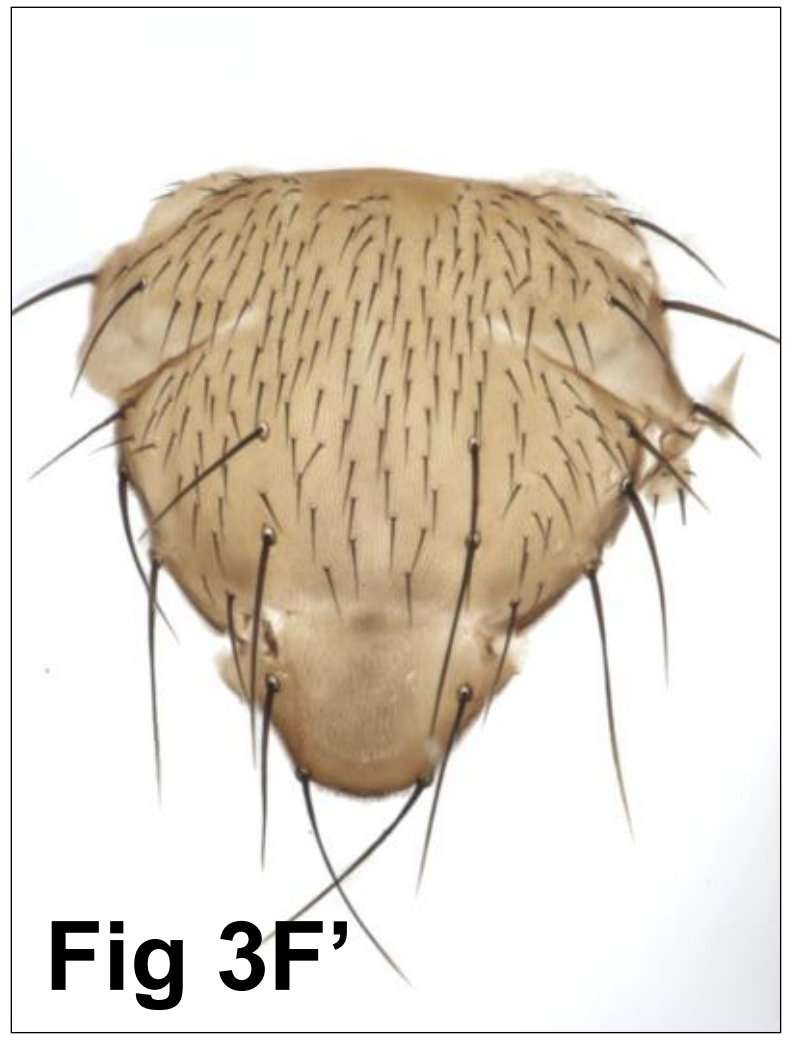

Fig 3F’

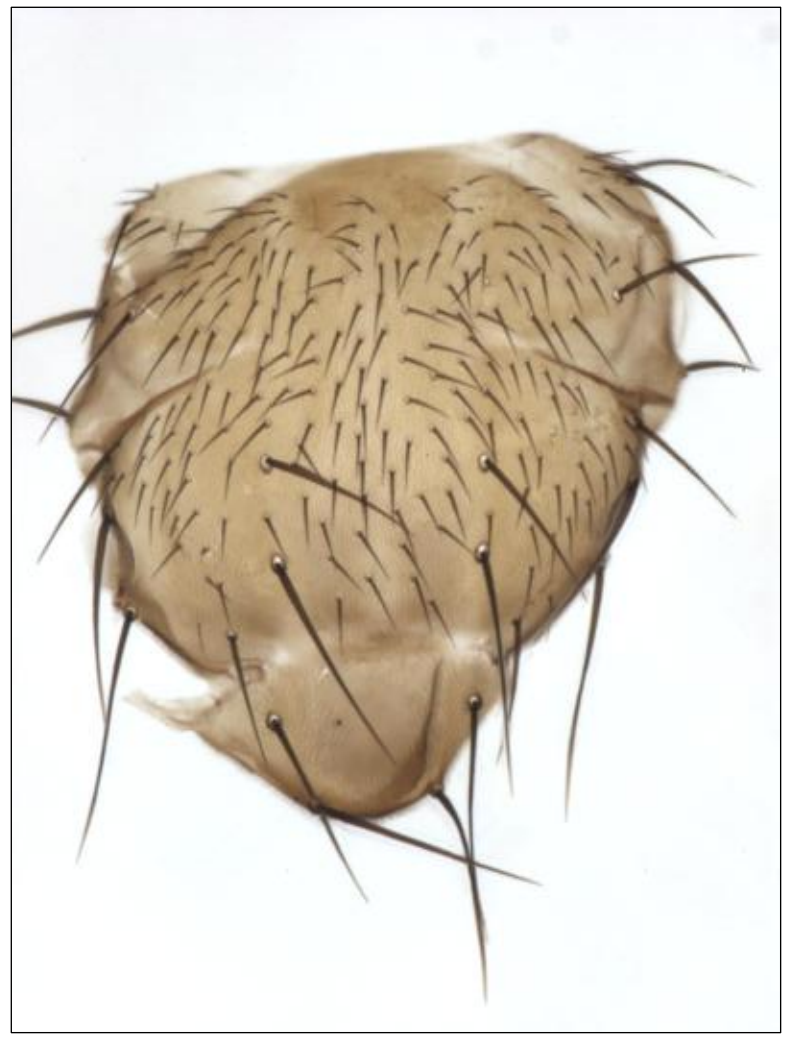

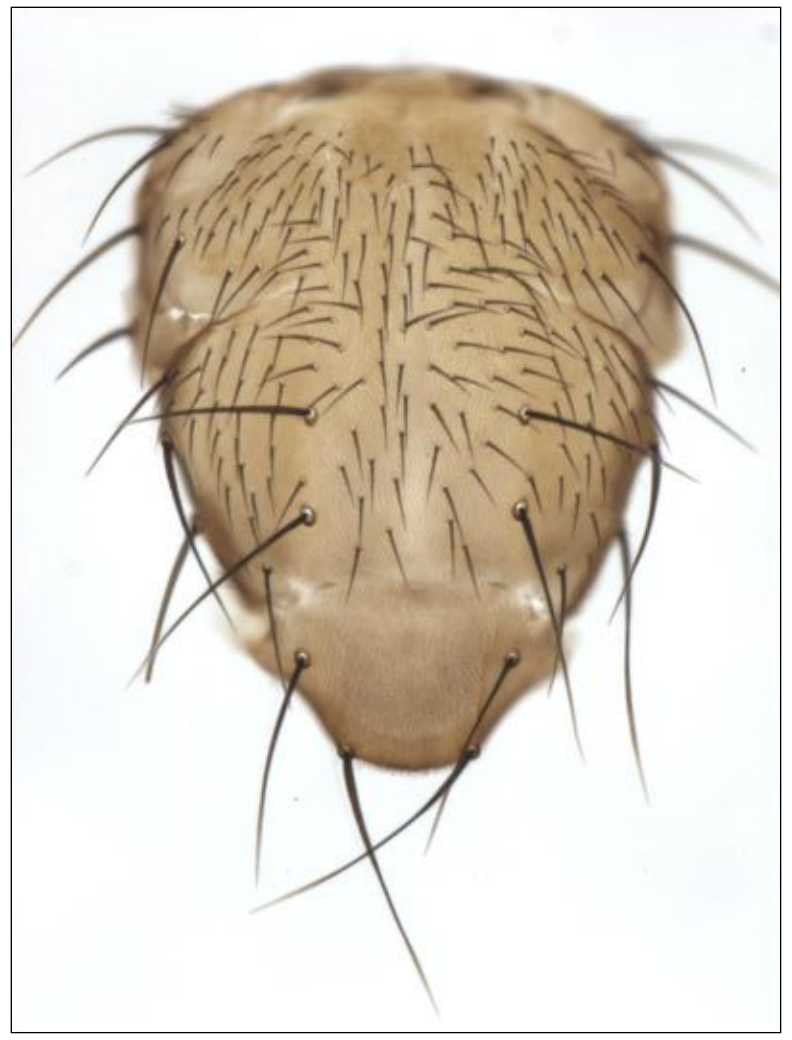

pnr>YAP1 25C

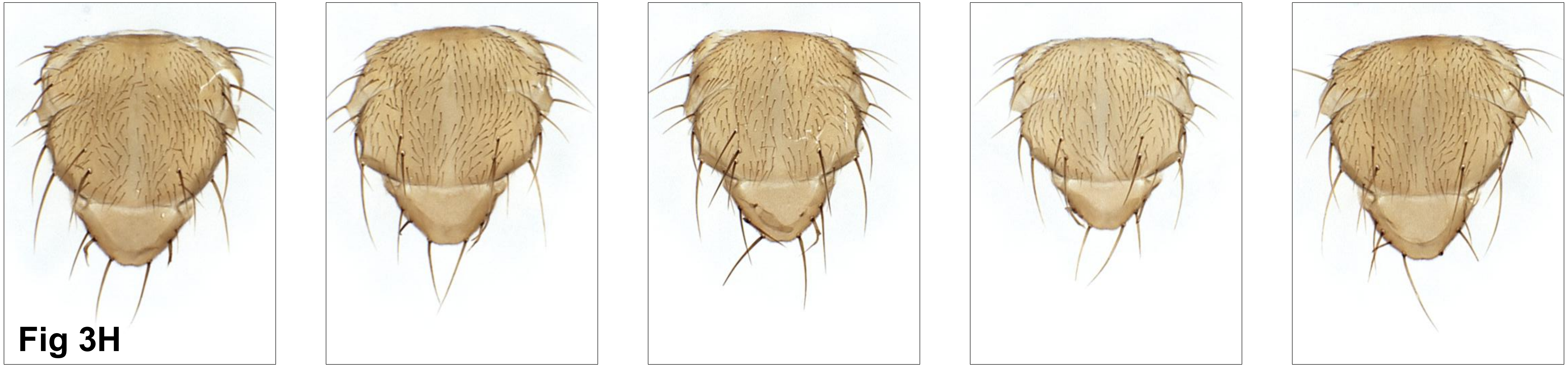

pnr>YAP1, yki-RNAi-1 25C

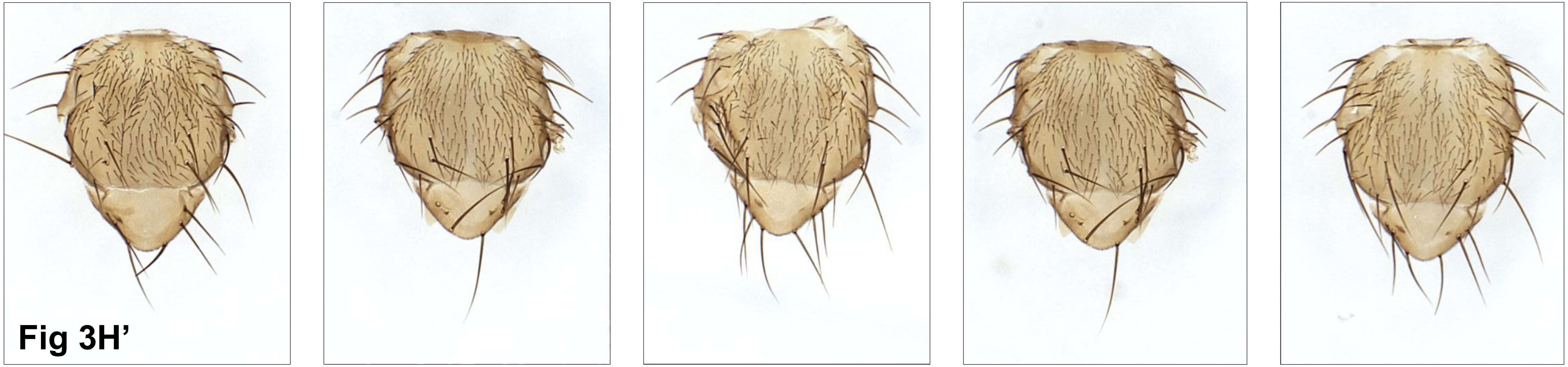

Fig 3H’

Notum Images

Figure 6 and Supplemental Figure 9

DEG concordant

pnr>stw-RNAi 29C

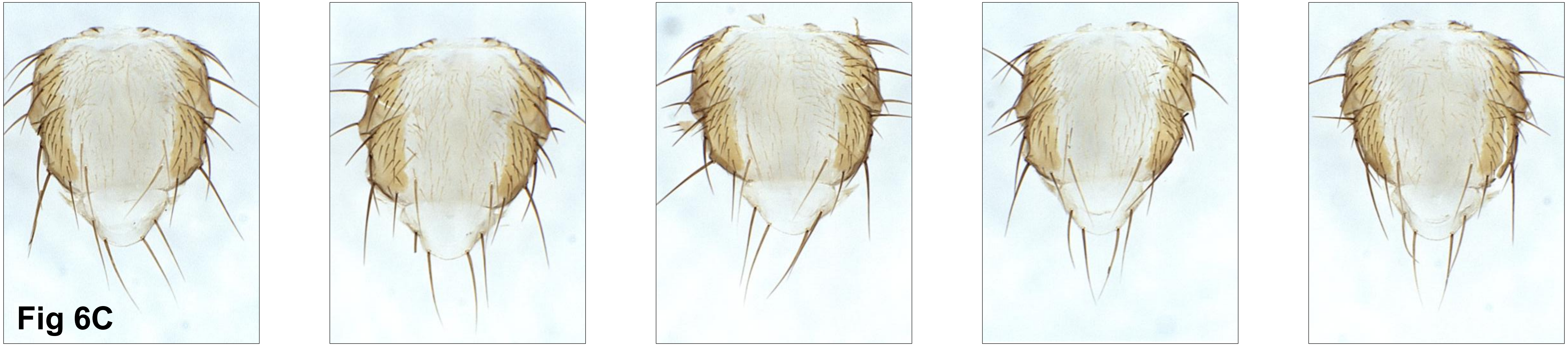

pnr>wts-RNAi-1, stw-RNAi 29C

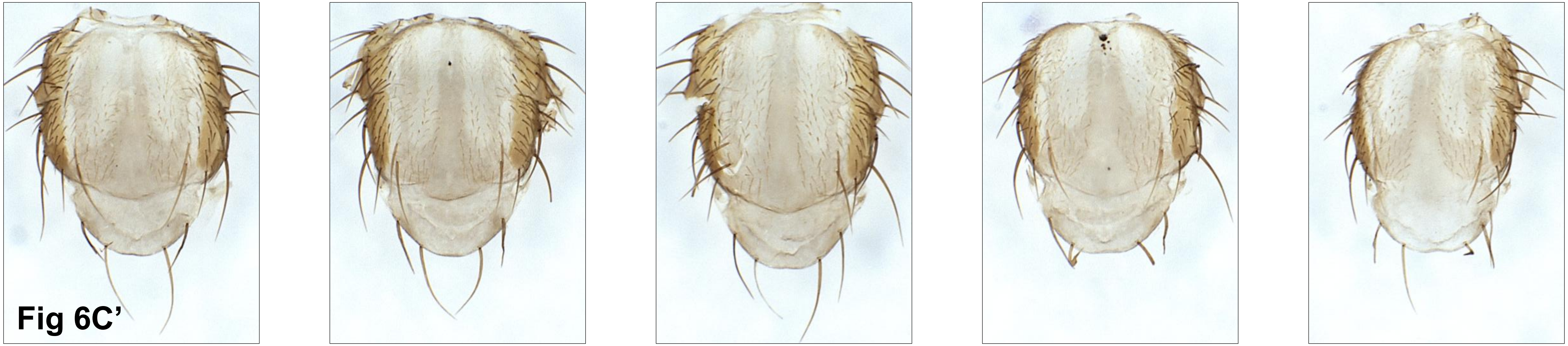

pnr-Lk6-RNAi 29C

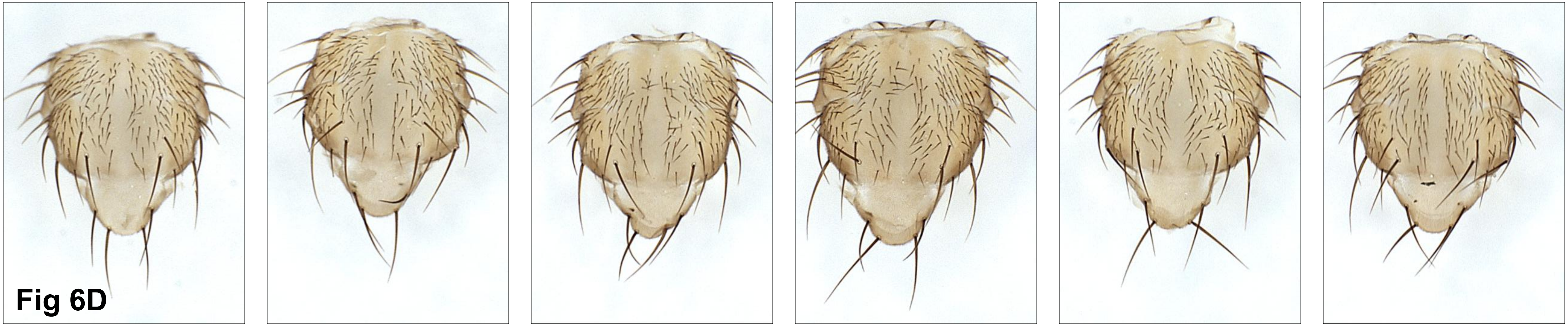

Fig 6D

pnr-wts-RNAi-1, Lk6-RNAi 29C

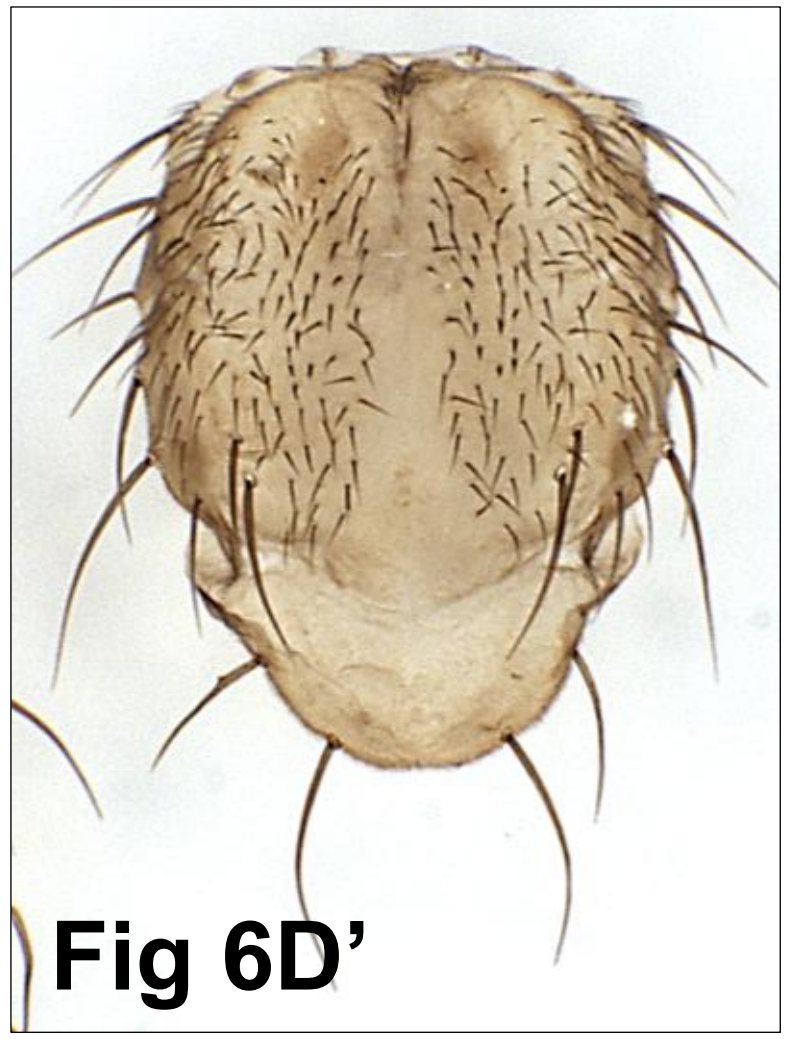

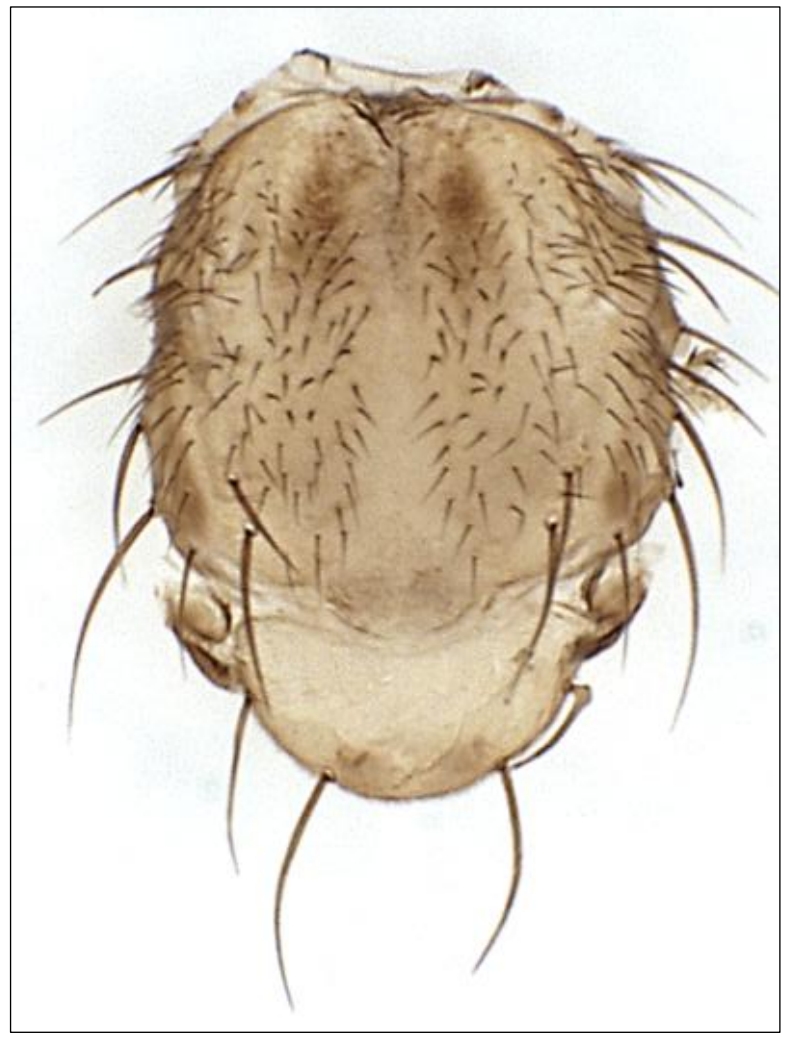

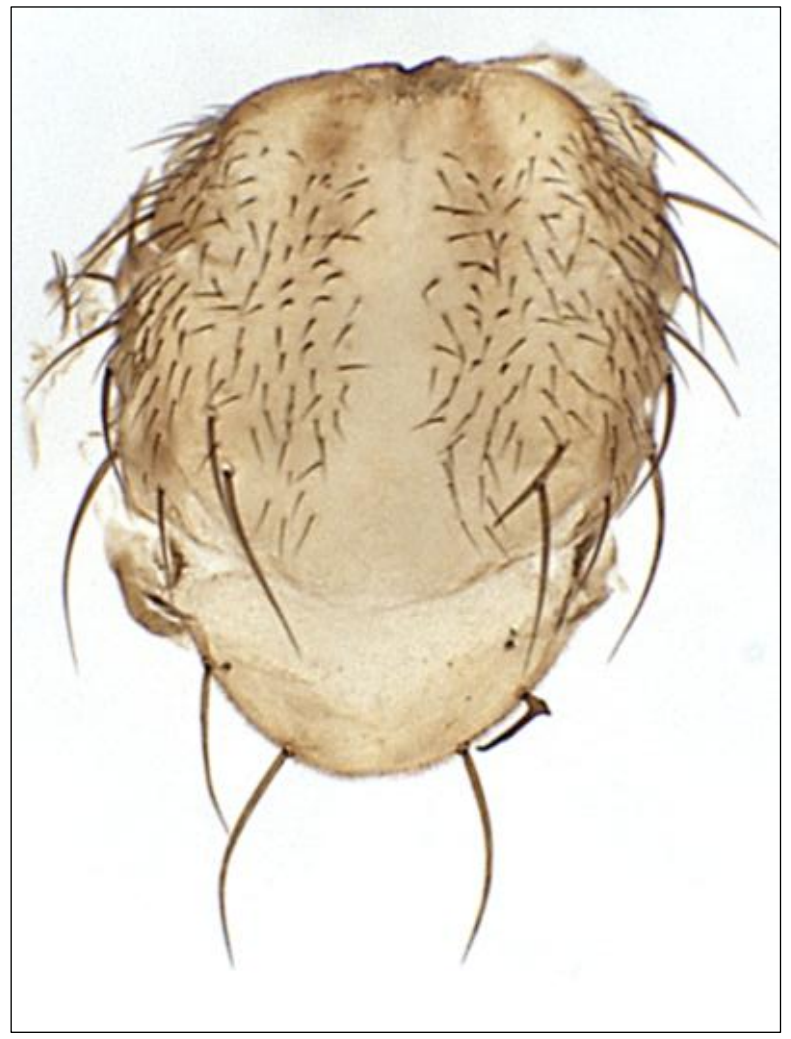

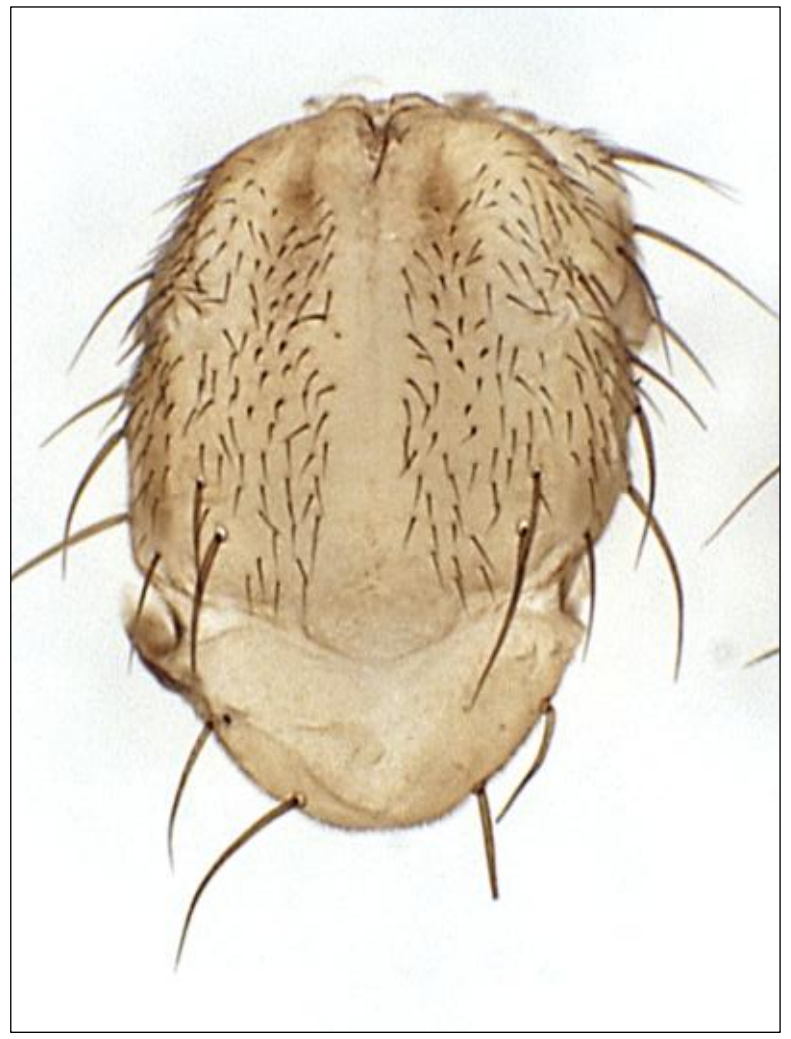

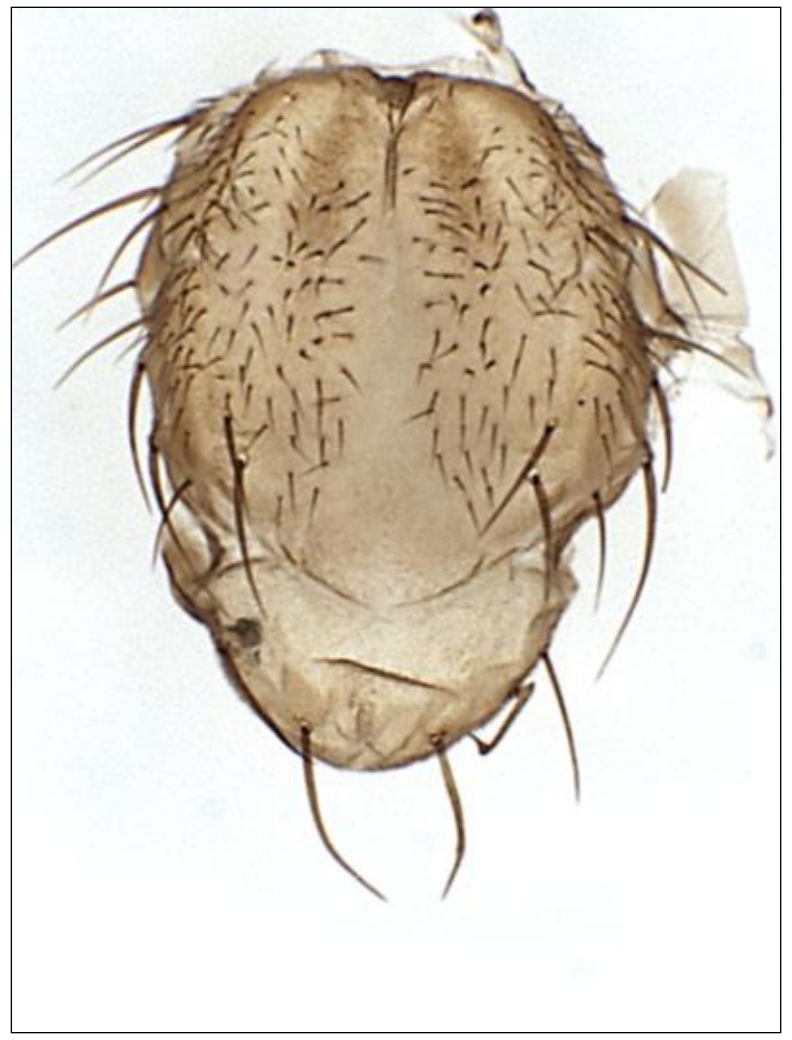

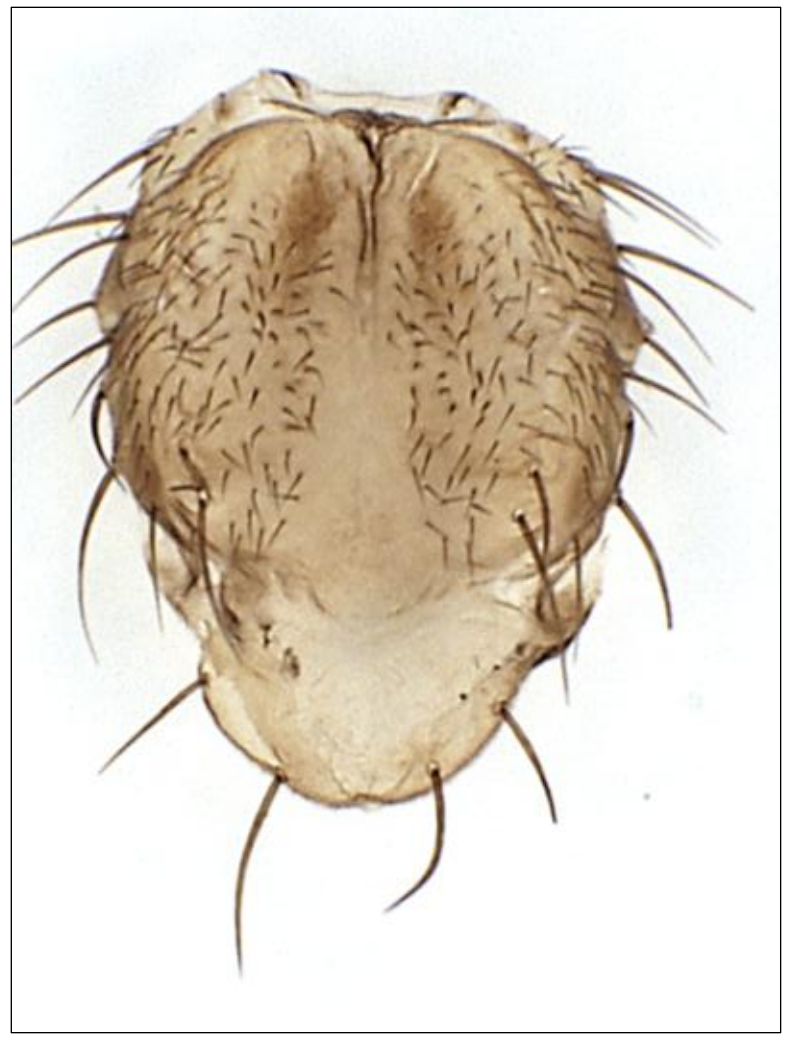

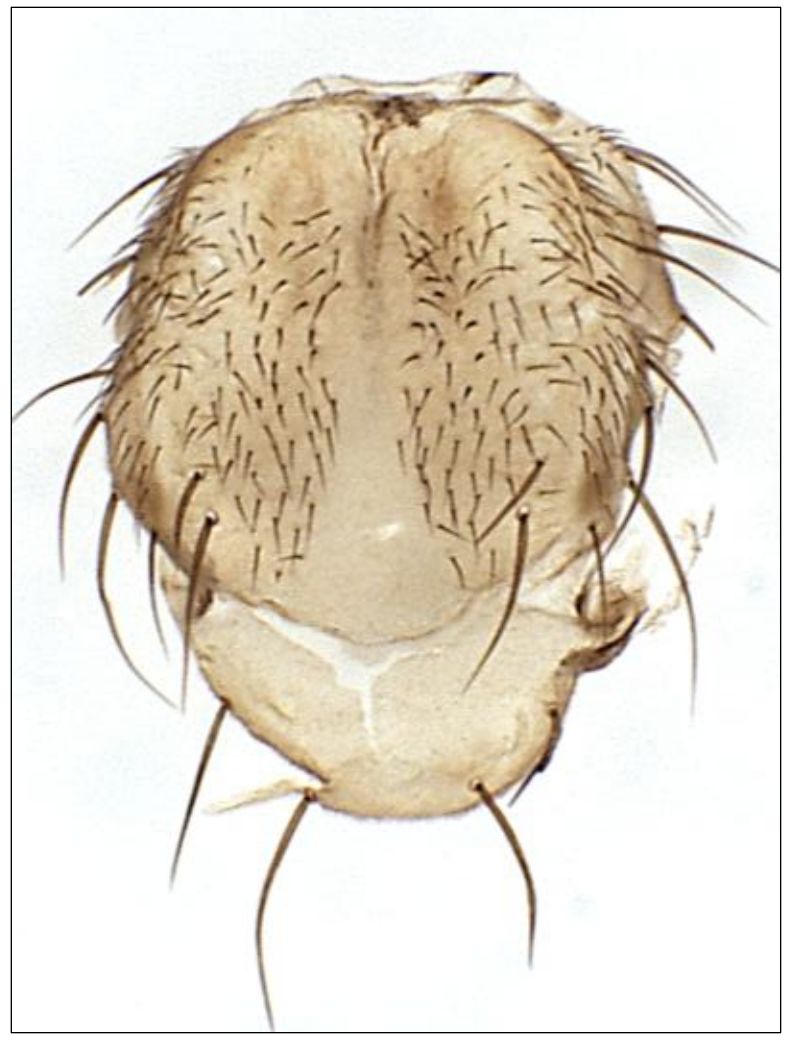

pnr>yellow-RNAi 29C

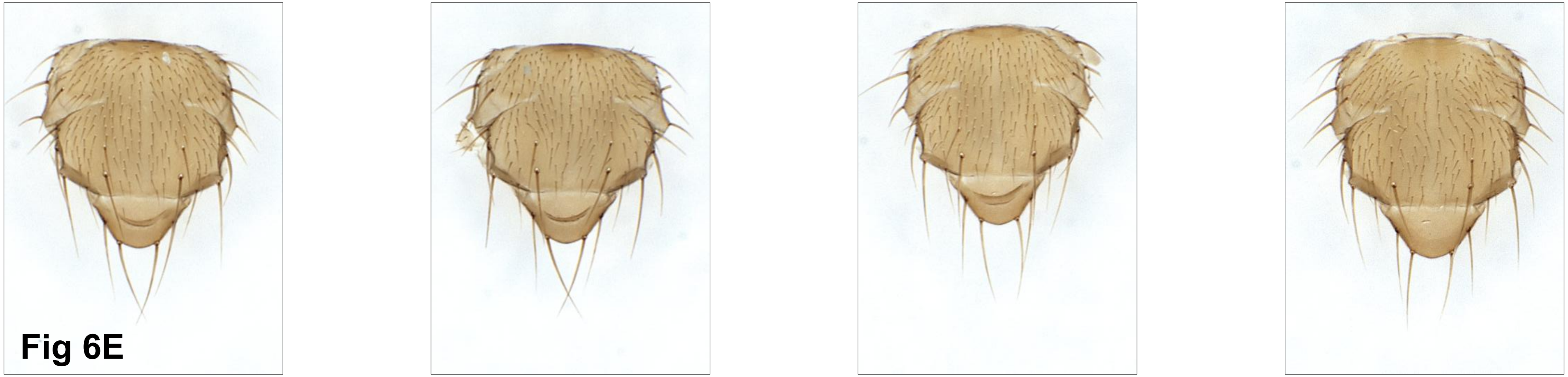

Fig 6E

pnr>wts-RNAi-1, yellow-RNAi 29C

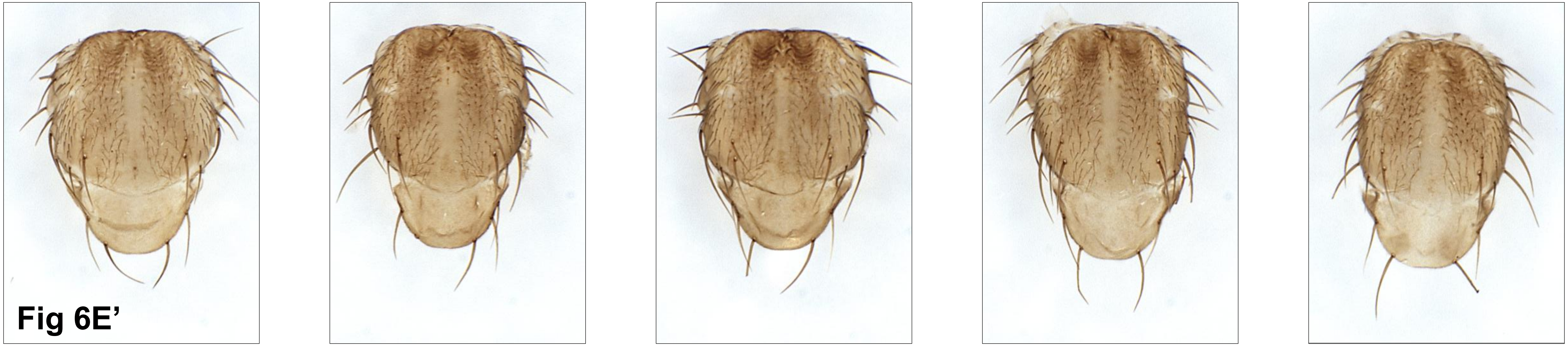

Fig 6E’

pnr>cib-RNAi 29C

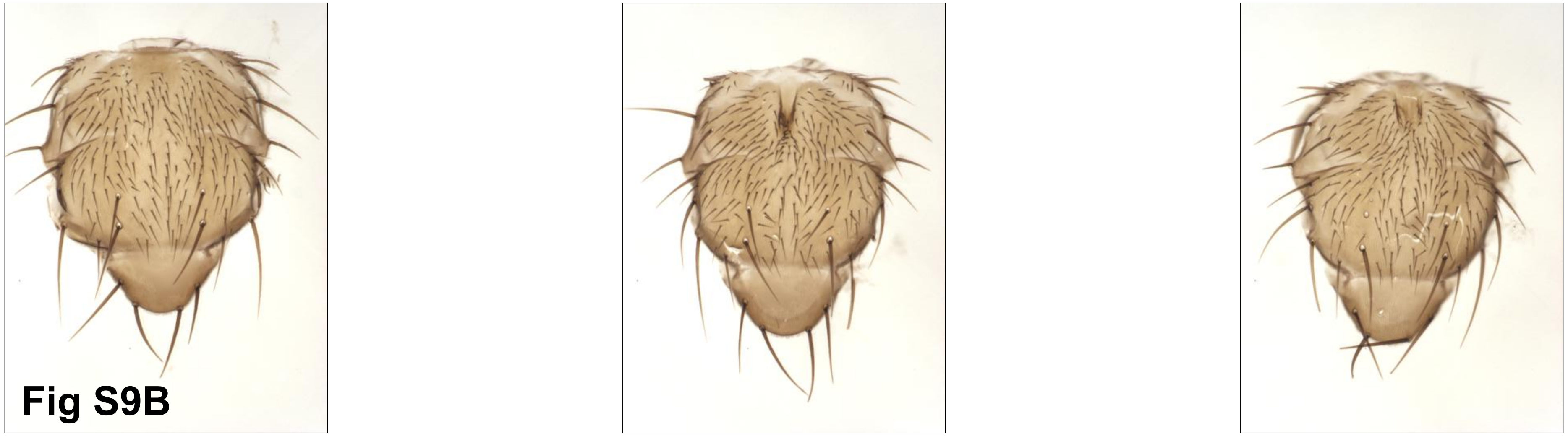

Fig S9B

pnr>wts-RNAi-1, cib-RNAi 29C

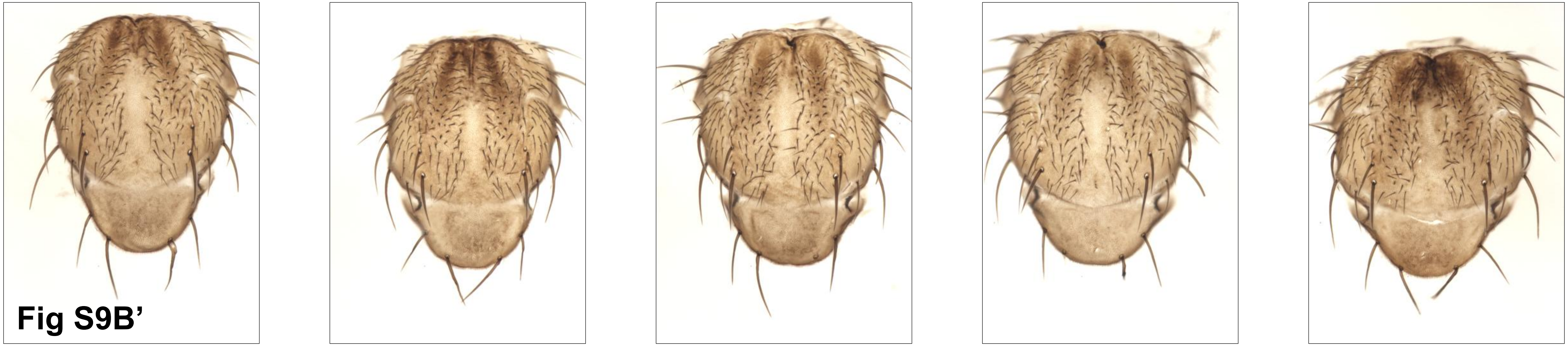

Fig S9B'

pnr>taldo-RNAi 29C

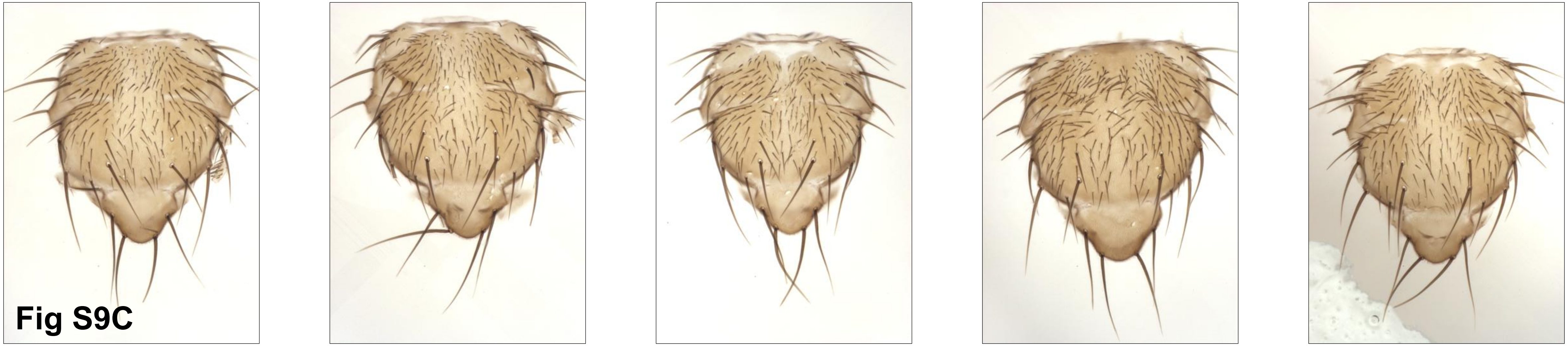

Fig S9C

pnr>wts-RNAi-1, taldo-RNAi 29C

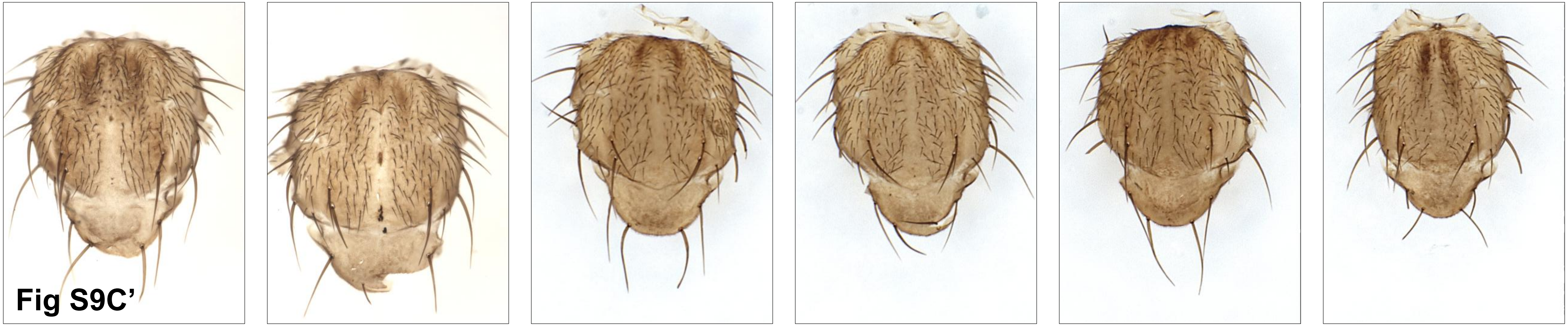

Fig S9C’

pnr-Duox-RNAi 29C

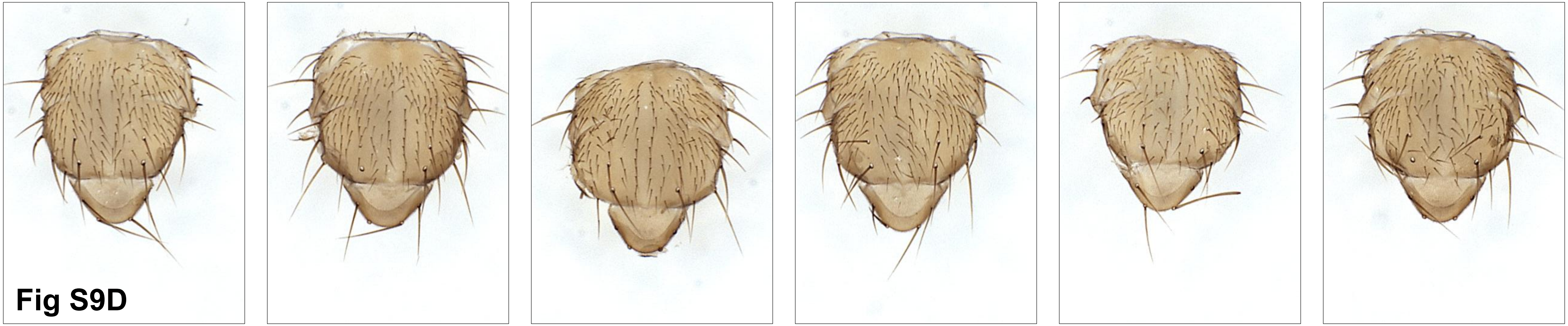

Fig S9D

pnr-wts-RNAi-1, Duox-RNAi 29C

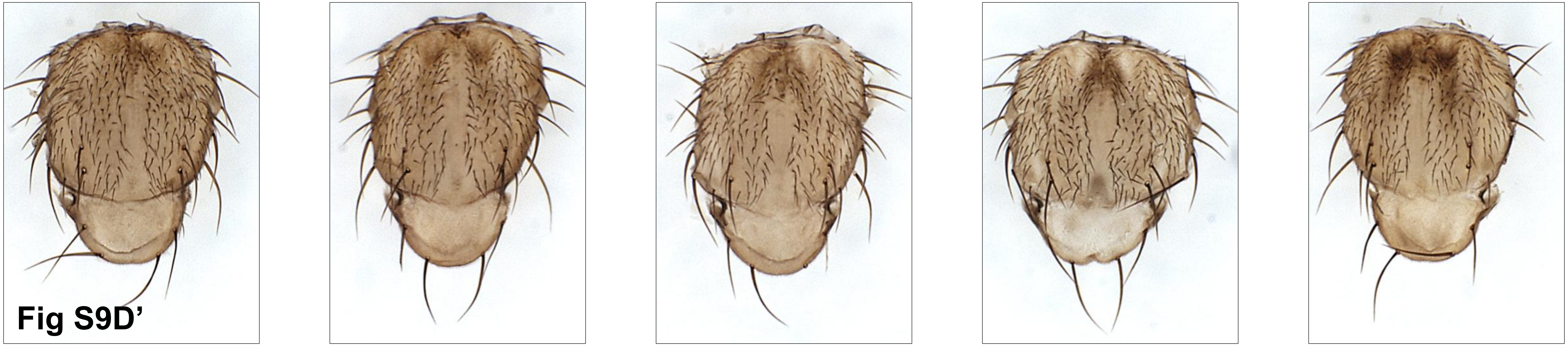

Fig S9D’

pnr>Gp150-RNAi 29C

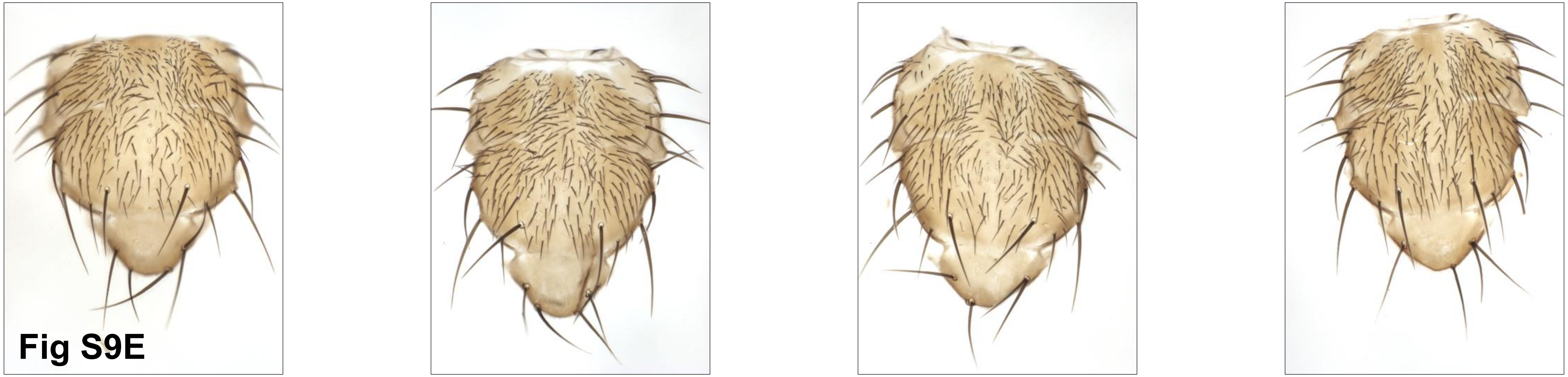

Fig S9E

pnr>wts-RNAi-1, Gp150-RNAi 29C

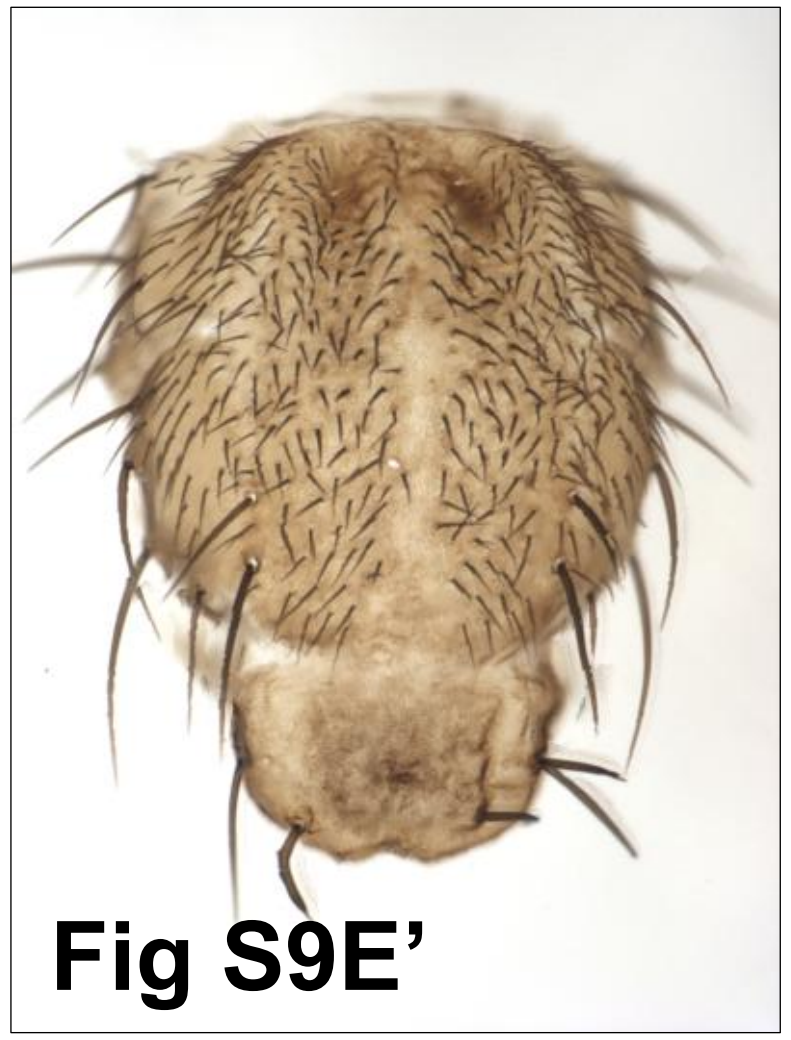

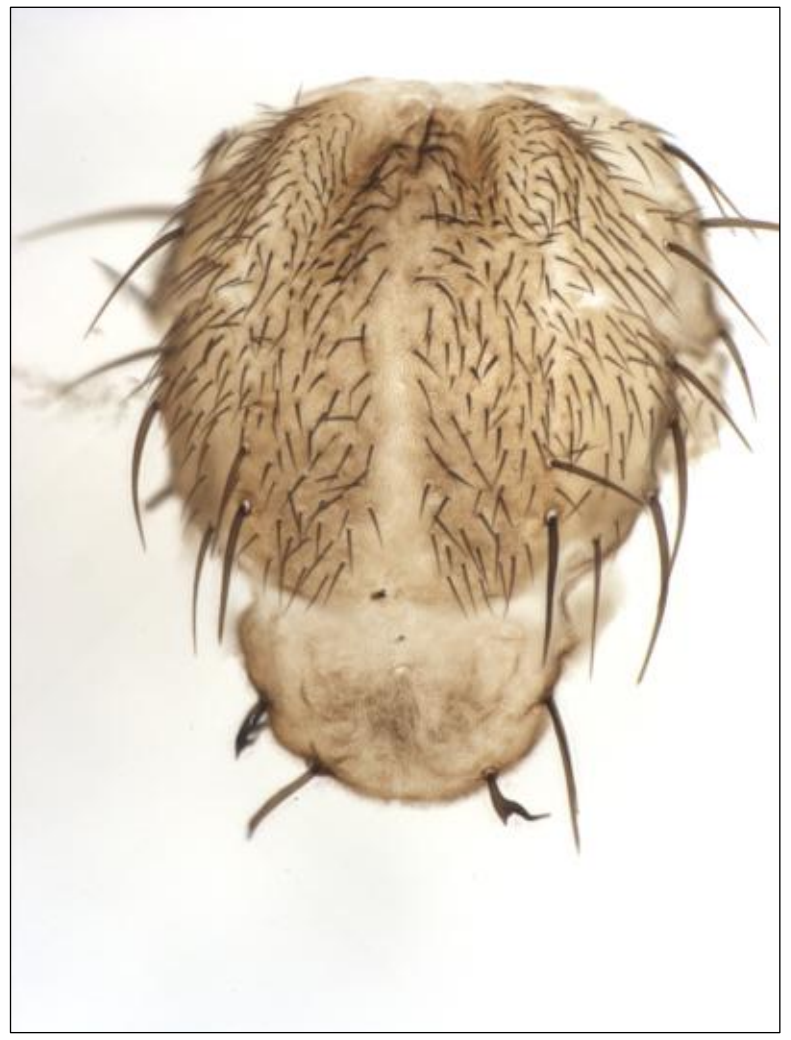

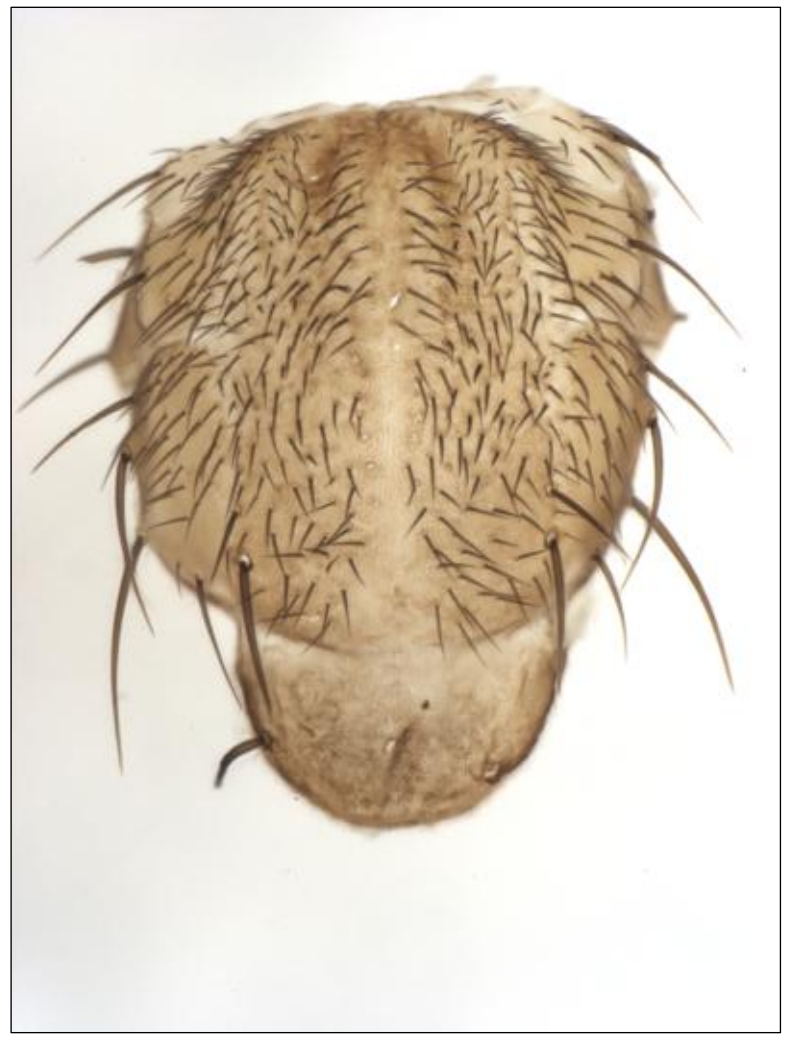

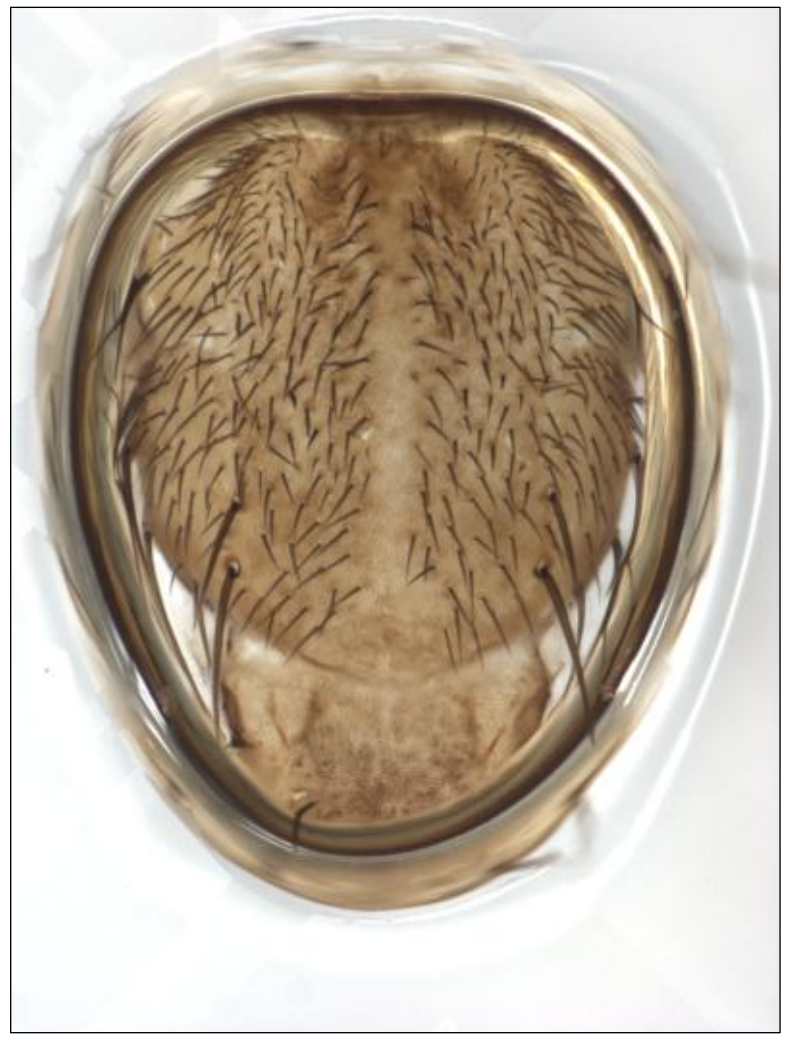

pnr-InR-RNAi 29C

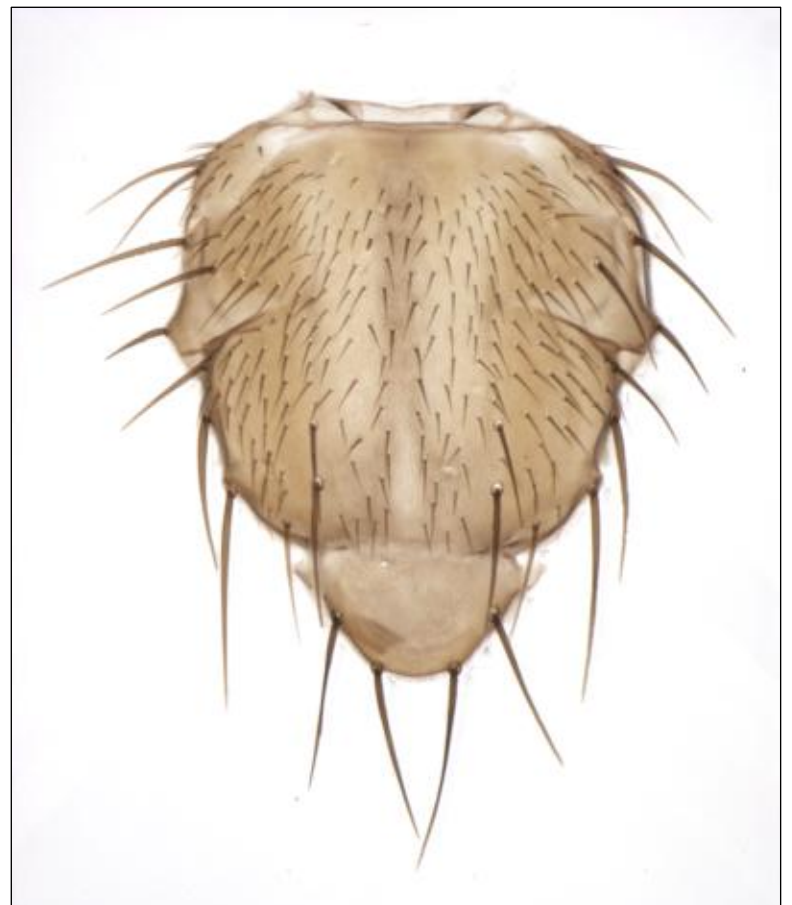

Fig S9F

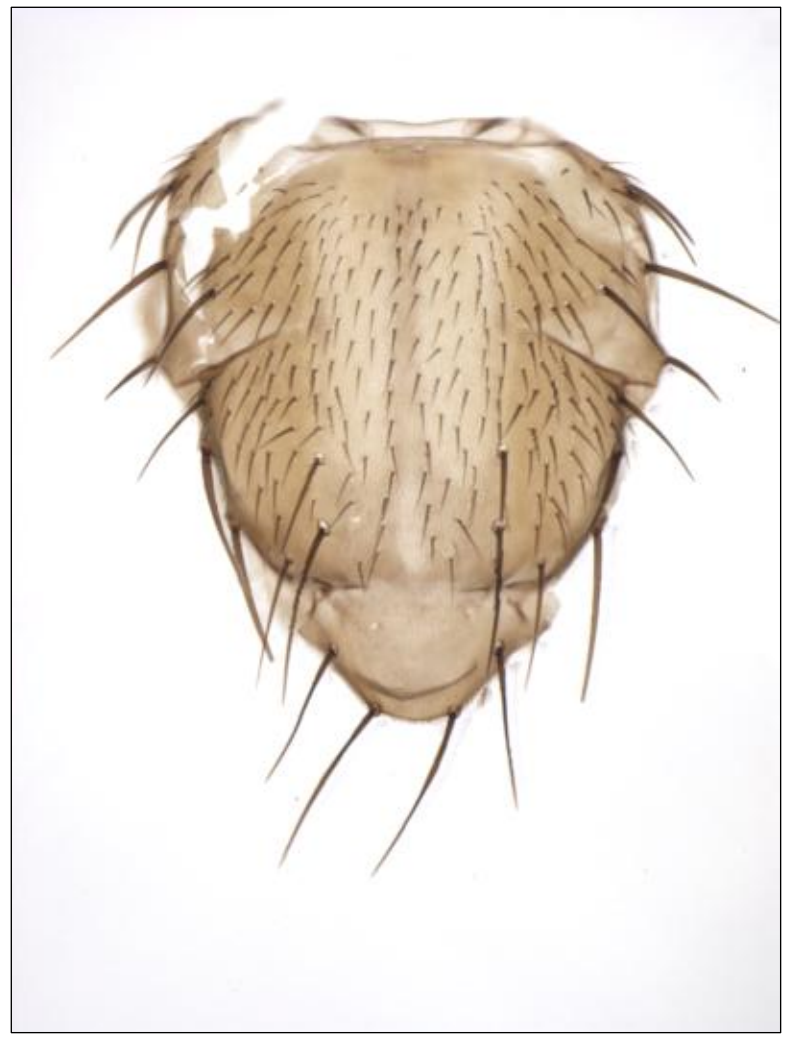

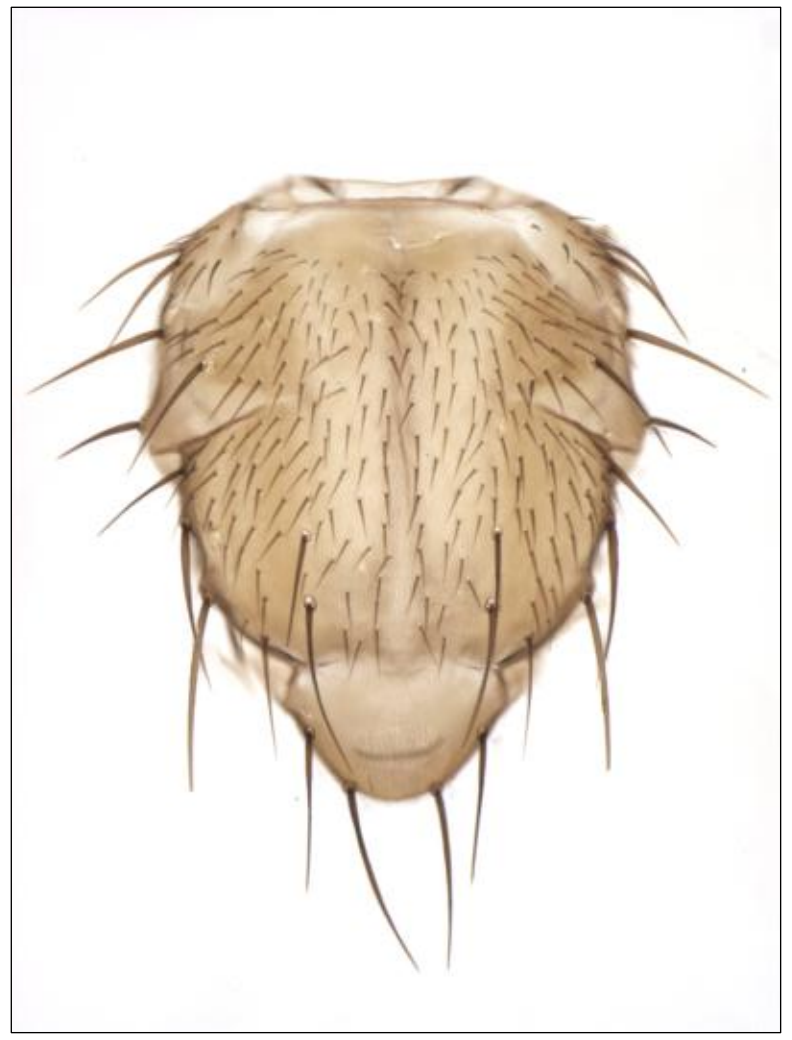

pnr>wts-RNAi-1, InR-RNAi 29C

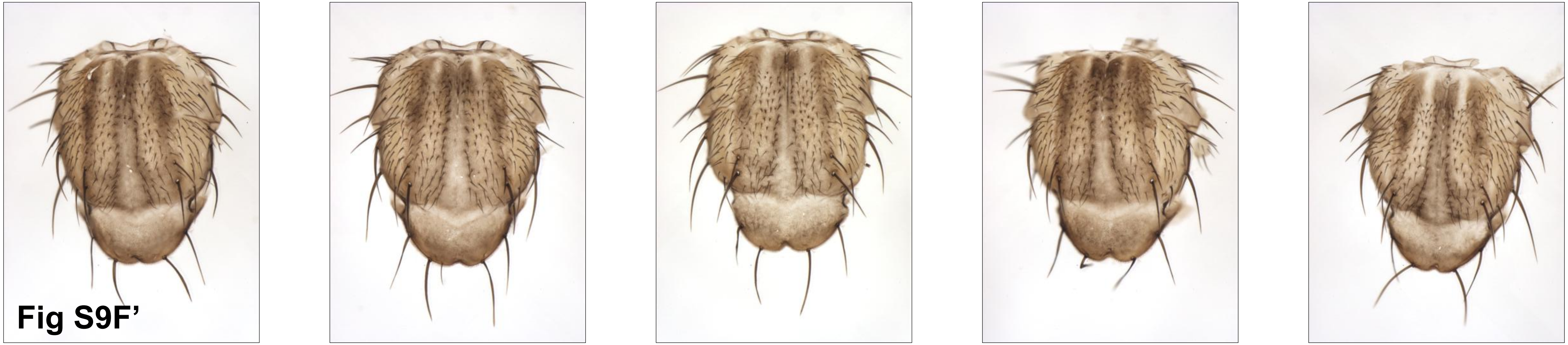

DEG non-concordant

pnr>Bsg-RNAi 29C

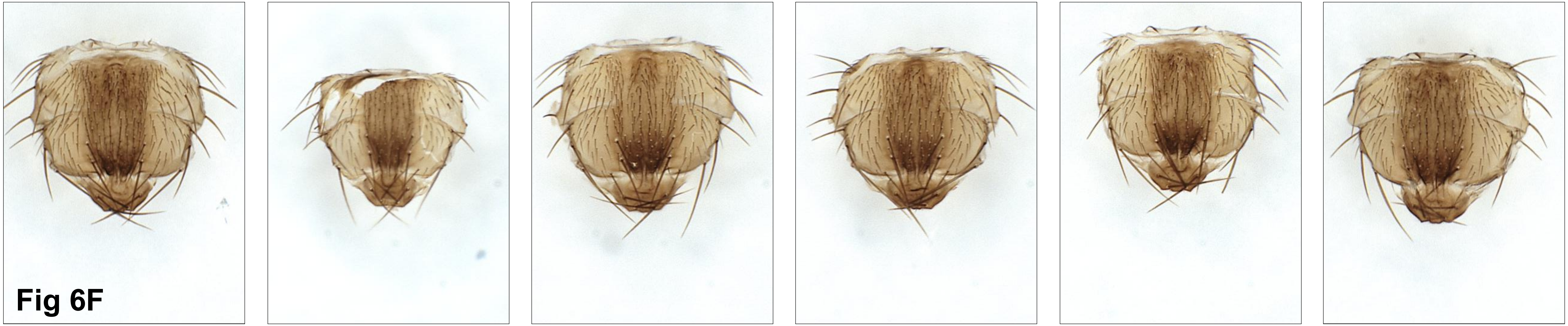

Fig 6F

pnr>wts-RNAi-1, Bsg-RNAi 29C

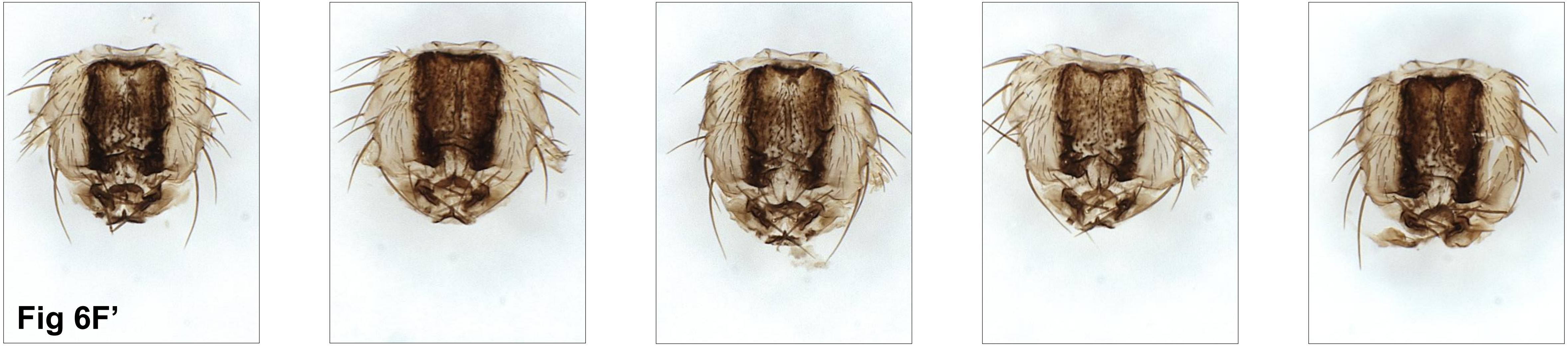

pnr>stv-RNAi 29C

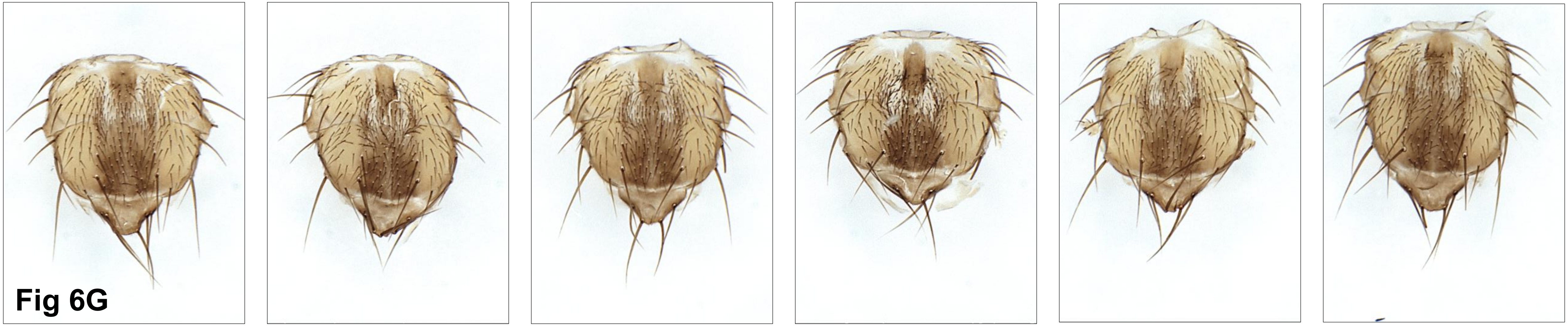

Fig 6G

pnr>wts-RNAi-1, stv-RNAi 29C

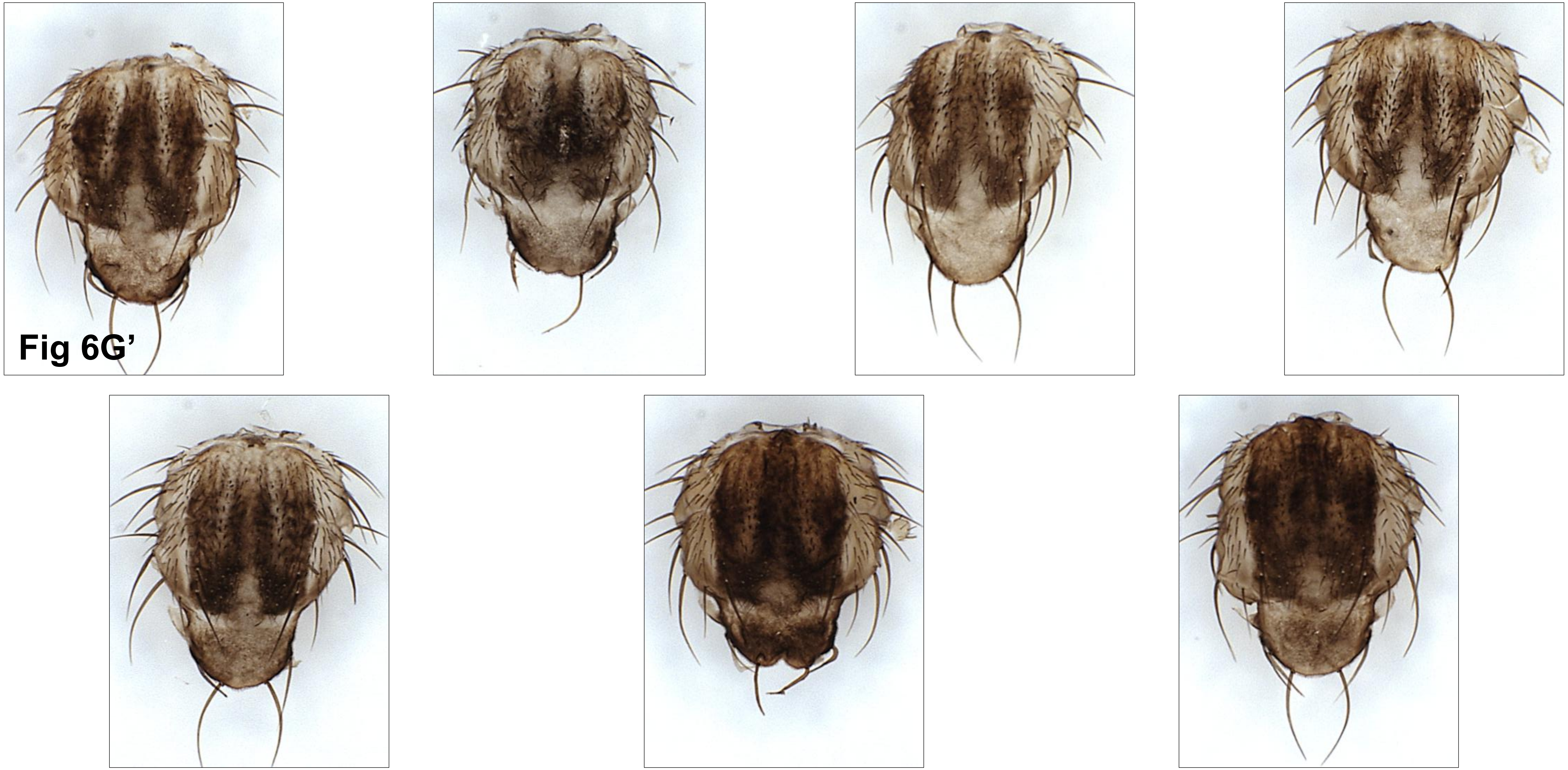

pnr>bab1-RNAi 29C

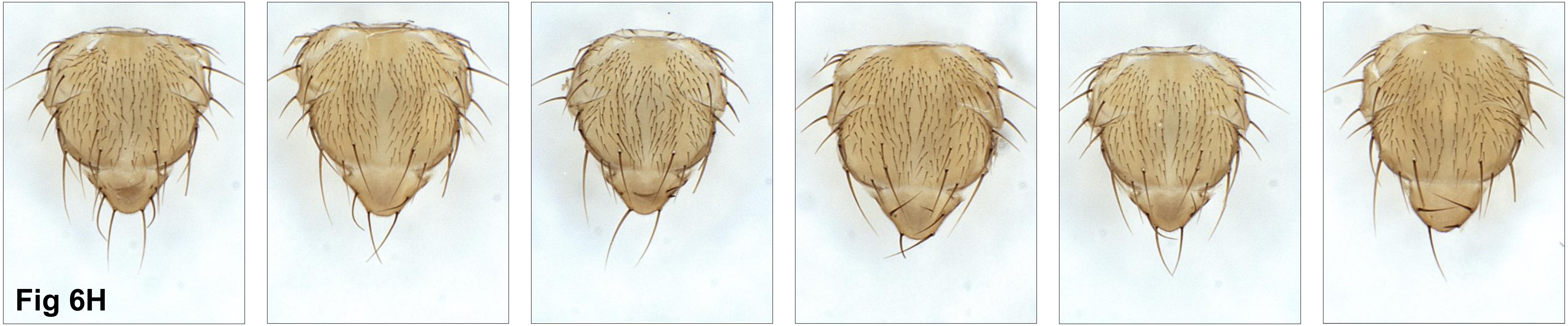

pnr>wts-RNAi-1, bab1-RNAi 29C

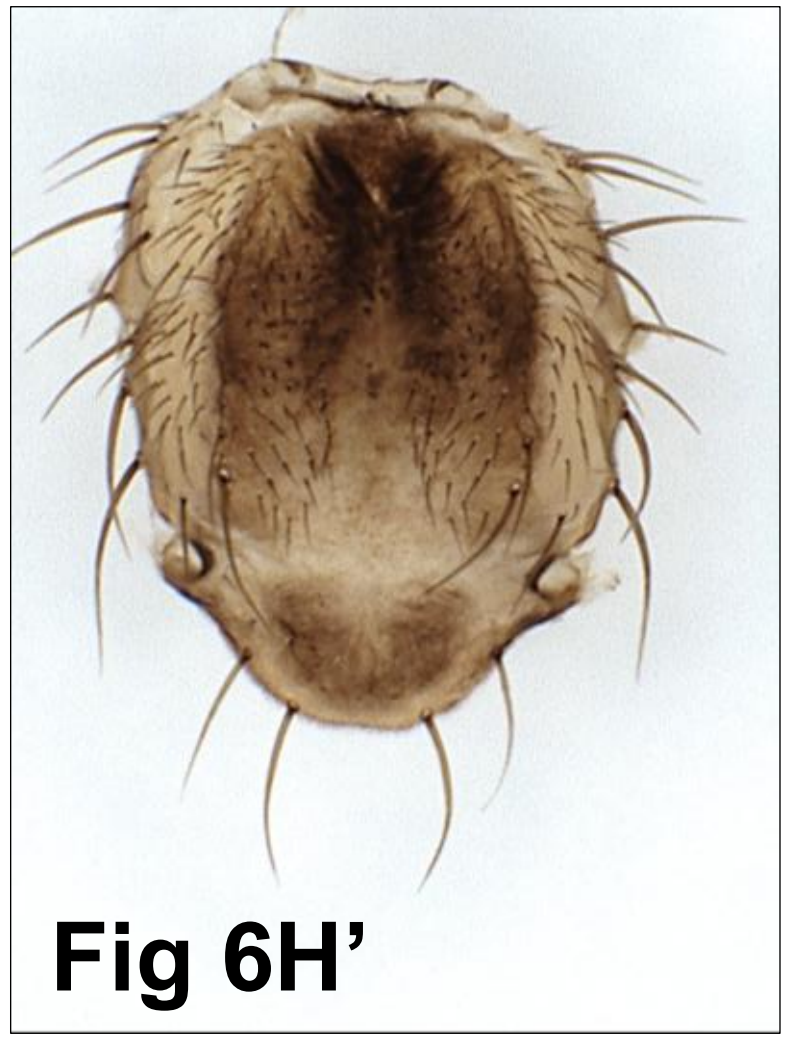

Fig 6H’

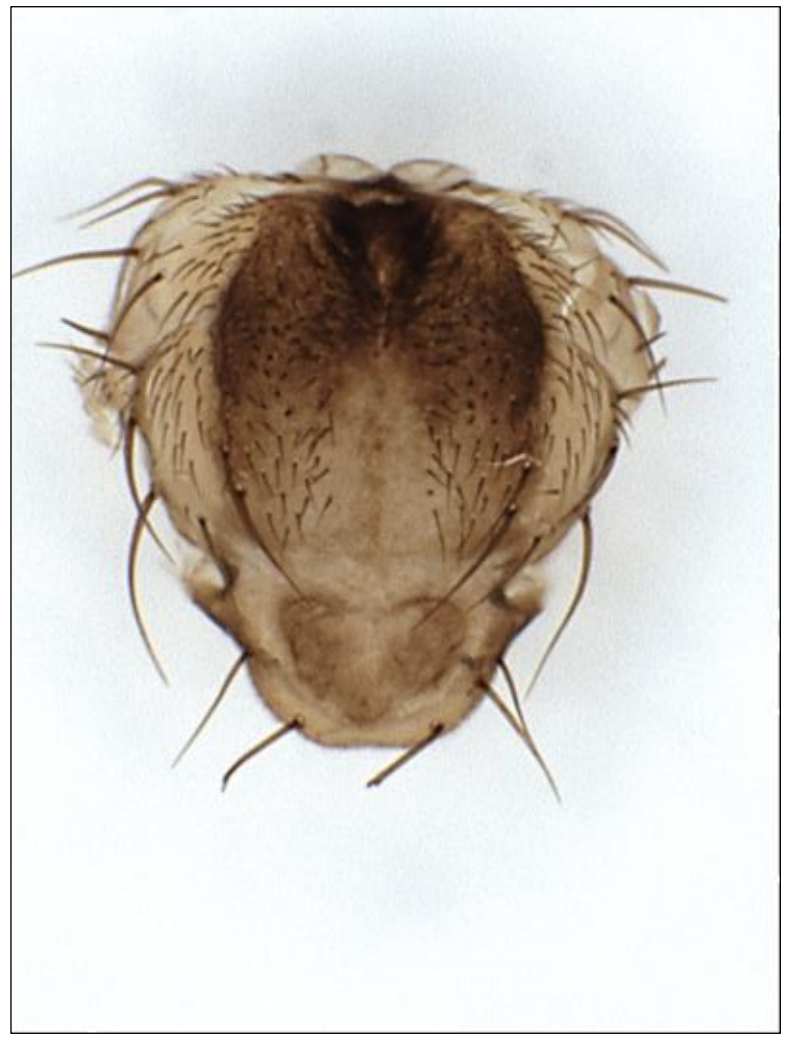

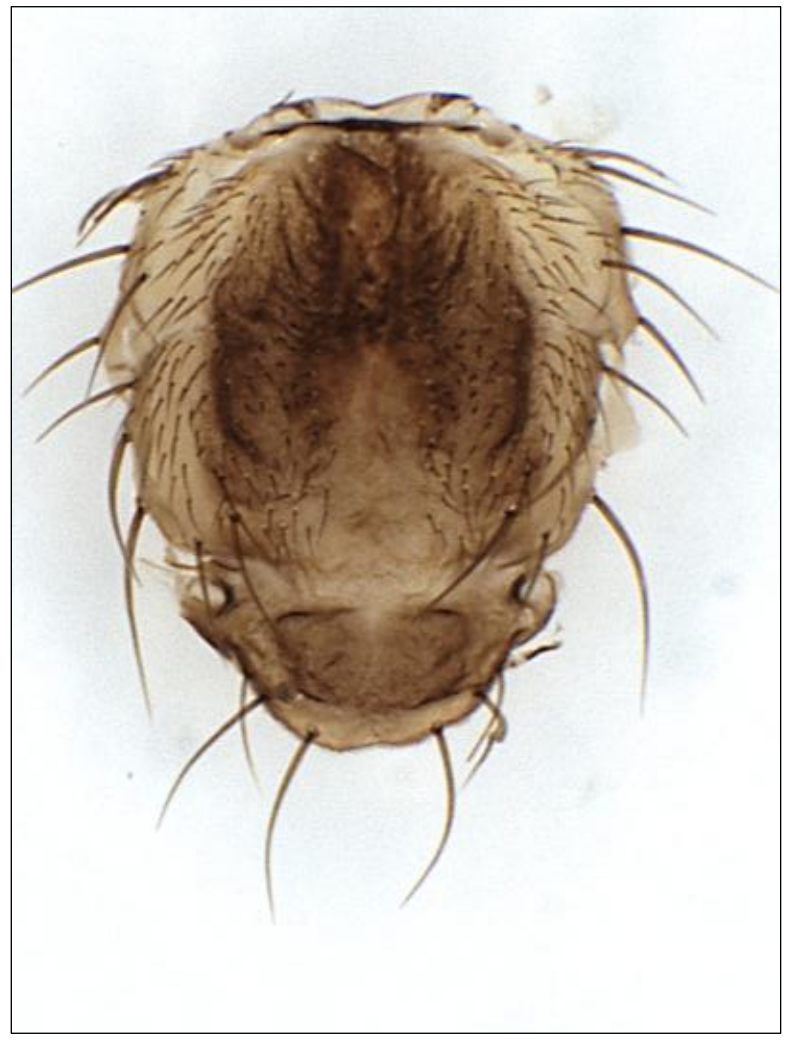

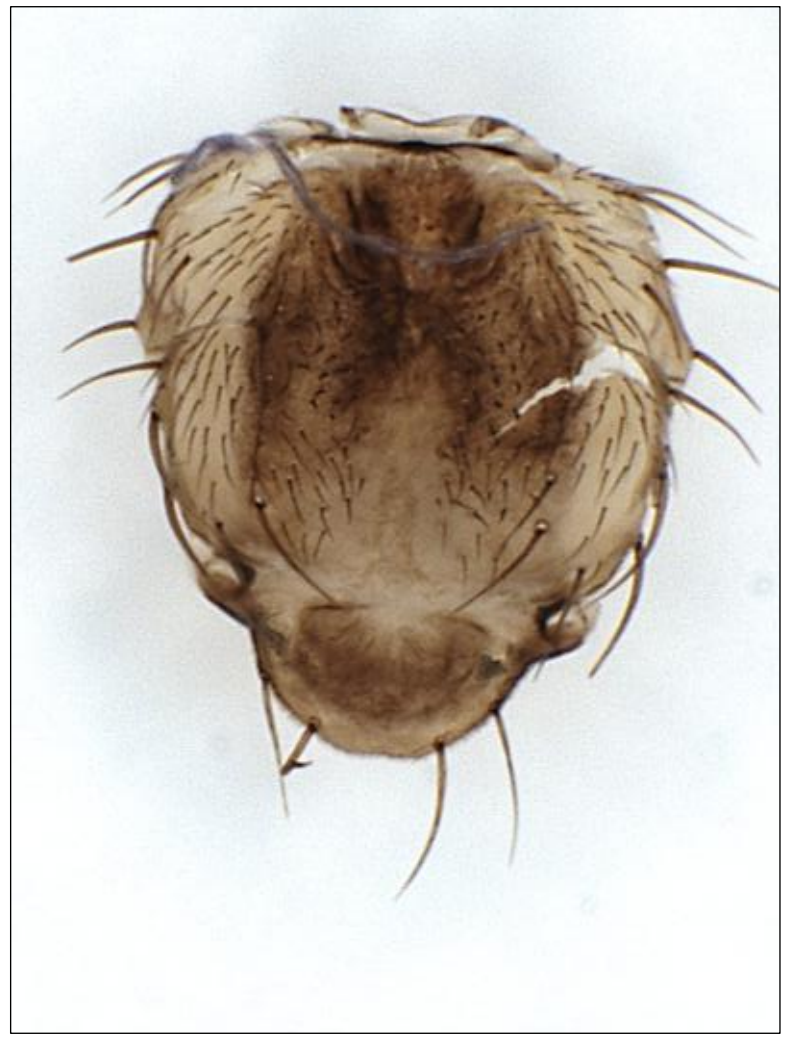

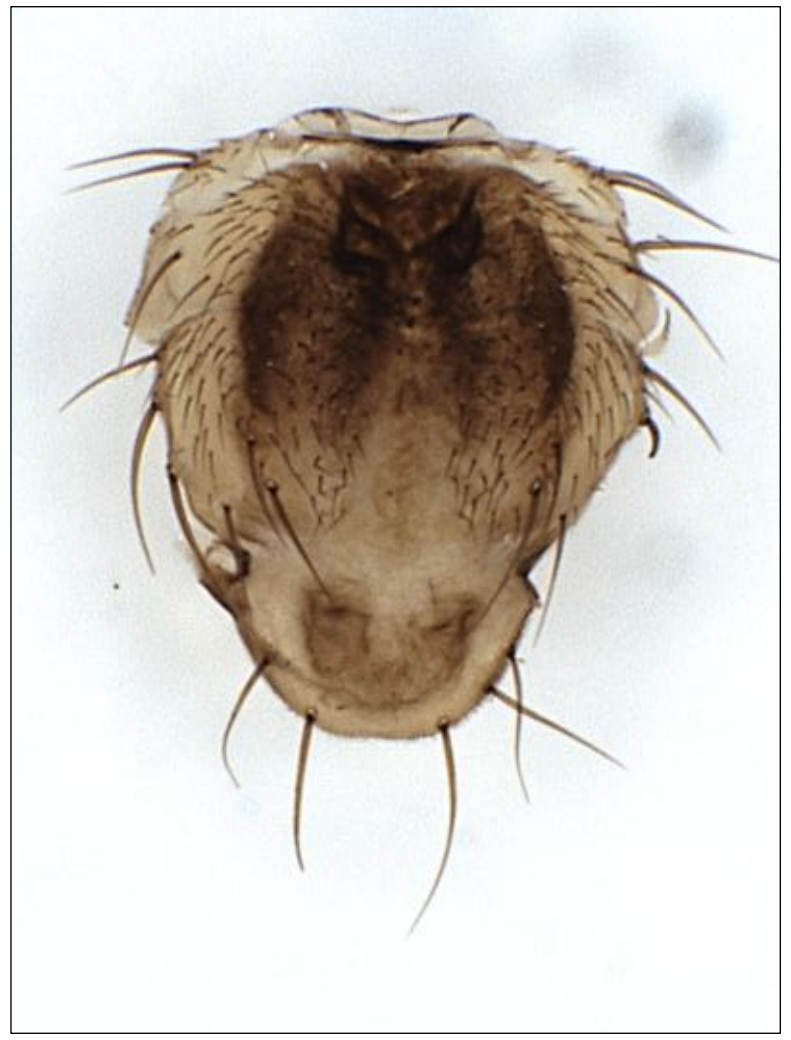

pnr>mgl-RNAi 29C

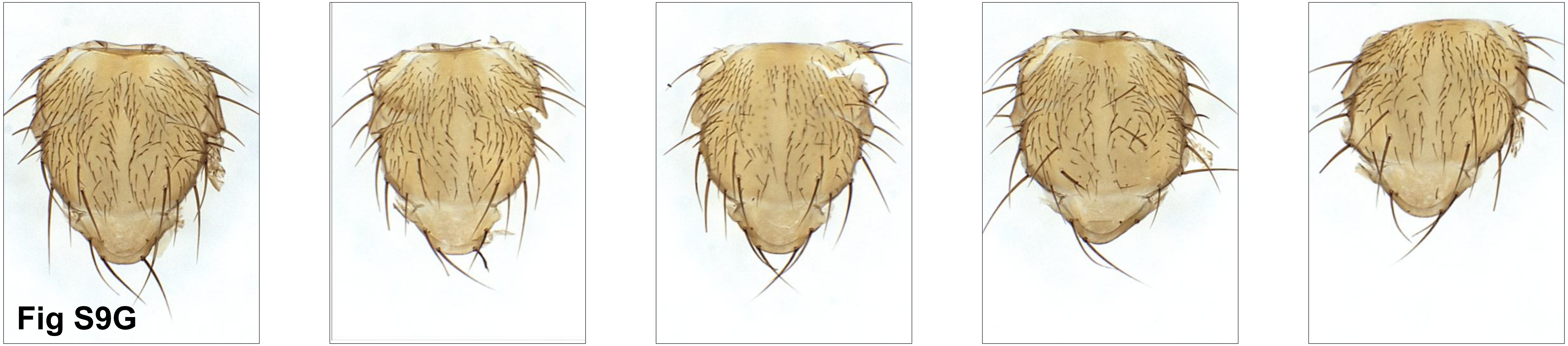

Fig S9G

pnr>wts-RNAi-1, mgl-RNAi 29C

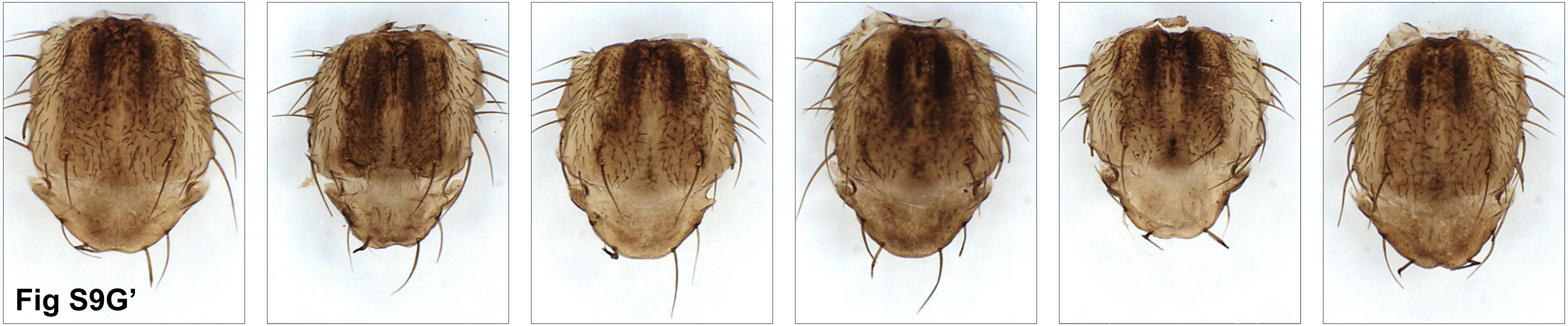

pnr>yellow-B-RNAi 29C

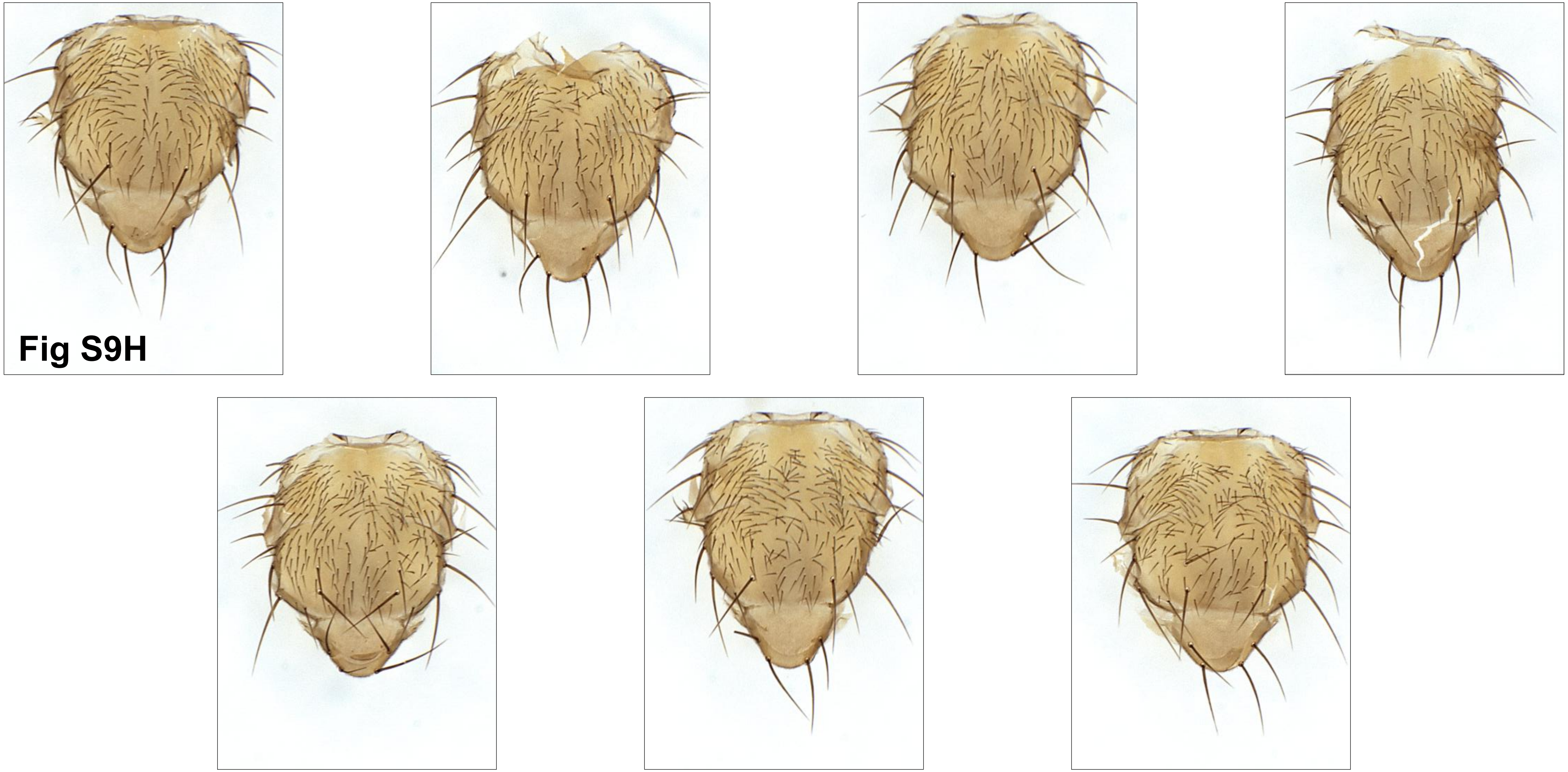

pnr> wts-RNAi-1, yellow-B-RNAi 29C

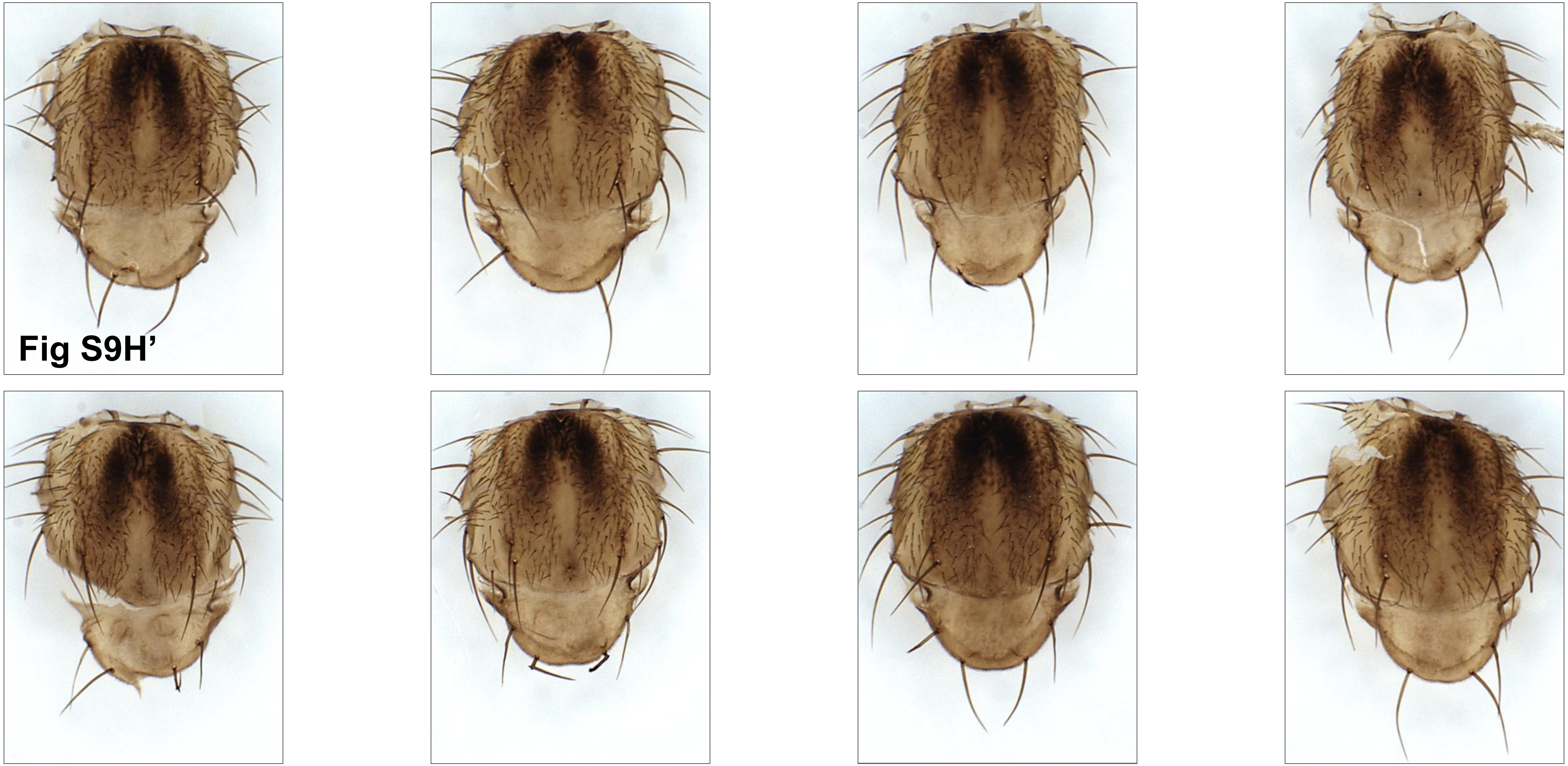

pnr>kibra-RNAi 29C

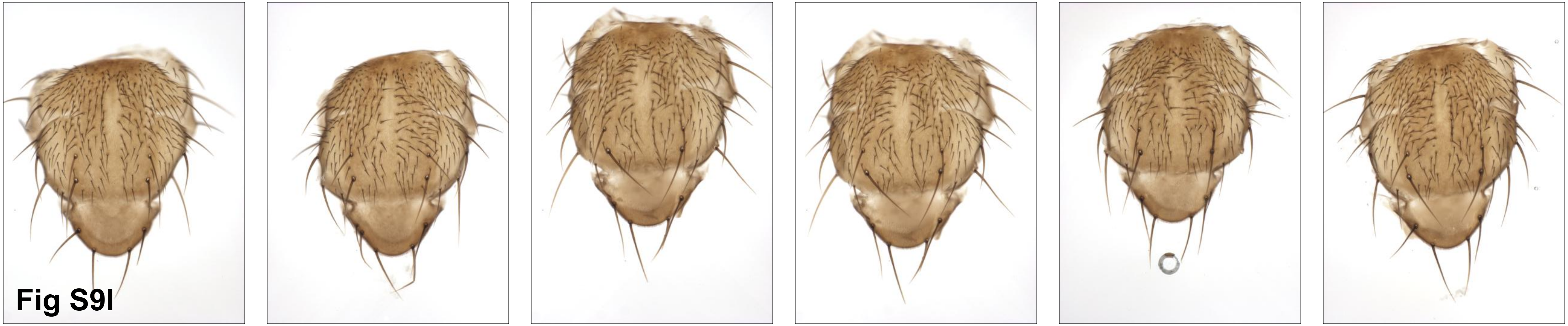

Fig S9I

pnr>wts-RNAi-1, kibra-RNAi 29C

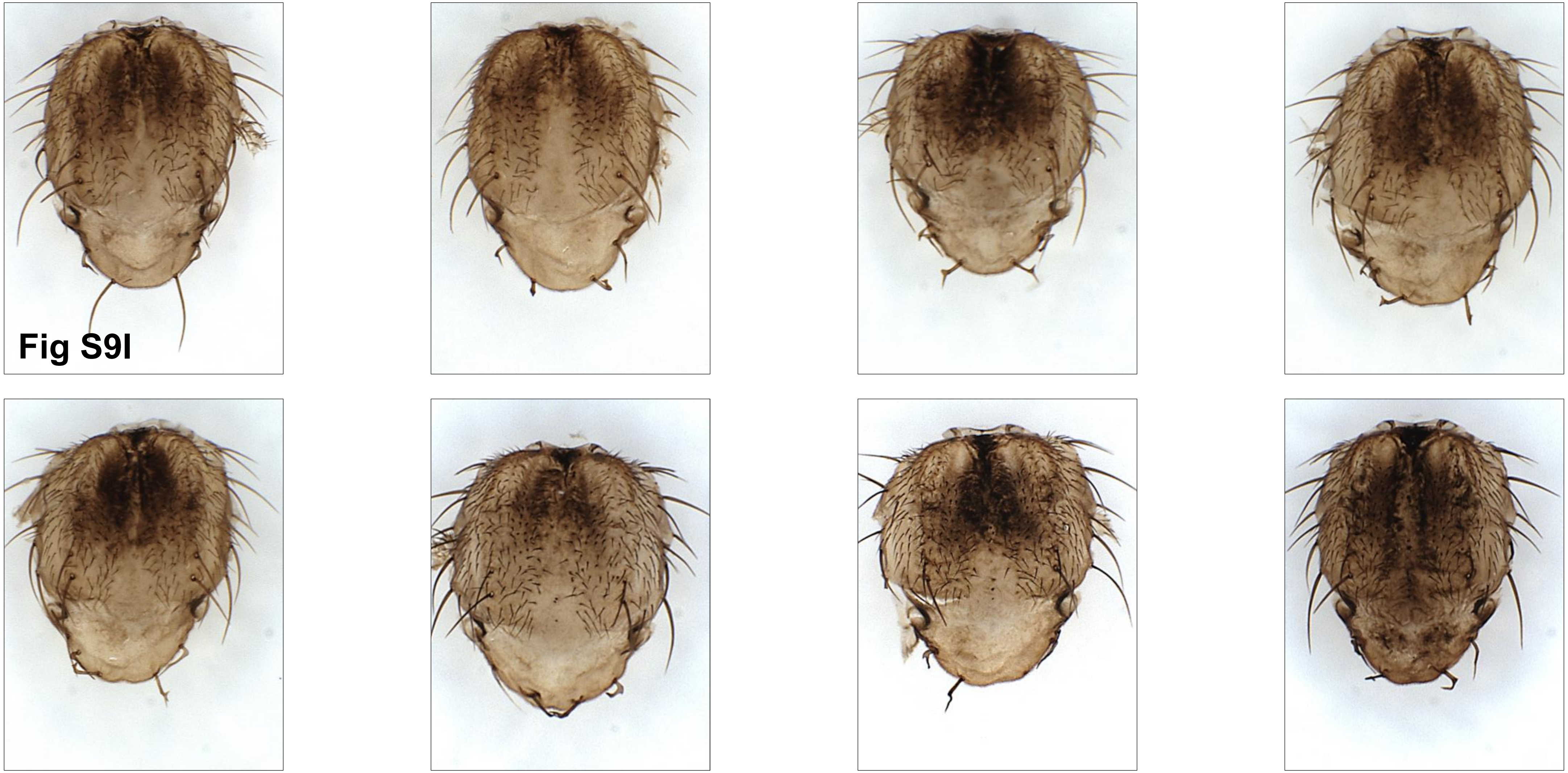

Notum Images

Supplemental Figures

pnr>ple-RNAi 29C

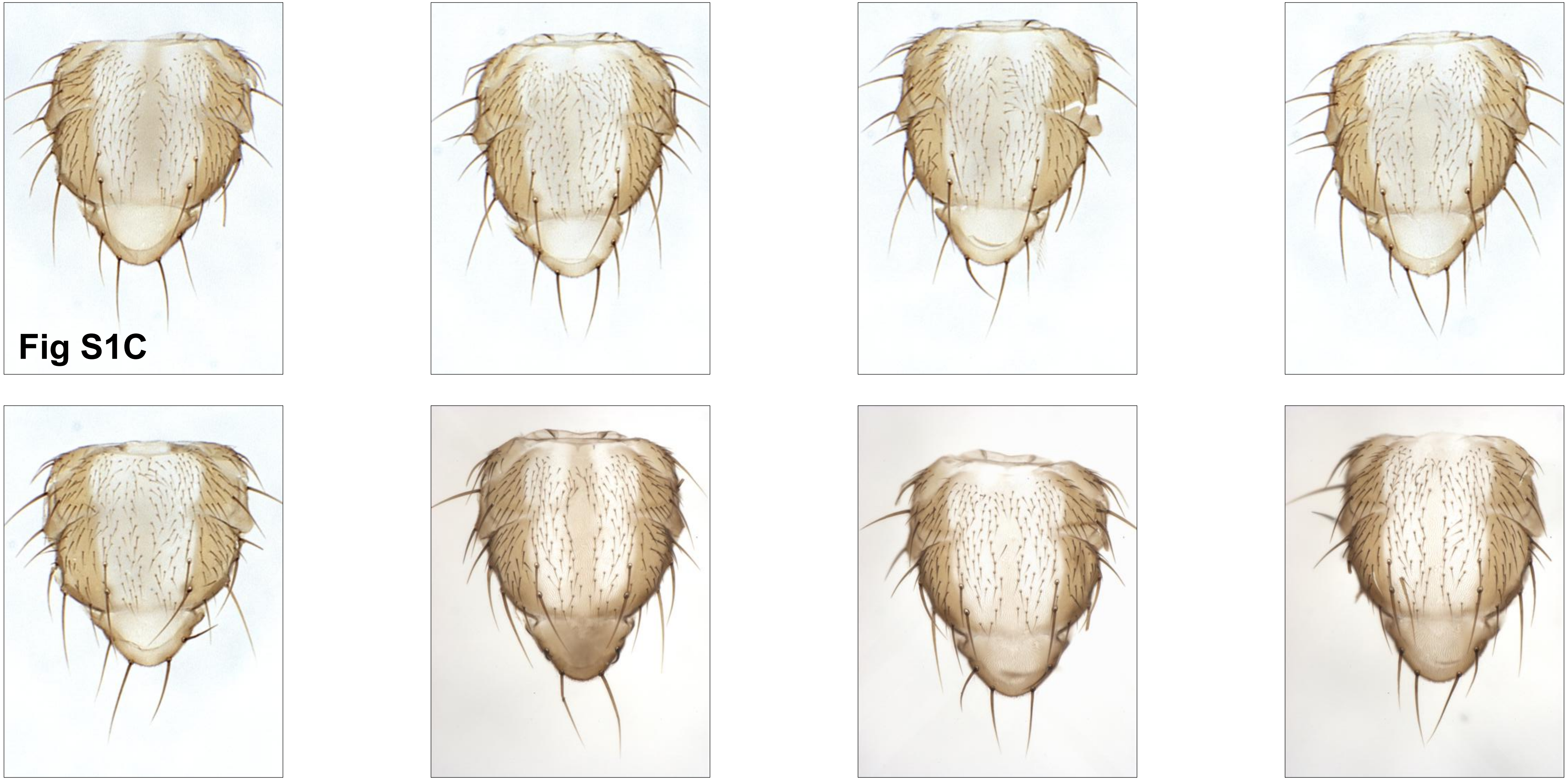

pnr>TH 29C

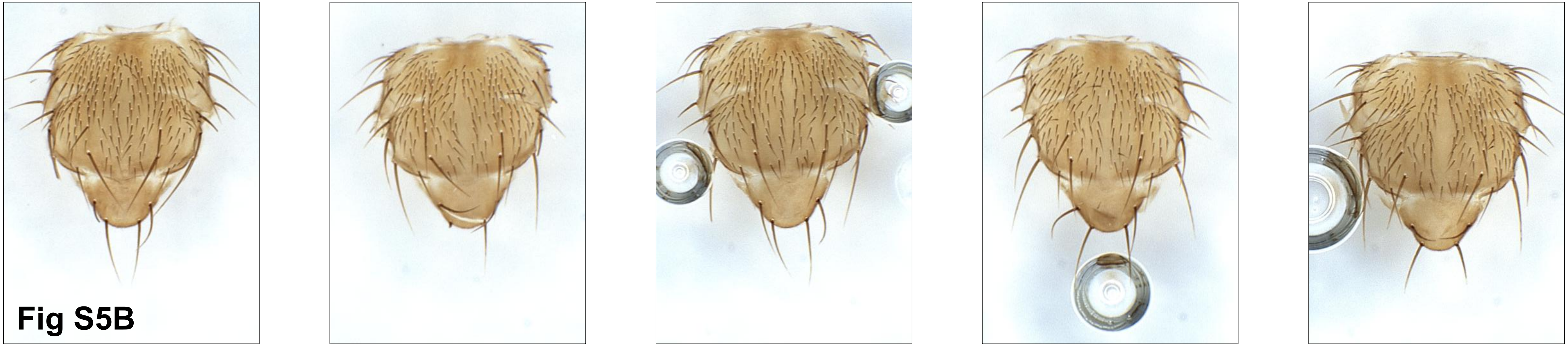

Fig S5B

pnr>yellow 29C

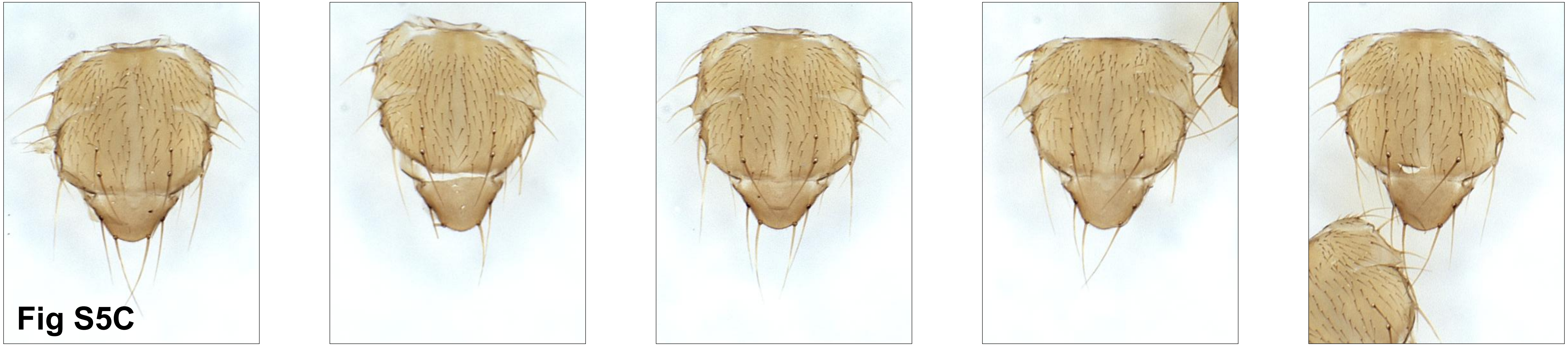

Fig S5C

pnr>TH, Ddc 29C

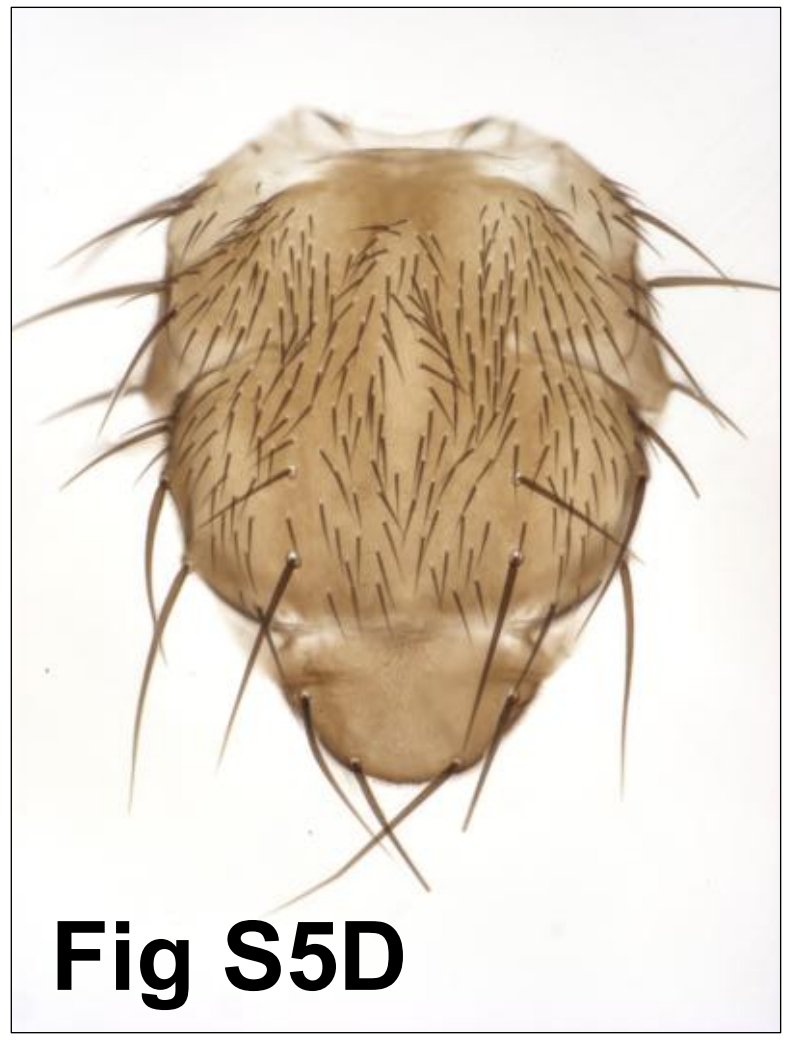

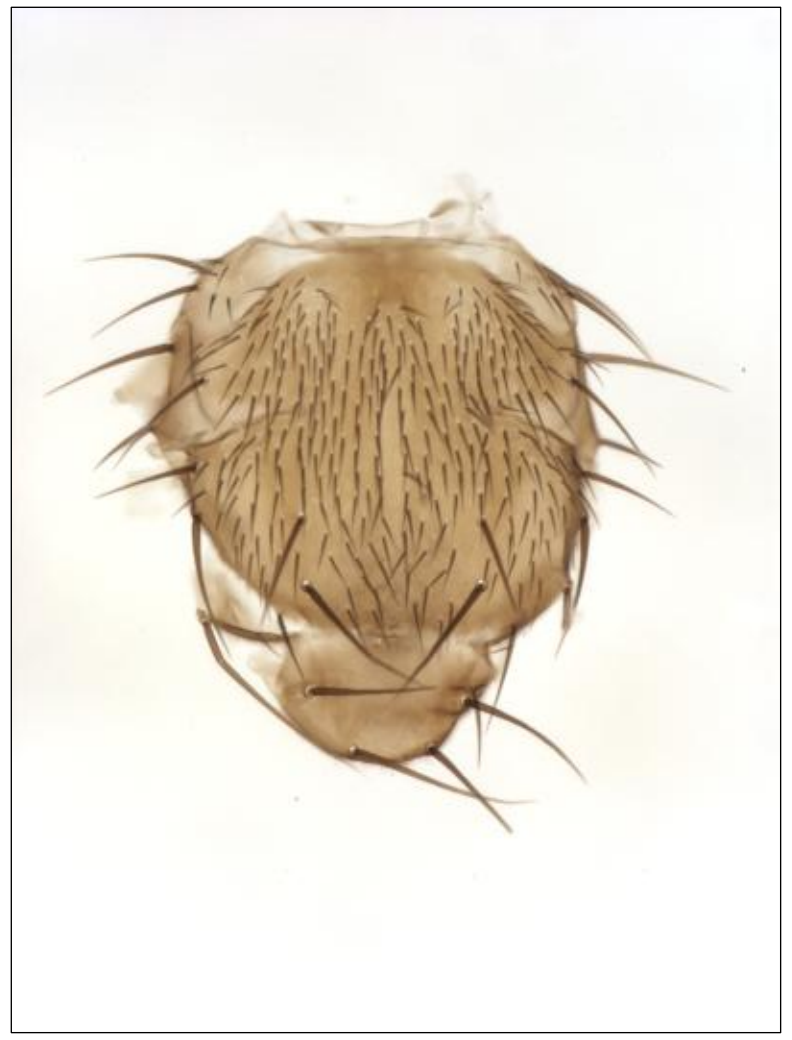

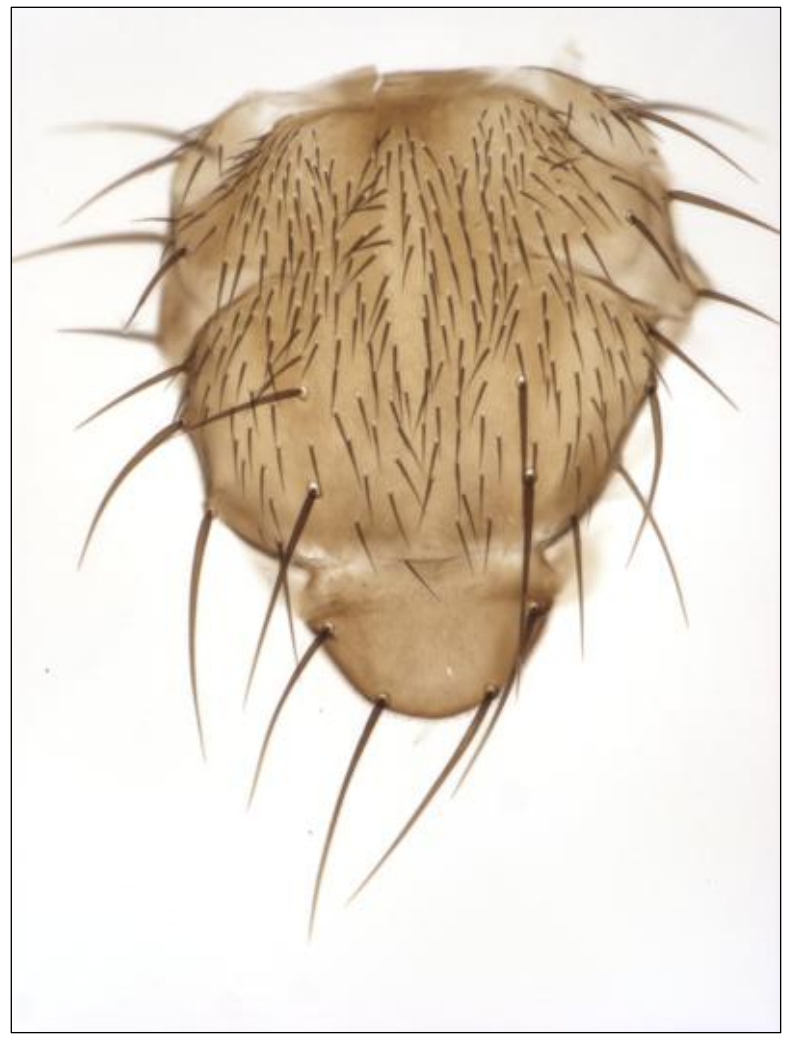

pnr>TH, yellow 29C

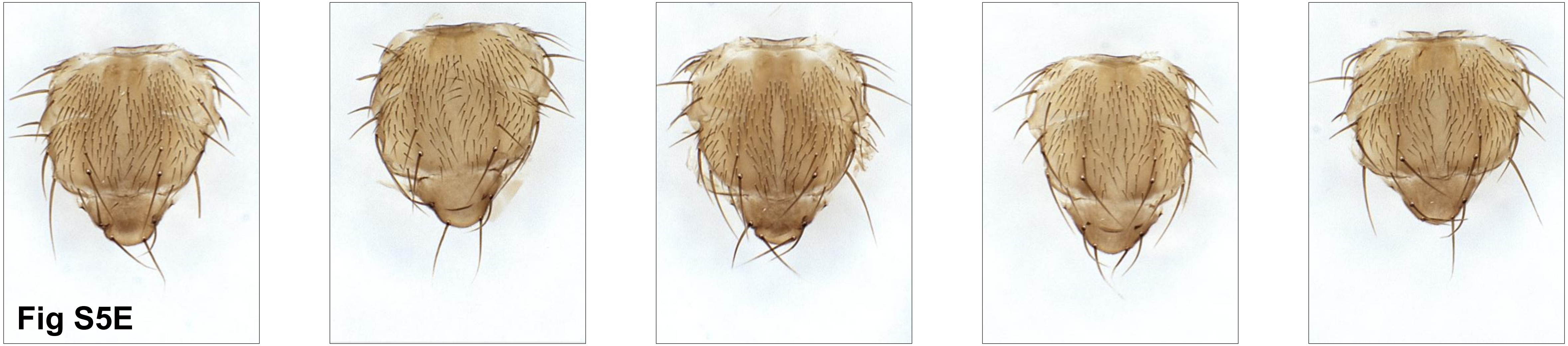

Notum Images

JNK and Growth

JNK signaling

pnr>bsk-RNAi 29C

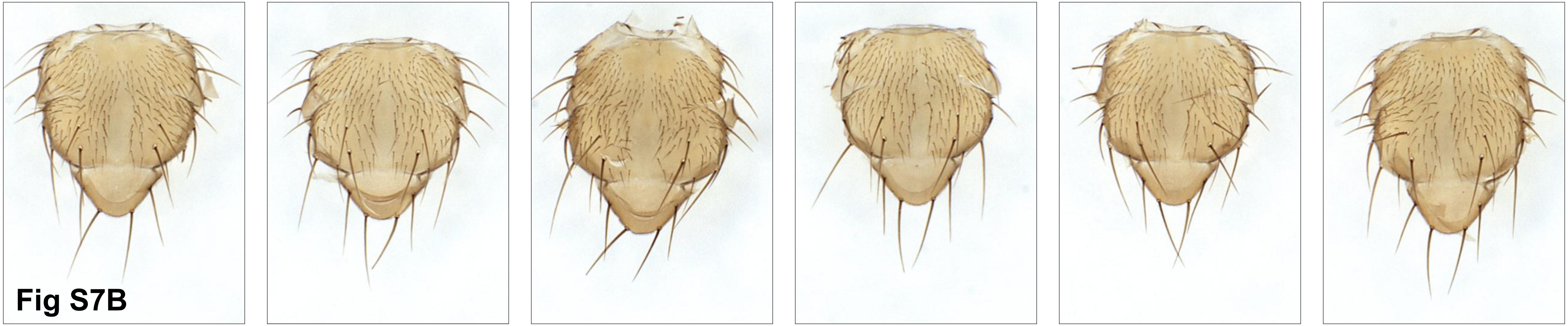

Fig S7B

pnr>bsk-RNAi, wts-RNAi-1 29C

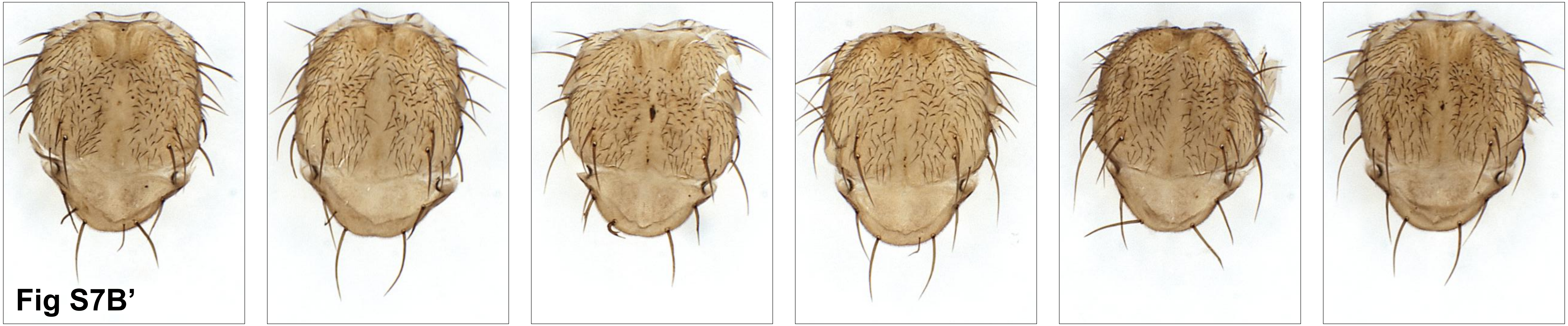

Fig S7B’

pnr>hep-RNAi 29C

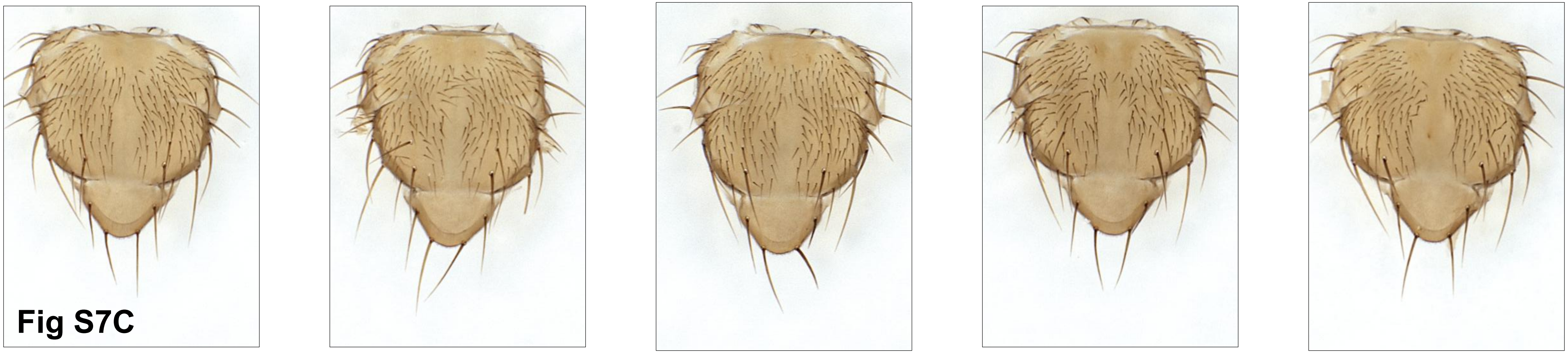

Fig S7C

pnr>hep-RNAi, wts-RNAi-1 29C

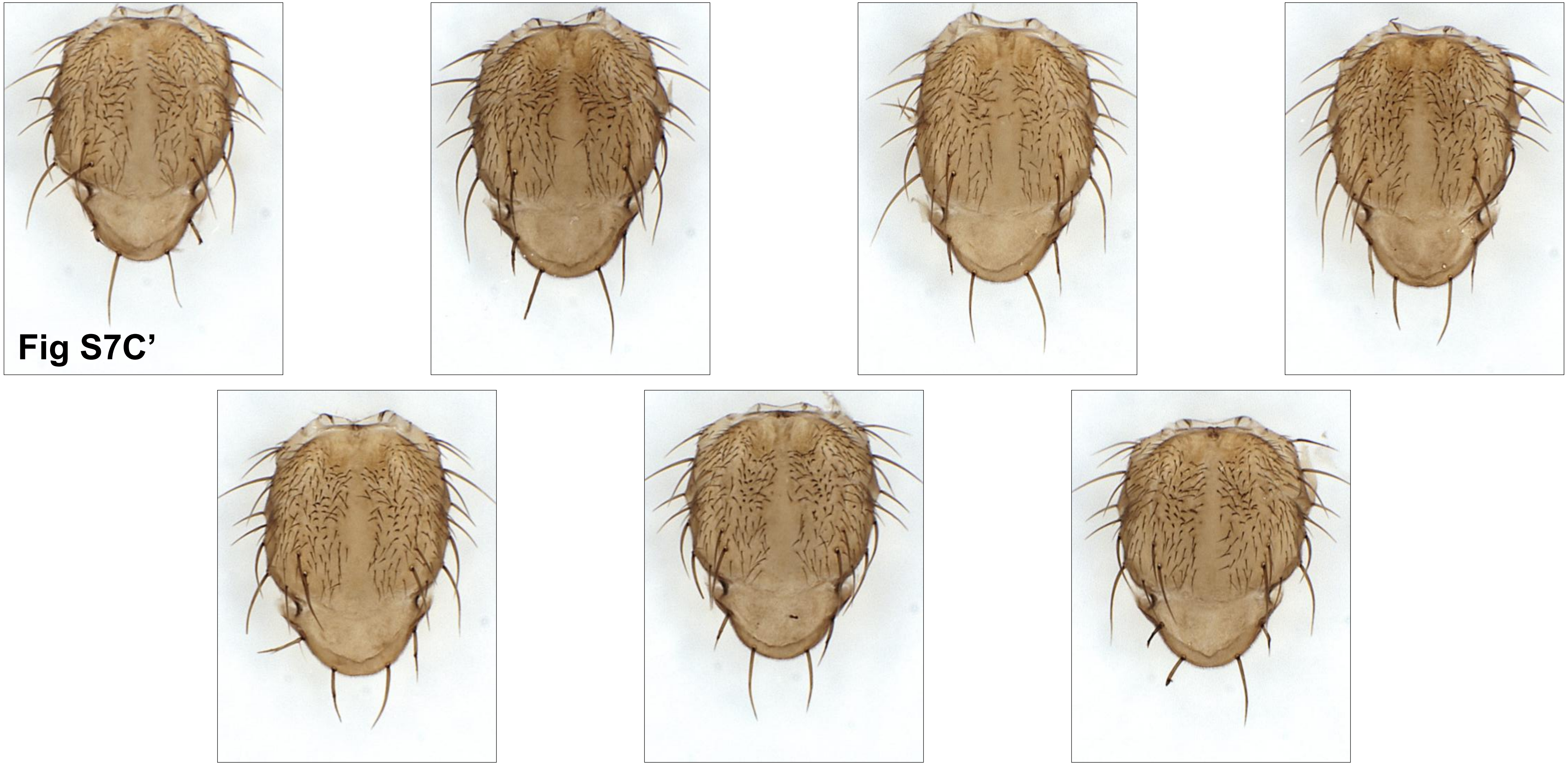

Fig S7C’

Growth

pnr>myc-RNAi-1 29C

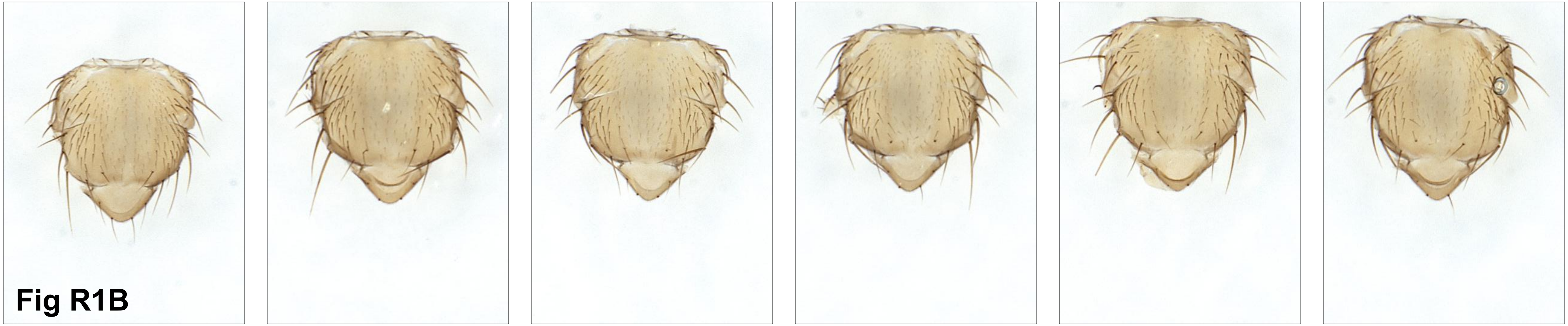

Fig R1B

pnr>myc-RNAi-1, wts-RNAi-1 29C

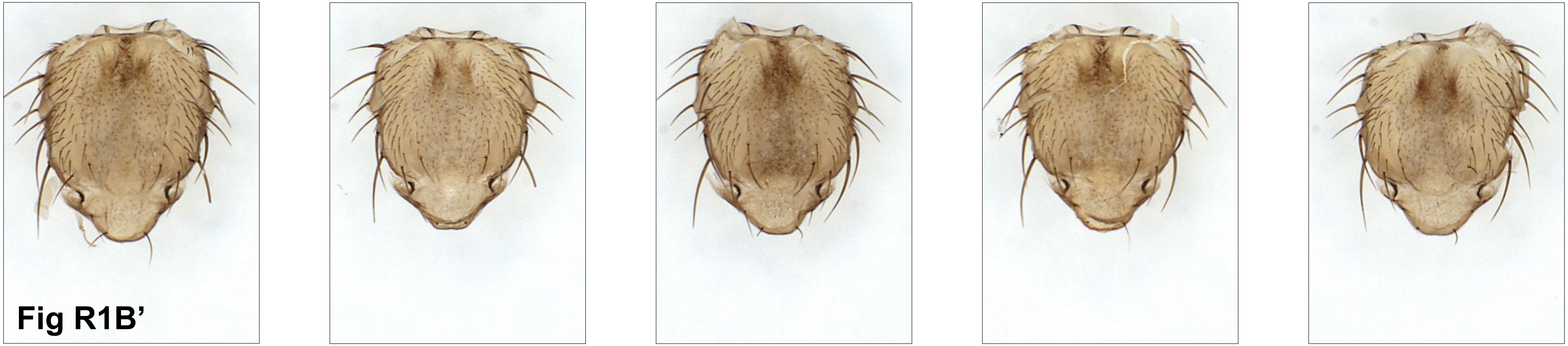

pnr>myc-RNAi-2 29C

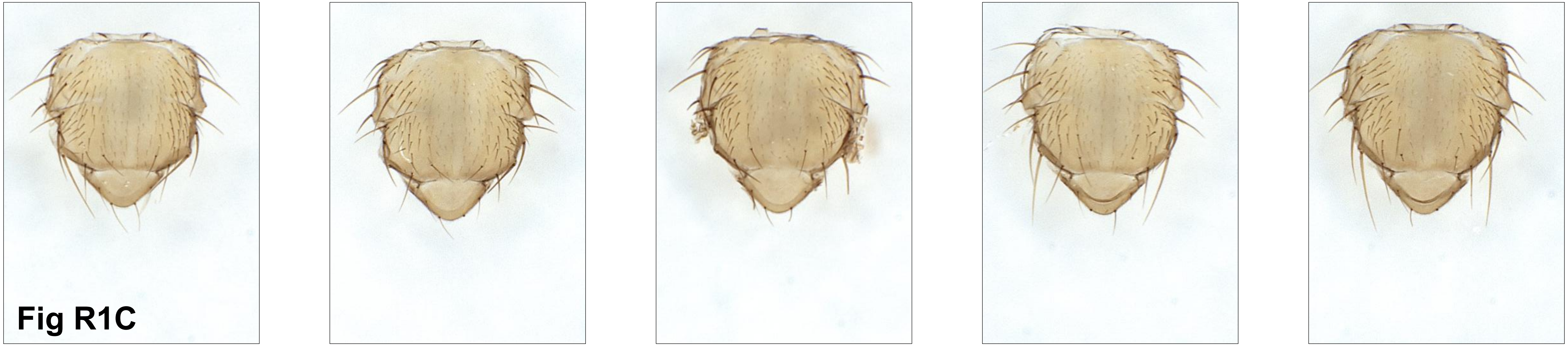

Fig R1C

pnr>myc-RNAi-2, wts-RNAi-1 29C

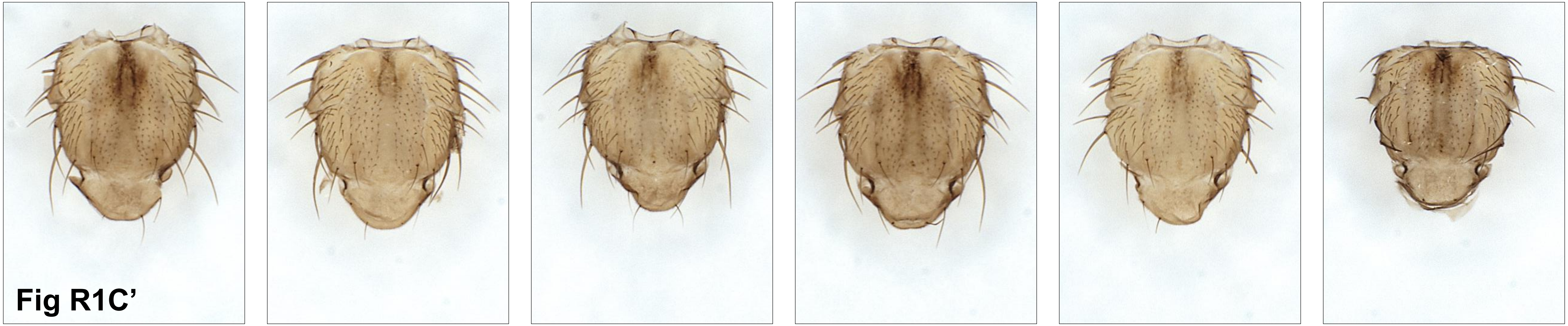

Supplement: Supplementary file 3 [file pgen.1012260.s016.pdf]
